# Long-term kidney outcomes after COVID-19: a matched cohort study using the OpenSAFELY platform

**DOI:** 10.1016/j.lanepe.2025.101338

**Published:** 2025-06-18

**Authors:** Viyaasan Mahalingasivam, Bang Zheng, Kevin Wing, Edward P.K. Parker, Krishnan Bhaskaran, Juan Jesús Carrero, Sandra Jayacodi, Edith Jumbo, Tamanna Miah, Brian Gracey, John Tazare, Shalini Santhakumaran, Rohini Mathur, Ruth E. Costello, Emily Herrett, Qing Wen, Thomas Hartney, Ian J. Douglas, Amelia Green, Louis Fisher, Helen J. Curtis, Alex J. Walker, Brian MacKenna, William J. Hulme, Amir Mehrkar, Sebastian Bacon, Ben Goldacre, Elizabeth Williamson, Dorothea Nitsch, Kathryn E. Mansfield, Laurie Tomlinson

**Affiliations:** a Department of Non-Communicable Disease Epidemiology, London School of Hygiene & Tropical Medicine, London, UK; b Department of Nephrology & Transplantation, Barts Health NHS Trust, London, UK; c Glasgow Lab for Health Data Science & AI, Public Health, School of Health & Wellbeing, University of Glasgow, Glasgow, UK; d NIHR Health Protection Research Unit in Vaccines and Immunisation, London School of Hygiene & Tropical Medicine, London, UK; e Department of Medical Epidemiology & Biostatistics, Karolinska Institutet, Solna, Sweden; f Patient and Public Involvement Partner, UK; g Department of Medical Statistics, London School of Hygiene & Tropical Medicine, London, UK; h UK Kidney Association, Bristol, UK; i Wolfson Institute of Population Health, Queen Mary University of London, London, UK; j Bennett Institute for Applied Data Science, University of Oxford, Oxford, UK; k School of Health and Care Sciences, University of Lincoln, Lincoln, UK

**Keywords:** COVID-19, Chronic kidney disease, CKD, Kidney, Kidney failure, End-stage kidney disease, End-stage renal disease, ESKD, ESRD

## Abstract

**Background:**

COVID-19 severe enough to require hospitalisation is commonly associated with acute kidney injury. However, it remains unclear whether COVID-19 leads to long-term kidney outcomes in the broader population.

**Methods:**

We undertook a population-based, matched cohort study. With the approval of NHS England, we used primary and secondary care electronic health records from England using the OpenSAFELY-TPP platform. We compared people with and without COVID-19 using fully-adjusted, stratified, cause-specific Cox models for kidney failure, 50% reduction in kidney function, and death.

**Findings:**

Overall, all outcomes were increased after COVID-19 over the course of follow-up (HR for kidney failure 1.93 [95% CI 1.84–2.03]). Hazards of kidney failure were greatest after hospitalisation (HR 7.74 [95% CI 7.00–8.56]) and remained increased beyond 180 days of follow-up. There was no evidence of increased risk in those not hospitalised (HR 0.85 [95% CI 0.79–0.90]). Increased kidney failure was more pronounced in black ethnicity (HR 4.50 [95% CI 2.92–6.92]) compared to white ethnicity (HR 1.82 [95% CI 1.71–1.94]). Amongst those hospitalised with COVID-19, there was no attenuation of kidney failure between the first wave (HR 8.74 [95% CI 6.88–11.08]) and the Omicron wave (HR 8.36 [95% CI 6.81–10.27]).

**Interpretation:**

We observed increased long-term kidney outcomes in people hospitalised with COVID-19, as well as notable ethnic differences. Our results suggest strategies to minimise severe COVID-19 should continue to be optimised among vulnerable groups, and that kidney function should be proactively monitored after hospital discharge.

**Funding:**

10.13039/501100000272National Institute for Health and Care Research.

**Research in context**

**Evidence before this study**

We searched MEDLINE with no language restrictions for journal articles using the terms “SARS-CoV-2”, “COVID-19”, “coronavirus”, “kidney outcomes”, “renal outcomes”, “nephropathy”, “chronic kidney disease”, “CKD”, “renal insufficiency”, “end-stage renal disease”, “ESRD”, “end-stage kidney disease”, “ESKD”, “end-stage renal failure”, “ESRF”, “kidney failure” and “renal failure” between 1 January 2021 and 5 December 2024. This search produced 3117 results, from which we identified ten articles investigating long-term kidney outcomes after COVID-19. We identified an additional ten articles through hand searches from reference lists.

Of the twenty articles reviewed, eight investigated longer-term kidney function or diagnosed kidney disease after COVID-19 related acute kidney injury (AKI) or specifically those who received kidney replacement therapy in the intensive care unit setting. These were mainly single centre follow-up studies from early in the COVID-19 pandemic. Most studies found poorer renal outcomes in survivors with AKI compared to those without AKI. One study of a five centre US health system compared outcomes after COVID-19-related-AKI with AKI related to influenza and other causes, finding a greater annual decline in kidney function after COVID-19.

Most of the remaining articles investigated kidney disease as one of several long-term outcomes after COVID-19, predominantly using large electronic health record (EHR) databases, with one single-centre study follow-up of hospitalised COVID-19 patients from China and one self-reported questionnaire in Norway. Most defined kidney disease on the basis of incident ICD-10 codes, while one investigated for incident estimated glomerular filtration rate (eGFR) <90 ml/min/1.73 m^2^. Two studies investigated kidney failure as an outcome: one found increased hazards of kidney failure after COVID-19 (hazard ratio (HR) 1.69 (95% confidence interval (CI) 1.14–2.50)), with the other from US veterans’ data finding persistently increased hazards of kidney failure after COVID-19 compared to influenza from before the COVID-19 pandemic.

Two studies used EHR data to systematically investigate kidney outcomes at the population level, including people not hospitalised for COVID-19. The first is an analysis of US veterans’ data, up to April 2021, finding increased kidney outcomes including kidney failure and 50% reduction in eGFR after COVID-19, including non-hospitalised COVID-19. The second is an analysis using Swedish data which found accelerated decline in eGFR after COVID-19 compared to pre-pandemic pneumonia. Other smaller studies which reported on individuals not hospitalised for COVID-19 were at high risk of bias in both in terms of selection and outcome classification.

**Added value of this study**

In contrast to US veterans' data of predominantly older males, this study investigated long-term COVID-19 outcomes using linked primary and secondary care data from over 3.5 million COVID-19 survivors and 10 million comparators, drawn from the primary care population in a setting with universal healthcare coverage. We reported outcomes from later in the pandemic, including periods in which the Omicron variant was dominant and after mass vaccination, and we used a range of epidemiological methods to address potential biases. Like other studies, we found increased kidney outcomes after COVID-19 but in contrast to the US veterans’ study, we did not find increased kidney outcomes amongst individuals with COVID-19 who were not hospitalised. We found that most of the increase in kidney failure after COVID-19 was in people who required dialysis around the time of their COVID-19 illness and important ethnic inequalities in kidney outcomes, with markedly increased HRs for kidney failure in black ethnic groups.

**Implications of all the available evidence**

The evolving evidence base on long-term kidney outcomes after COVID-19 is of significant importance for policymakers in considering the future burden of chronic kidney disease, as well as being able to plan for future pandemics. There is a strong association between COVID-19 severe enough to require hospitalisation and long-term adverse kidney outcomes, especially amongst those who had AKI. However, in our large study, we reassuringly did not find any evidence of worse kidney outcomes amongst people not requiring hospitalisation. These findings may be factored into considerations about preventing hospitalisation in groups who are most at risk, as well as emphasising the importance of routinely following-up kidney function after hospital discharge for COVID-19 in order to ensure timely management of chronic kidney disease. Further research is required into the reasons behind ethnic differences in kidney outcomes, potentially driven by inequalities in healthcare and social determinants of health before infection, and differences or delays to COVID-19 management after infection. It remains unknown whether kidney outcomes after COVID-19 hospitalisation are causally specific to COVID-19 or whether they are a consequence of severe illness more generally.

## Introduction

At the start of the COVID-19 pandemic, hospitals across the world observed that severe infection was often accompanied by acute kidney injury (AKI), in many cases requiring life-sustaining dialysis, and sometimes resulting in chronic kidney disease (CKD) or kidney failure.^1^, ^2^, ^3^, ^4^, ^5^ Advanced CKD places considerable strain on health systems but its burden in the asymptomatic early stages may be undetected.^6^, ^7^, ^8^ Long-term kidney-related consequences of COVID-19 need to be understood to inform renal service and future-pandemic planning.

Using Swedish population level data, we previously found an association between COVID-19 and accelerated decline in kidney function compared to pre-pandemic pneumonia, particularly amongst survivors of hospitalisation.^9^ However, the long-term burden of kidney outcomes after COVID-19 remains unknown. An analysis of US military veteran survivors from early in the pandemic demonstrated heightened risks, even in those who were not hospitalised.^5^ However, it is unlikely that these findings from predominantly older men are generalisable to the wider population and it remains unknown whether risks persisted into an era of widespread vaccination and milder illness.

We aimed to comprehensively investigate the burden of long-term kidney outcomes following COVID-19 up to December 2022. We conducted a study using routinely-collected electronic health record (EHR) data from across England comparing the risks of kidney failure, reduction in kidney function, and death in a matched analysis of individuals with and without recorded COVID-19. In a secondary analysis, we compared kidney outcomes between adults hospitalised with COVID-19, and those hospitalised with pneumonia between February 2017 and December 2019.

## Methods

### Data source

We used linked EHR data from primary and secondary care, and COVID-19 test data. All data were linked, stored, and analysed securely using the OpenSAFELY platform (https://www.opensafely.org/) as part of the National Health Service (NHS) England OpenSAFELY COVID-19 service (Appendix S1). Primary care records managed by the software provider TPP, were linked to hospital admissions data from the Secondary Uses Service (SUS) and COVID-19 testing data from the Second Generation Surveillance System (SGSS) through OpenSAFELY. OpenSAFELY-TPP holds EHR data for 24 million people registered with primary care practices using TPP SystmOne software, representing around 40% of the population of England.^10^ Pseudonymised data include coded diagnoses, medications, and physiological parameters. No free text data are included.

### Study design and populations

We conducted a population-based, matched (age, sex, region, date) cohort study. The study population comprised individuals without pre-existing kidney failure (i.e., record of dialysis or kidney transplantation or estimated glomerular filtration rate (eGFR) < 15 ml/min/1.73 m^2^) registered with primary care practices in OpenSAFELY-TPP. We excluded individuals with missing age, sex, sustainability and transformation partnership (STP) (local NHS administrative regions), or Index of Multiple Deprivation (IMD) data.^11^ Individuals were also required to have at least three months of follow-up available before cohort entry to ensure reliable capture of baseline health status.

**Fig. 1:**
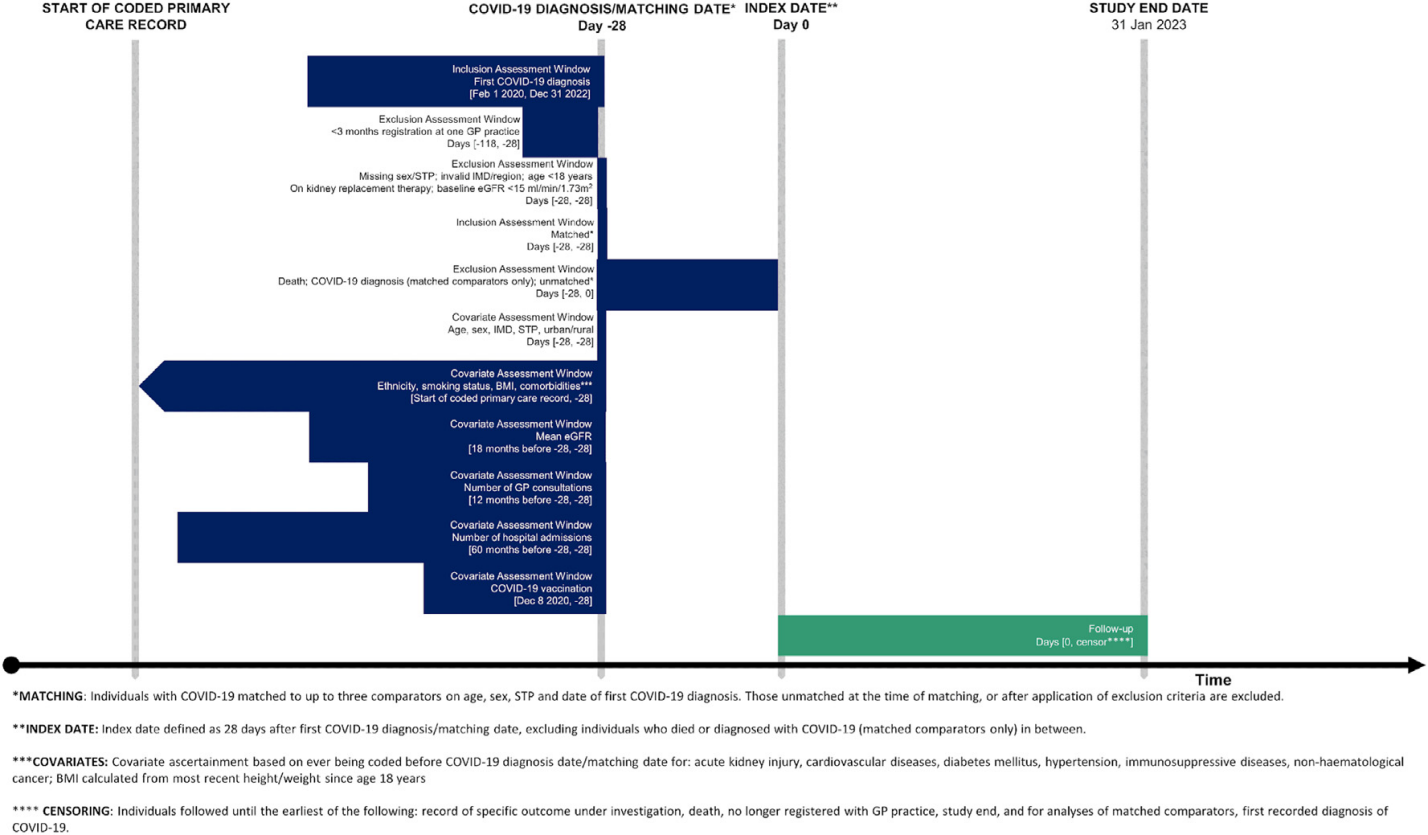
Study design.

We ascertained individuals with COVID-19 based on: a positive test result from SGSS, primary care morbidity coding, or hospital coding with diagnosis date as the first recording of any of these between 1 February 2020 and 31 December 2022 (https://github.com/opensafely/post-covid-kidney-outcomes/tree/main/codelists). We followed individuals from 28 days after the COVID-19 diagnosis date (index date) and excluded anyone who died before this. The rationale for this was that the focus of our research question was on longer-term kidney effects, acknowledging that many patients who required dialysis during COVID-19 hospitalisation died.^1^^,^^2^^,^^4^ Study design decisions, potential biases and mitigations of bias are outlined further in Appendix S2. We ended follow-up at the earliest of: date of GP deregistration, date of death, or end of study (31 January 2023) (Fig. 1). We defined COVID-19 exposure status as: 1) any COVID-19 (i.e., non-hospitalised and hospitalised COVID-19 combined); 2) non-hospitalised COVID-19; and 3) hospitalised COVID-19. People were considered to have been hospitalised with COVID-19 if there was a hospital admission within 28 days of the first record of COVID-19.

We matched individuals with COVID-19 without replacement (i.e., matched comparators could not be reused by subsequent COVID-19 cases), by age, sex and STP with up to three comparators drawn from the general population without any prior diagnosis of COVID-19 in their health record or pre-existing kidney failure before the matching date with their COVID-19 cohort comparator. In line with the COVID-19 cohort, we set the comparator index date as 28 days after this date and excluded anyone who was diagnosed with COVID-19, died, or was deregistered from their primary care practice within those 28 days, and subsequently censored individuals on the earliest of any of these events, or at the end of follow-up on 31 January 2023.

### Outcomes

#### Primary outcome

Our primary outcome was time to kidney failure, defined as a new primary or secondary care morbidity code record for dialysis or kidney transplant (https://github.com/opensafely/post-covid-kidney-outcomes/tree/main/codelists), or eGFR <15 ml/min/1.73 m^2^. Individuals whose first record for dialysis was within 28 days after their first COVID-19 diagnosis date/matching date, were counted as reaching the outcome on Day 1 of study follow-up. The rationale for this was to ensure that important information on the impact of severe COVID-19-AKI was not lost, and also so that ongoing kidney failure was not underestimated due to disproportionate non-recording of further dialysis for patients who died early in follow-up (Appendix S2).

#### Secondary outcomes

Our secondary outcomes were time to 50% reduction in eGFR and all-cause death.

We defined 50% reduction in eGFR as a composite of kidney failure (our primary outcome) or a halving of eGFR compared to baseline.^12^ Baseline eGFR (calculated using the CKD-EPI equation from serum creatinine in primary care test records only, excluding adjustment for ethnicity)^13^^,^^14^ was the mean eGFR in the 18 months preceding COVID-19 diagnosis date/matching date.^13^^,^^14^ An individual met the outcome definition of a first occurrence of a halving in eGFR on the 15th day of the month in which this occurred based on the mean eGFR within that month.

We included all-cause death as an outcome to help contextualise our kidney outcomes given the importance of competing mortality.^2^^,^^15^^,^^16^ We defined death based on recording in primary care.^17^ We did not use Office for National Statistics (ONS) death registration data for consistency, since ONS data are only available within OpenSAFELY from 2019 onwards and therefore could not be used for our pre-pandemic comparator. We did not investigate death due to CKD due to potential misclassification.

### Covariates

Baseline covariates included age, sex, deprivation, ethnicity, region of England, STP, rural or urban, body mass index (BMI), smoking status, baseline eGFR,^18^^,^^19^ previous AKI, cardiovascular diseases, diabetes mellitus, hypertension, immunosuppressed, non-haematological cancer, GP interactions in the year before COVID-19 diagnosis/matching date, hospital admissions in the preceding five years, COVID-19 vaccination status, and COVID-19 wave. Ethnicity was extracted using primary care coding and categorised into white, South Asian, black, mixed, other ethnicities, or missing.^20^ Covariate assessment windows are presented in Fig. 1, and details of definitions/components and categories are outlined in Appendix S3.

### Statistical analysis

#### Main analyses

We initially described sociodemographic and clinical characteristics for people with COVID-19 and their matched comparators.

We used cause-specific Cox regression, stratified by matched set, to estimate adjusted hazard ratios (HR) and 95% confidence intervals (CI) for each outcome after COVID-19 with time from start of follow-up as the underlying timescale (i.e., from 28 days after COVID-19 and the corresponding index date for matched comparators). In separate analyses, we compared with matched comparators: 1) all COVID-19 cases, 2) non-hospitalised cases, and 3) hospitalised cases. We implicitly adjusted for matching factors (age, sex, STP, date) at cohort entry through stratification by matched set. We explicitly adjusted for potential confounders (ethnicity, IMD, rural/urban, BMI, smoking, baseline eGFR (with “no baseline eGFR measurement” treated as a categorical variable), cardiovascular disease, diabetes mellitus, hypertension, immunosuppressed, non-haematological cancer, previous AKI, hospital admissions within the preceding five years, GP consultations within the previous year and COVID-19 vaccination status). We accounted for clustering by primary care practice using robust standard errors. We performed a complete case analysis and so excluded anyone with missing ethnicity, BMI or smoking data, and therefore any matched set in which there was no longer a COVID-19 case and at least one matched comparator. We estimated cause-specific HRs overall and by time period after index date: 0–29 days, 30–89 days, 90–179 days and 180+ days. We estimated cause-specific HRs for outcomes compared to the matched comparators, for COVID-19 overall and by hospitalisation status. We tested the proportional hazards assumption using Schoenfeld residuals.

To estimate period-specific adjusted rate differences, we subtracted crude rates in the COVID-19 group divided by the corresponding hazard ratio from the crude rate.^21^ To obtain 95% confidence intervals, we repeated this using lower and upper bound 95% confidence intervals for each hazard ratio.

#### Sensitivity analyses

##### Historical cohort analysis

To account for misclassification of unrecorded COVID-19, we replicated our main analyses comparing individuals with COVID-19 with a matched historical cohort. We selected our matched historical cohort from the primary care population using the same criteria as our main analysis, but with a matching date three years before the COVID-19 diagnosis date. We set the historical comparator index date as 28 days after the matching date and excluded anyone who died, or was deregistered from their primary care practice within those 28 days and censored individuals after this date on the date of deregistration from their GP, date of death, or on 31 January 2020.

##### Additional sensitivity analyses

To explore the robustness of our results to assumptions made in exposure, outcome and covariate definitions, we also replicated our main analyses in a series of additional sensitivity analyses. These have been described and justified in Table S1.

#### Secondary analyses

##### Effect modification

We investigated whether the effect of COVID-19 (overall, non-hospitalised and hospitalised) on time to kidney failure, 50% reduction in eGFR, and death, was modified by age, sex, ethnicity, diabetes, baseline eGFR, COVID-19 vaccination status, and COVID-19 wave. We adjusted for ethnicity, deprivation, rural or urban, BMI, smoking status, baseline eGFR, previous AKI, cardiovascular diseases, diabetes mellitus, hypertension, immunosuppressed, non-haematological cancer, general practice consultations in the previous year, hospital admissions in the previous five years and COVID-19 vaccination status. We replicated this (for age, sex, ethnicity, diabetes and baseline eGFR) in comparison with our matched historical cohort.

##### Severity of COVID-19 hospitalisation

To determine the risk of kidney failure associated with varying COVID-19 severity, we further categorised COVID-19 status as: i) hospitalised with or without admission to the intensive care unit (ICU); ii) hospitalised with or without AKI; and iii) non-hospitalised COVID-19.

##### Hospitalised COVID-19 compared to pre-pandemic hospitalised pneumonia

To explore whether outcomes after COVID-19 hospitalisation were different from other hospitalised respiratory tract infections episodes, we undertook an additional unmatched analysis comparing individuals with COVID-19 hospitalisation (1 February 2020 to 31 December 2022) to individuals hospitalised with pneumonia (in any coded position) before the pandemic (1 February 2017 to 31 December 2019). We chose to compare with pre-pandemic illness to avoid misclassification of COVID-19 admissions and because of pandemic impacts on other health care. We set the index date of follow-up for kidney outcomes 28 days after the date of COVID-19 or pneumonia hospital admission and excluded anyone who died before this. We censored individuals on the earliest of: date of deregistration from their GP, date of death, or administratively for COVID-19 on 31 January 2023, and for pneumonia on 31 January 2020.

We obtained overall and period-specific HRs for each outcome using Cox models, adjusted for age (parameterised as a four-knot restricted cubic spline), sex, ethnicity, IMD, rural/urban, BMI, smoking, baseline eGFR, cardiovascular disease, diabetes mellitus, hypertension, immunosuppressed, non-haematological cancer, previous AKI, hospital admissions within the preceding five years, GP consultations within the previous year, and calendar month. We accounted for clustering by practice using robust standard errors.

### Software and reproducibility

We used Python 3.9.7 and Stata 16.1 for data management and analysis, and R for data visualisation. Code for data management and analysis, as well as codelists, protocol, and deviations from protocol, are archived online (https://github.com/opensafely/post-covid-kidney-outcomes). Data management and analysis was executed on OpenSAFELY without viewing patient data. Detailed pseudonymised data is potentially re-identifiable and therefore not shared.

### Patient and public involvement

This study has been co-authored with patient and public involvement (PPI) partners who are members of the study steering committee and have contributed throughout the research cycle (identifying and prioritising, designing and managing, and disseminating) (Appendix S4). We received additional contributions from wider groups during the identifying and prioritising, and dissemination stage. PPI has also been a focus of the wider OpenSAFELY project (Appendix S5).

### Ethics approval

This study was approved by the Health Research Authority (REC reference 20/LO/0651) and by the London School of Hygiene & Tropical Medicine's Ethics Board (reference 21863).

### Role of funding source

The funders had no role in study design; in the collection, analysis, and interpretation of data; in the writing of this report; and in the decision to submit this paper for publication.

## Results

### Cohort characteristics

**Fig. 2:**
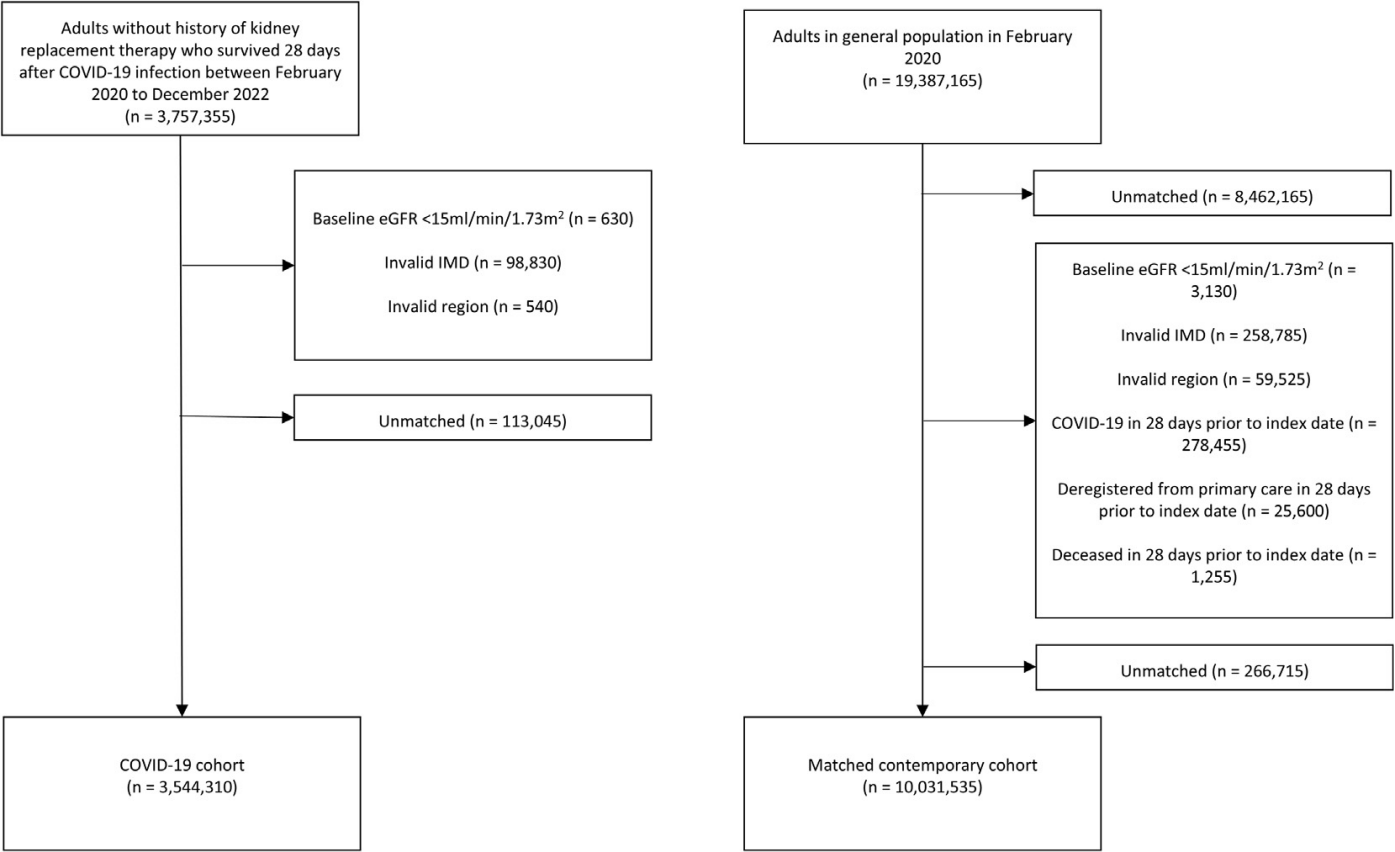
Study population flowchart; adults aged 18+ years, counts rounded to nearest 5. eGFR = estimated glomerular filtration rate, IMD = index of multiple deprivation. Initial extractions from OpenSAFELY platform excluded individuals without valid age, sex, region and IMD.

We included 3,544,310 individuals after COVID-19, matched to 10,031,535 comparators without previously recorded COVID-19 (Fig. 2). Individuals with and without COVID-19 had similar demographic and socioeconomic characteristics, although there was more previous healthcare use amongst individuals in the COVID-19 cohort (Table 1). Median follow-up was 446 days (interquartile range [IQR] 370–700) for the COVID-19 cohort, and 410 days (IQR 361–567) for matched comparators. Amongst those with COVID-19, 243,845 (6.9%) were hospitalised. Those hospitalised with COVID-19 were older, had lower baseline kidney function and more comorbidities that matched comparators (Table S2). After excluding individuals with missing data for ethnicity, BMI or smoking status for complete case analysis, 2,516,030 individuals remained in the COVID-19 cohort with 5,649,105 in the matched cohort (Table S3).

**Table 1:** Baseline characteristics for COVID-19 cohort and an age-, sex-, and sustainability and transformation partnership region-matched cohort.

	COVID-19 cohort	Matched cohort
Number of individuals	3,544,310	10,031,535
Follow-up (days), median (IQR)	446 (370–700)	410 (361–567)
Age (years), median (IQR)	44 (32–57)	44 (32–57)
Sex, n (%)		
Female	1,886,340 (53.2)	5,306,755 (52.9)
Male	1,657,970 (46.8)	4,724,780 (47.1)
Index of multiple deprivation, n (%)		
1 Most deprived	726,765 (20.5)	2,104,640 (21.0)
2	718,950 (20.3)	2,004,605 (20.0)
3	746,720 (21.1)	2,104,315 (21.0)
4	702,980 (19.8)	1,981,500 (19.8)
5 Least deprived	648,890 (18.3)	1,836,475 (18.3)
Ethnicity, n (%)		
White	2,568,170 (72.5)	7,085,150 (70.6)
South Asian	241,010 (6.8)	680,615 (6.8)
Black	74,625 (2.1)	233,515 (2.3)
Mixed	41,485 (1.2)	122,310 (1.2)
Other	50,720 (1.4)	237,785 (2.4)
Missing	568,300 (16.0)	1,672,165 (16.7)
Region, n (%)		
East Midlands	648,825 (18.3)	1,844,310 (18.4)
East	792,995 (22.4)	2,240,430 (22.3)
London	217,915 (6.1)	618,245 (6.2)
North East	193,870 (5.5)	552,690 (5.5)
North West	352,505 (9.9)	995,750 (9.9)
South East	209,785 (5.9)	595,370 (5.9)
South West	430,780 (12.2)	1,224,560 (12.2)
West Midlands	156,475 (4.4)	448,735 (4.5)
Yorkshire and The Humber	541,155 (15.3)	1,511,450 (15.1)
Urban/rural, n (%)		
Urban	2,892,590 (81.6)	8,043,555 (80.2)
Rural	651,715 (18.4)	1,987,985 (19.8)
Body mass index, n (%)		
<18.5 kg/m^2^	58,510 (1.7)	205,770 (2.1)
18.5–24.9 kg/m^2^	1,039,705 (29.3)	3,155,620 (31.5)
25.0–29.9 kg/m^2^	1,023,430 (28.9)	2,750,620 (27.4)
30.0–34.9 kg/m^2^	541,325 (15.3)	1,365,695 (13.6)
35.0–39.9 kg/m^2^	224,720 (6.3)	548,850 (5.5)
≥40.0 kg/m^2^	120,670 (3.4)	294,820 (2.9)
Missing	535,945 (15.1)	1,710,165 (17.0)
Smoking, n (%)		
Non-smoker	1,689,090 (47.7)	4,692,210 (46.8)
Current/former smoker	1,736,760 (49.0)	4,883,770 (48.7)
Missing	118,455 (3.3)	455,555 (4.5)
Comorbidity before index date		
Baseline eGFR (ml/min/1.73 m^2^), median (IQR)	90.3 (75.9–103.1)	89.6 (75.3–102.6)
Previous acute kidney injury, n (%)	69,805 (2.0)	116,575 (1.2)
Cardiovascular diseases, n (%)	299,865 (8.5)	745,695 (7.4)
Diabetes mellitus, n (%)	384,975 (10.9)	976,655 (9.7)
Hypertension, n (%)	656,205 (18.5)	1,748,430 (17.4)
Immunosuppressive diseases, n (%)	75,645 (2.1)	179,915 (1.8)
Non-haematological cancer, n (%)	198,035 (5.6)	517,520 (5.2)
GP consultations previous year, median (IQR)	4 (1–9)	3 (0–8)
Hospital admissions previous 5 years, n (%)		
0	2,010,875 (56.7)	6,220,305 (62.0)
1	664,035 (18.7)	1,736,460 (17.3)
>1	869,400 (24.5)	2,074,770 (20.7)
COVID-19 vaccination status, n (%)		
Unvaccinated	1,426,045 (40.2)	4,470,605 (44.6)
1 vaccine dose	237,525 (6.7)	605,545 (6.0)
2 vaccine doses	1,207,650 (34.1)	2,989,875 (29.8)
3 vaccine doses	633,595 (17.9)	1,849,475 (18.4)
4 vaccine doses	39,495 (1.1)	116,040 (1.2)
COVID-19 wave, n (%)		
February 2020–August 2020 (wild-type)	109,590 (3.1)	320,440 (3.2)
September 2020–June 2021 (Alpha variant)	1,064,495 (30.0)	3,058,780 (30.5)
July 2021–November 2021 (Delta variant)	1,057,055 (29.8)	3,010,410 (30.0)
December 2021–December 2022 (Omicron variants)	1,313,170 (37.1)	3,641,900 (36.3)

Counts rounded to nearest five to preserve anonymity. eGFR = estimated glomerular filtration rate, GP = general practice, IQR = interquartile range.

### Main analyses

#### Kidney failure

**Fig. 3:**
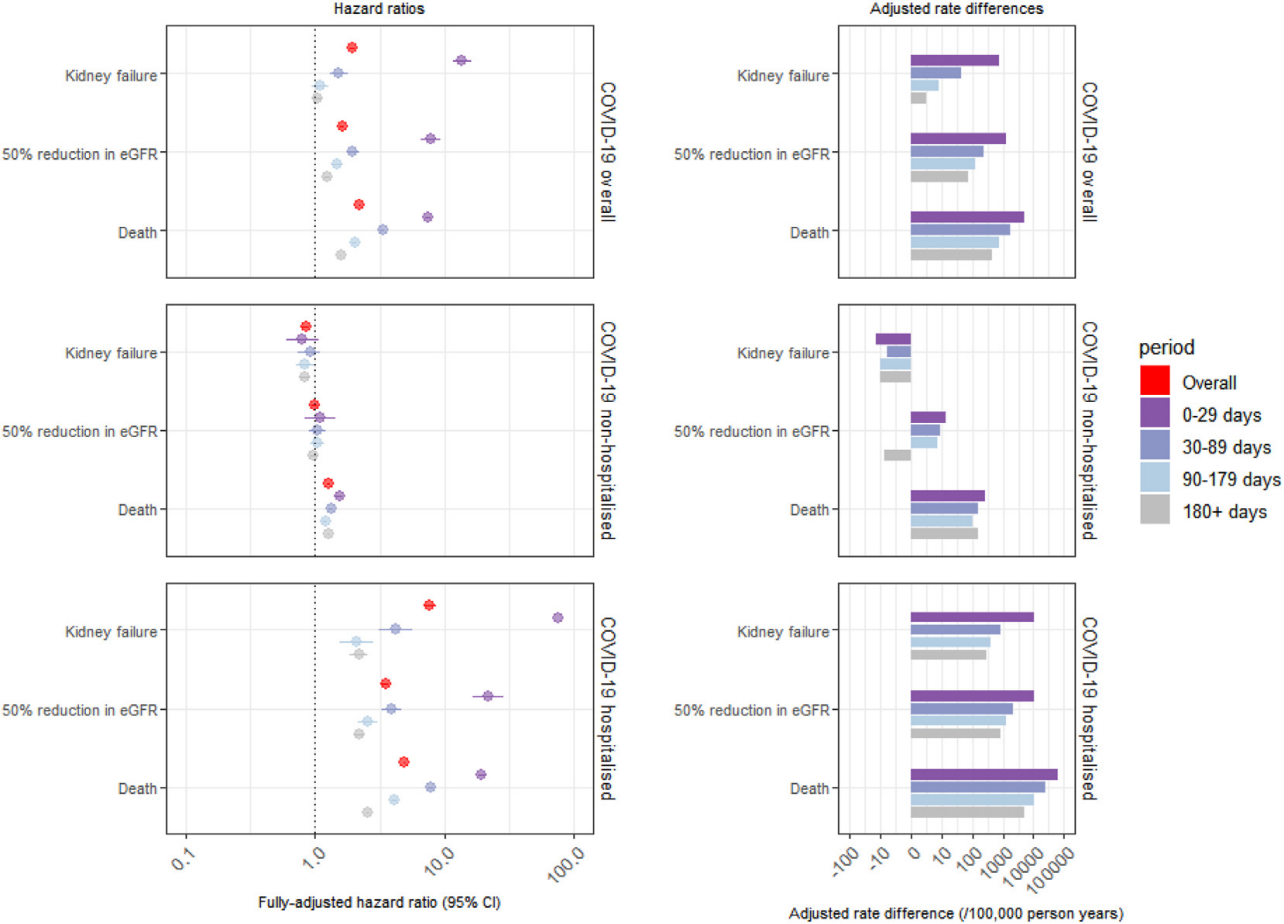
Fully-adjusted hazard ratio and adjusted rate difference estimates for kidney failure (i.e., incident dialysis, kidney transplantation or estimated glomerular filtration rate (eGFR) < 15 ml/min/1.73 m^2^), 50% reduction in eGFR, and death after COVID-19 compared to an age-, sex- and sustainability and transformation partnership region-matched cohort, overall and by COVID-19 hospitalisation status by specific follow-up periods (in days since index date, i.e., 28 days after first COVID-19 infection record) (Table S4). Models adjusted for ethnicity, deprivation, rural or urban, body mass index, smoking status, baseline eGFR (with “no baseline eGFR measurement” treated as a categorical variable), previous acute kidney injury, cardiovascular diseases, diabetes mellitus, hypertension, immunosuppressive diseases, non-haematological cancer, general practice consultations in the previous year, hospital admissions in the previous five years and COVID-19 vaccination status. Fully-adjusted hazard ratio and adjusted rate difference on log scale. CI = confidence interval.

Overall, we saw an increased hazard (subsequently ‘risk’) of kidney failure after COVID-19 compared to matched comparators (HR 1.93 [95% CI 1.84–2.03]) (Fig. 3; Table S4). There was evidence of non-proportional hazards with kidney failure decreasing over time after infection, with greatest risk in the first 29 days after index date (HR 13.65 [95% CI 11.59–16.07]) and no evidence of an increase in kidney failure from 90 days after index date (HR 1.10 [95% CI 0.95–1.27]). The adjusted rate difference at 0–29 days was 756 per 100,000 person years (95% CI 745–765), and at 30–89 days was 43 (95% CI 30–55).

We observed a lower risk of kidney failure in those with COVID-19 who were not hospitalised compared to the matched cohort (HR 0.85 [95% CI 0.79–0.90]). However, there was strong evidence that COVID-19 requiring hospitalisation was associated with kidney failure (HR 7.74 [95% CI 7.00–8.56]). For people who were hospitalised, HR estimates were increased across all time periods including beyond 180 days (HR 2.20 (95% CI 1.87–2.58); adjusted rate difference 292 per 100,000 person years [95% CI 249–328]), with the greatest risk in the first 29 days (HR 75.9 [95% CI 52.7–109.4]).

#### 50% eGFR reduction and death

Overall, there was an increased risk in both 50% eGFR reduction and death after COVID-19 compared to the matched cohort: 50% reduction in eGFR, HR 1.62 (95% CI 1.56–1.68), and death, HR 2.22 (95% 2.19–2.25) (Fig. 3; Table S4). Of 7070 individuals who developed 50% reduction in eGFR after COVID-19, 3805 (53.8%) of these were due to incident kidney failure. Of 57,530 individuals with COVID-19 who died, 1550 (2.7%) had reached kidney failure by the time of death, including 400 (0.7%) with eGFR <15 ml/min/1.72 m^2^ not on KRT. Increased HRs for 50% eGFR reduction and death were maintained beyond 180 days.

Amongst individuals with COVID-19 who were not hospitalised, we saw no increase in risk of 50% reduction of eGFR (HR 0.99 [95% CI 0.95–1.04]) but there was a consistent increased risk of death (HR 1.27 [95% CI 1.25–1.29]). Both 50% eGFR reduction (HR 3.49 [95% CI 3.25–3.75]) and death (HR 4.93 [95% CI 4.83–5.04]) were consistently increased in those hospitalised with COVID-19.

#### Proportional hazards

While there was evidence of non-proportionality (Table S5) related to the change in risk after infection, we report weighted-average HR estimates, which maintained internal validity over the course of study follow-up and facilitated meaningful comparisons for sensitivity and secondary analyses. To directly address the non-proportionality over time we also report period-specific HRs, which offer a more nuanced understanding of temporal risk dynamics.^22^

### Sensitivity analyses

#### Historical cohort analysis

We saw a similar distribution of demographic and socioeconomic characteristics between the COVID-19 cohort and matched historical cohort with more previous healthcare use amongst individuals with COVID-19 (Table S6). When compared to the matched historical cohort, there were similar patterns of time to kidney failure, 50% reduction in eGFR and death after COVID-19, overall and among hospitalised patients. However, no increased risk of death was observed in those who were not hospitalised (Figure S2; Table S7).

#### Additional sensitivity analyses

The risk of kidney failure after COVID-19 persisted after restricting to coded KRT outcomes alone, after multiple imputation for missing ethnicity data, and after restriction of COVID-19 cases to those diagnosed up to the end of universal access to testing in March 2022. There was a decrease in the magnitude of overall risk of kidney failure (HR 1.10 [95% CI 1.04–1.17]) and 50% reduction in eGFR (HR 1.36 [95% CI 1.31–1.42]) after excluding individuals who required dialysis within 28 days of COVID-19 diagnosis (i.e. before the index date), as well as a decrease amongst those hospitalised with COVID-19, HR 2.34 (95% CI 2.16–2.74) and 2.46 (95% CI 2.28–2.64) for each outcome respectively. There was also a decrease in kidney failure (HR 1.22 [95% CI 1.15–1.29]) and 50% reduction in eGFR (HR 1.29 [95% CI 1.24–1.34]) when only including codes for KRT from 28 days after COVID-19 (i.e., after the index date). For those hospitalised with COVID-19, HR estimates decreased to 3.06 (95% CI 2.73–3.43) for kidney failure and 2.40 (95% CI 2.24–2.58) for 50% reduction in eGFR (Figure S3; Table S1).

### Secondary analyses

#### Effect modification

**Fig. 4:**
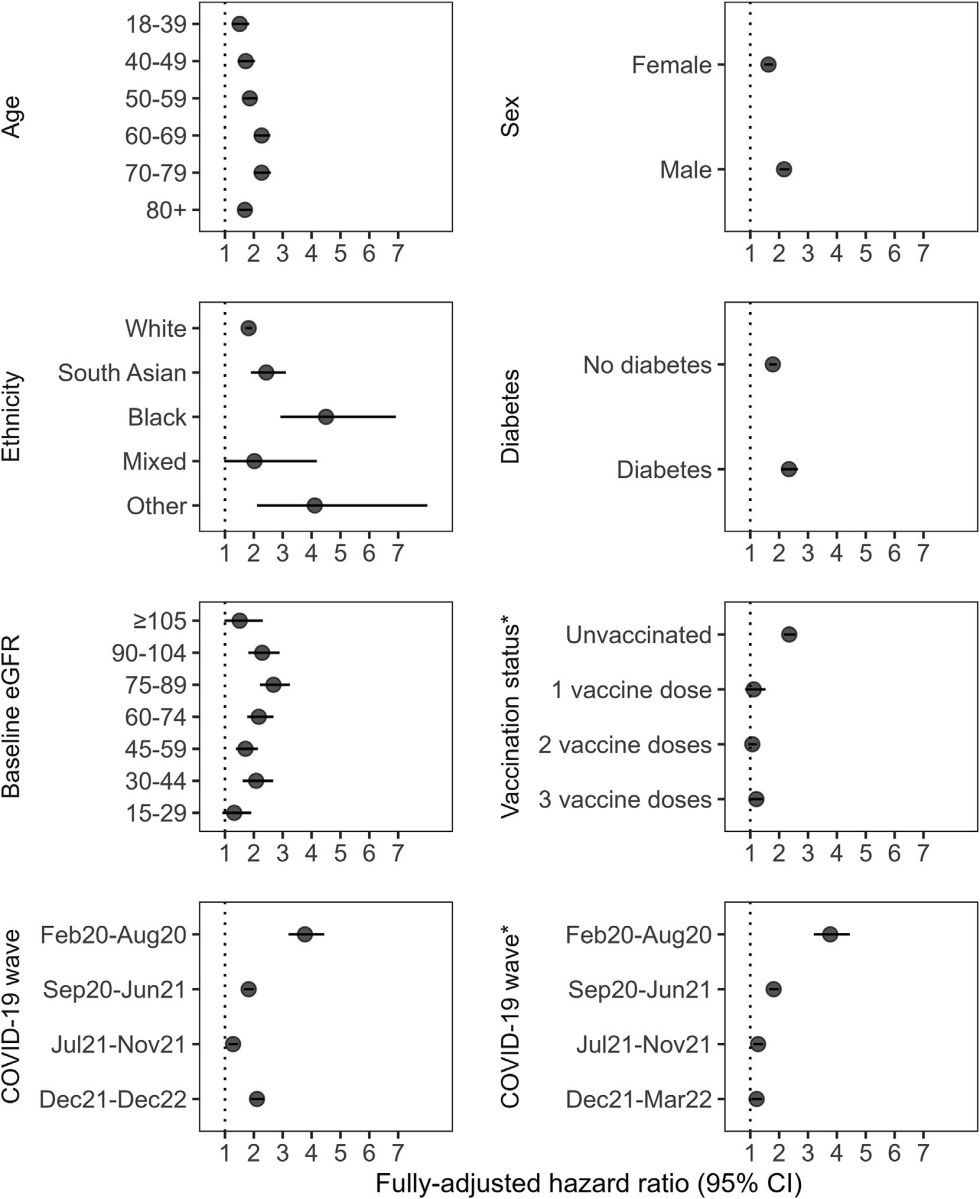
Fully-adjusted hazard ratio estimates for kidney failure (i.e., incident dialysis, kidney transplantation or estimated glomerular filtration rate (eGFR) < 15 ml/min/1.73 m^2^), stratified by potential effect modifiers, for COVID-19 compared to an age-, sex- and sustainability and transformation partnership region-matched cohort (Table S8). Models adjusted for ethnicity, deprivation, rural or urban, body mass index, smoking status, baseline eGFR (with “no baseline eGFR measurement” treated as a categorical variable), previous acute kidney injury, cardiovascular diseases, diabetes mellitus, hypertension, immunosuppressive diseases, non-haematological cancer, general practice consultations in the previous year, hospital admissions in the previous five years and COVID-19 vaccination status. Age in years. Baseline eGFR in ml/min/1.73 m^2^. CI = confidence interval. ∗COVID-19 vaccination status and COVID-19 wave restricted to cases up to March 2022 (i.e., the end of universal access to COVID-19 testing).

We found evidence that the overall association between recorded COVID-19 and time to kidney failure was modified by age, sex, ethnicity, diabetes, baseline eGFR, vaccination status and wave (Fig. 4; Table S8; Figure S4; Table S9), driven by substantial differences in the hazard ratios among hospitalised people (Figure S5; Table S10; Figure S6; Table S11), most markedly for ethnicity. The HR for the primary outcome was 4.50 (95% CI 2.92–6.92) for black ethnic groups compared to 2.42 (95% CI 1.90–3.11) for South Asian ethnic groups and 1.82 (95% CI 1.71–1.94) for white ethnic groups. The HR was greater for men (HR 2.17 [95% CI 2.01–2.35]) than for women (HR 1.63 [95% CI 1.49–1.79]). HRs were highest among unvaccinated people and among those who had received four vaccine doses (i.e., individuals offered multiple doses due to their high risk of severe outcomes). A strong association between kidney failure and COVID-19 hospitalisation (where differential ascertainment of infection was unlikely to be a substantial issue, in contrast to non-hospitalised individuals), persisted up to December 2022 (HR 8.36 [95% CI 6.81–10.27]). There was a progressive weakening of the association between COVID-19 and time to 50% reduction in eGFR with each successive wave from HR 2.34 (95% CI 2.08–2.65) for the first wave to 1.19 (95% CI 1.07–1.34) for the period between December 2021 and March 2022 (Figure S7; Table S12). The HRs for all outcomes persisted over successive COVID-19 waves amongst those hospitalised (Figure S7; Table S13).

#### Severity of COVID-19 hospitalisation

Amongst individuals hospitalised with COVID-19, compared to matched comparators, the increased risks of kidney failure were most pronounced after ICU admission (HR 47.38 [95% CI 36.49–61.50]) and separately among those who had coded AKI during admission (HR 28.40 [95% CI 23.63–34.13]). However, risks notably remained elevated even amongst those who were hospitalised but not in ICU (HR 3.19 [95% CI 2.84–3.59]) and without coded AKI (HR 2.53 ([95% CI 2.21–2.90]) (Figure S8; Table S14).

#### Hospitalised COVID-19 compared to hospitalised pneumonia

Compared to individuals with hospitalised pneumonia pre-pandemic (Figure S9), those who were hospitalised with COVID-19 were younger, more likely to be from a South Asian or black ethnic group, more likely to be obese or a non-smoker, and had fewer comorbidities or history of previous healthcare utilisation (Table S15).

In fully-adjusted Cox models, risk of all outcomes were lower overall after hospitalised COVID-19 compared to pre-pandemic hospitalised pneumonia (Figure S10; Table S16).

## Discussion

In our study of over 13 million adults in England, we found an overall increase in long-term kidney outcomes in people with COVID-19 compared to individuals without a record of COVID-19. There was a persistent increased risk when the need for dialysis around the time of infection was excluded, though of lower magnitude. Amongst people not hospitalised for COVID-19 there was no increase in kidney outcomes. In hospitalised patients, outcomes were highest in those who required ICU and in those with concurrent AKI, though risks remained notably elevated amongst hospitalised patients without AKI and in those receiving only ward level care too. While the overall risks of kidney outcomes decreased over the pandemic, they did not disappear: amongst those who were hospitalised with COVID-19 during the Omicron wave, kidney outcomes remained substantially increased despite high vaccination coverage, dominance of less virulent variants, and the availability of anti-COVID-19 therapies. There was an increase in the risk of kidney failure in men and people from black ethnic groups compared to other groups. Vaccination was associated with a reduction in risk.

A strength of our analysis is that we used linked primary and secondary care records with extensive coverage in a setting of universal healthcare, providing statistical power and robust capture of kidney failure, a relatively rare outcome. While coverage of London was lower than other parts of England, OpenSAFELY-TPP is broadly representative of the population in terms of ethnicity.^10^ We determined both relative and absolute risk to understand health service impacts. In addition, our study period continued through the pandemic until an era where the majority of the population was vaccinated, although we cannot be certain that patterns were similar for patients who developed COVID-19 after December 2022. In the UK, universal community access to COVID-19 testing was available in some form between September 2020 and March 2022,^23^ limiting the impact of selective testing which would have led to differential misclassification and underestimation outcomes. The use of highly detailed primary care records enabled us to adjust for important potential confounding factors and we used almost three years of incident COVID-19 cases in our analysis. We importantly undertook a sensitivity analysis replicating our main analysis using a matched historical cohort from before the COVID-19 pandemic to be able to address potential misclassification within the control group, with broadly similar findings.

As with all observational analyses of this topic there are sources of confounding and bias which we have sought to make explicit and address where possible using this detailed data.^24^ Individuals hospitalised with COVID-19 were likely to have higher baseline risk of kidney failure than those who were not hospitalised. This depletion of higher risk individuals from the non-hospitalised group contributed to the lower observed risk of kidney outcomes compared to non-infected comparators, with likely residual confounding due to factors not adequately captured in health records (e.g., the severity of comorbidities such as diabetes and cardiovascular diseases). Early depletion of those most susceptible to kidney failure from the non-infected cohort due to kidney failure or death from COVID-19 may have contributed to the persistent risk of kidney failure among hospitalised COVID-19 patients throughout the pandemic (Appendix S6). Depletion of those at greatest risk of the outcome likely also affected the comparison to pre-pandemic pneumonia,^15^^,^^16^ where patients surviving a month after COVID-19 were younger with better baseline kidney function and fewer comorbidities, and thus inherently with lower risk of subsequent kidney failure (Appendix S6). In relation to the secondary outcomes, determining 50% reduction in eGFR is dependent on having regular blood tests, more likely among patients with chronic health conditions who may also be more likely to test for and record COVID-19, resulting in collider bias (Appendix S6). Some admissions which we have categorised as hospitalised infections may be due to other causes in which COVID-19 occurred incidentally; while the cause of these admissions may have been related to degree of kidney failure risk (in both directions), our results will have captured the full risk associated with COVID-19 rather than specific to patients admitted primarily for COVID-19. While the summary result for the risk of kidney failure over the whole period was driven by the substantial risk during the first COVID-19 wave, the effects in hospitalised patients, even within individual waves, were consistently raised throughout the pandemic and are a more reliable measure of the ongoing impact. Inevitably, there will be sources of residual confounding as there were important covariates we were unable to adjust for such as occupation, and we did not adjust for type of vaccination.^25^ Lastly, while we undertook a complete case analysis with regard to ethnicity, BMI and smoking data, results were similar after using multiple imputation for missing ethnicity.

Most previous studies addressing risks of kidney related outcomes after COVID-19 have been among hospitalised cohorts, long-COVID clinics or volunteers.^26^, ^27^, ^28^, ^29^, ^30^, ^31^, ^32^ The only other large-scale study was conducted among predominantly male and older US military veterans up to March 2021.^5^ However, unlike the US study, we found no evidence of an increase in kidney outcomes amongst those not hospitalised for COVID-19, likely because our analysis included a healthier population with greater access to COVID-19 testing across a longer period.

It remains unknown whether kidney outcomes after COVID-19 hospitalisation are causally specific to COVID-19 or whether they are a consequence of severe illness. Kidney organoids infected with SARS-CoV-2 (the virus that causes COVID-19) have signs of injury and upregulated various pro-fibrotic signalling pathways, however, direct evidence implicating the virus in AKI and CKD pathogenesis is lacking.^33^^,^^34^ We have previously shown an accelerated decline in eGFR amongst survivors of hospitalised COVID-19 compared to pre-pandemic hospitalised pneumonia in Sweden,^9^ while in this study we found decreased kidney outcomes after COVID-19 compared to pneumonia. These differences highlight the challenges in comparing outcomes in survivors of both presentations over time (Appendix S6). Due to the potential severity of COVID-19, a proportion of admissions were previously relatively healthy individuals with a low baseline risk of kidney outcomes. Conversely, the population admitted to hospital with pneumonia prior to the pandemic were older, frailer people with comorbidities and a high baseline risk of kidney outcomes. During the pandemic, people with these characteristics would have had high early mortality and been less likely to survive to develop kidney outcomes, thus biasing the risk of kidney outcomes lower after COVID-19 compared to pneumonia. Bias due to this differential mortality is likely more problematic in England, where in-hospital COVID-19 mortality was around 30% in the early stages of the pandemic,^35^ than in Sweden where the 60-day mortality was 17%.^36^

We found a substantial burden on acute dialysis around the time of COVID-19, reflecting the difficult experiences of healthcare workers.^1^^,^^4^ Our quantification of this demand can help inform resource requirements for future pandemic planning. The persistent increased risk of outcomes amongst those hospitalised with COVID-19, even during the latter stages of the pandemic, affirms the importance of ongoing public health measures such as vaccination to minimise severe disease, especially amongst those who are clinically vulnerable. Based on our previous finding of a 5.4% annual decline in eGFR in hospitalised survivors of COVID-19,^9^ we recommend that this group undergo eGFR and albuminuria screening in primary care, and are thereafter monitored at least annually (or more frequently if eGFR <45 ml/min/1.73 m^2^ or if there is albuminuria). We recommend that management of CKD is in accordance with international or local guidelines.^18^ However, people who were not hospitalised can be reassured that there was no evidence of increased risk of longer-term kidney outcomes after COVID-19.

An important finding from our secondary analysis was the marked ethnic inequalities in kidney outcomes after COVID-19, with people from black ethnic groups having a twofold higher risk of kidney failure compared to white and South Asian individuals, even after adjustment for baseline kidney function, other health conditions and vaccination status. Studies from the UK and USA from early stages of the pandemic found an increase in COVID-19 related AKI in black ethnic groups.^3^^,^^4^^,^^37^ Understanding the cause of these unequal outcomes requires further investigation as well as prioritisation in future pandemic considerations. Plausible explanations include residual confounding by socioeconomic factors (e.g., occupational risks), underdiagnosed or suboptimally managed pre-existing comorbidities (e.g., CKD, hypertension), delayed management of COVID-19, and ancestral factors (e.g., Apoprotein L1 risk variants).^37^^,^^38^

Our finding of increased long-term kidney outcomes after COVID-19 hospitalisation in a setting with universal healthcare, free at the point of delivery, is consistent with studies from Western Europe and North America.^5^^,^^9^ Generalisability of our findings beyond these economies remains unknown. While COVID-19 was associated with a considerable burden of AKI in low- and middle-income countries,^39^ long-term kidney outcomes are unclear but may be contributing to the ongoing impact of the pandemic in resource-constrained settings.

### Conclusion

While the risk of long-term kidney outcomes after COVID-19 improved over the course of the pandemic, we found a substantial increase amongst hospitalised patients which persisted into an era of vaccination and improved management. The degree of increased risk differs between subgroups and most notably between people from different ethnic groups. Our results suggest that interventions to minimise the risk of severe COVID-19 should continue to be optimised among vulnerable groups, and that kidney function should be proactively monitored after discharge.

## Contributors

Conceptualisation: Viyaasan Mahalingasivam, Sandra Jayacodi, Edith Jumbo, Tamanna Miah, Brian Gracey, Kathryn E Mansfield, Laurie Tomlinson.

Data curation: Viyaasan Mahalingasivam, Bang Zheng, Kevin Wing, Edward P K Parker, Krishnan Bhaskaran, John Tazare, Shalini Santhakumaran, Rohini Mathur, Emily Herrett, Amelia Green, Louis Fisher, Helen J Curtis, Alex J Walker, William J Hulme, Elizabeth Williamson.

Formal analysis: Viyaasan Mahalingasivam, Bang Zheng.

Funding acquisition: Viyaasan Mahalingasivam.

Investigation: Viyaasan Mahalingasivam, Bang Zheng, Kevin Wing, Edward P K Parker, Krishnan Bhaskaran, John Tazare, Shalini Santhakumaran, Rohini Mathur, Emily Herrett, Amelia Green, Louis Fisher, Helen J Curtis, Alex J Walker, William J Hulme, Elizabeth Williamson.

Methodology: Viyaasan Mahalingasivam, Bang Zheng, Kevin Wing, Krishnan Bhaskaran, John Tazare, Ian J Douglas, William J Hulme, Elizabeth Williamson, Dorothea Nitsch, Kathryn E Mansfield, Laurie Tomlinson.

Project administration: Viyaasan Mahalingasivam.

Resources: Amir Mehrkar, Sebastian Bacon, Ben Goldacre

Software: Viyaasan Mahalingasivam, Bang Zheng, Kevin Wing, Edward P K Parker, Krishnan Bhaskaran, John Tazare, Shalini Santhakumaran, Rohini Mathur, Emily Herrett, Amelia Green, Louis Fisher, Helen J Curtis, Alex J Walker, William J Hulme, Sebastian Bacon, Elizabeth Williamson.

Supervision: Kevin Wing, Juan Jesús Carrero, Krishnan Bhaskaran, Elizabeth Williamson, Dorothea Nitsch, Kathyrn E Mansfield, Laurie Tomlinson.

Validation: Viyaasan Mahalingasivam, Kevin Wing.

Visualisation: Viyaasan Mahalingasivam, Edward P K Parker.

Writing–original draft: Viyaasan Mahalingasivam.

Writing–review & editing: Viyaasan Mahalingasivam, Bang Zheng, Kevin Wing, Edward P K Parker, Krishnan Bhaskaran, Juan Jesús Carrero, Sandra Jayacodi, Edith Jumbo, Tamanna Miah, Brian Gracey, John Tazare, Shalini Santhakumaran, Ruth E Costello, Emily Herrett, Qing Wen, Thomas Hartney, Ian J Douglas, Brian MacKenna, Amir Mehrkar, Elizabeth Williamson, Dorothea Nitsch, Kathyrn E Mansfield, Laurie Tomlinson.

Access to the underlying identifiable and potentially re-identifiable pseudonymised electronic health record data is tightly governed by various legislative and regulatory frameworks, and restricted by best practice. The data in the NHS England OpenSAFELY COVID-19 service is drawn from General Practice data across England where TPP is the data processor.

TPP developers initiate an automated process to create pseudonymised records in the core OpenSAFELY database, which are copies of key structured data tables in the identifiable records. These pseudonymised records are linked onto key external data resources that have also been pseudonymised via SHA-512 one-way hashing of NHS numbers using a shared salt. University of Oxford, Bennett Institute for Applied Data Science developers and PIs, who hold contracts with NHS England, have access to the OpenSAFELY pseudonymised data tables to develop the OpenSAFELY tools.

These tools in turn enable researchers with OpenSAFELY data access agreements to write and execute code for data management and data analysis without direct access to the underlying raw pseudonymised patient data, and to review the outputs of this code. All code for the full data management pipeline — from raw data to completed results for this analysis — and for the OpenSAFELY platform as a whole is available for review at github. com/OpenSAFELY.

The data management and analysis code for this paper was led by VM and contributed to BZ, KW, KB, JT, RM, EH, AG, LF, AJW and EW.

VM and LT were responsible for the decision to submit for publication.

## Data sharing statement

All data were linked, stored and analysed securely using the OpenSAFELY platform, https://www.opensafely.org, as part of the NHS England OpenSAFELY COVID-19 service. Data include pseudonymised data such as coded diagnoses, medications and physiological parameters. No free text data are included. All code is shared openly for review and re-use under MIT open license [https://github.com/opensafely/post-covid-kidney-outcomes]. Detailed pseudonymised patient data is potentially re-identifiable and therefore not shared.

## Declaration of interests

VM held a Career Development Award from the National Institute for Health and Care Research (NIHR301535). The funders had no role in study design, data collection, data analysis, data interpretation, or the writing of this report. He received a travel grant from the European Renal Association.

EP has received funding from the UKRI/MRC (MR/W021420/1), NIHR (NIHR200929, NIHR206900), and Bill & Melinda Gates Foundation (OPP1210509). He has received funding as a consultant for Tulane University as part of the ‘Safe in Pregnancy and Childhood Study’, supported by the Safety Platform for Emergency vACcines (SPEAC), Task Force for Global Health, Inc.—The Coalition for Epidemic Preparedness Innovations (CEPI). He received reimbursements for providing input on an expert report for the UK COVID-19 Inquiry.

KB is funded by a Wellcome Senior Research Fellowship.

JJC has received funding to Karolinska Institutet from AstraZeneca, Boehringer Ingelheim, MSD, Vifor Pharma and NovoNordisk. He has received honoraria from Fresenius Kabi and Laboratorios Columbia.

RM is supported by Barts Charity (MGU0504).

RC has shares in AstraZeneca unrelated to this work.

EH is funded by the National Institute for Health and Care Research.

ID has received unrelated research grants from GlaxoSmithKline and AstraZeneca. He holds shares in GlaxoSmithKline.

BMacK is employed by NHS England working on medicines policy and clinical lead for primary care medicines data. All past declarations can be seen at https://www.whopaysthisdoctor.org/doctor/491/active.

AM is a senior clincal researcher at the University of Oxford in the Bennett Institute, which is funded by contracts and grants obtained from the Bennett Foundation, Wellcome Trust, NIHR Oxford Biomedical Research Centre, NIHR Applied Research Collaboration Oxford and Thames Valley, Mohn-Westlake Foundation and NHS England. He has represented the RCGP in the health informatics group and the Profession Advisory Group that advises on access to GP Data for Pandemic Planning and Research (GDPPR); the latter is a paid role. AM is a former employee and interim Chief Medical Officer of NHS Digital (now merged into NHS England, having left NHS Digital in January 2020).

SB received consulting fees from Madalena Consulting LLC by direct payment and Respiratory Matters Ltd by direct payment.

BGo has received research funding from the Bennett Foundation, the Laura and John Arnold Foundation, the NHS National Institute for Health Research (NIHR), the NIHR School of Primary Care Research, NHS England, the NIHR Oxford Biomedical Research Centre, the Mohn-Westlake Foundation, NIHR Applied Research Collaboration Oxford and Thames Valley, the Wellcome Trust, the Good Thinking Foundation, Health Data Research UK, the Health Foundation, the World Health Organisation, UKRI MRC, Asthma UK, the British Lung Foundation, and the Longitudinal Health and Wellbeing strand of the National Core Studies programme; he has previously been a Non-Executive Director at NHS Digital; he also receives personal income from speaking and writing for lay audiences on the misuse of science.

DN has funding from NIHR, MRC and the UK health foundation for work unrelated to COVID-19. She is the chair of the DMEC of the NIHR-funded OPTICAL study (unrelated to this work). She is the UKKA Director of Informatics Research.

LT holds an NIHR Professorship (NIHR302405). The funders had no role in study design, data collection, data analysis, data interpretation, or the writing of this report. She has received travel expenses for MHRA expert advisory group meetings. She is an unpaid member of three trial steering committees (NIHR funded).

## Appendix A Supplementary data

Protocol
