# Supplementary material for: Long-term kidney outcomes after COVID-19: a matched cohort study using the OpenSAFELY platform
Source: Lancet Reg Health Eur. 2025 Jun 18;55:101338. doi: 10.1016/j.lanepe.2025.101338 (PMC12426834; doi:10.1016/j.lanepe.2025.101338)
Supplement: Protocol [file mmc1.pdf]

Protocol: Long-term kidney outcomes after SARS-CoV-2 infection in the general population

Viyaasan Mahalingasivam, Kathryn E. Mansfield, Dorothea Nitsch, Juan-Jesús Carrero, Krishnan Bhaskaran, John Tazare, Alex Walker, Elizabeth Williamson, Brian MacKenna, OpenSAFELY collaborative, National Core Studies Team... Laurie A. Tomlinson

Date: April 2022

1. Lay summary

1.1 Background

People who require hospital admission for COVID-19 usually have severe lung infection which means they can struggle to maintain oxygen levels. However, other organs can also be affected, with the kidneys being among the most common. Around a third of people admitted to hospital with COVID-19 develop kidney damage (known as acute kidney injury or AKI); of these, around 10% develop such severe AKI that they require short-term dialysis (treatments to clear out toxins and excess fluid from their bodies). The reasons for this AKI are yet to be definitively understood.

It is not unusual for AKI to complicate any severe illness (such as life-threatening infections or trauma), especially in people who have other medical problems such as diabetes, chronic kidney disease (CKD) or heart failure. We do not know whether AKI related to COVID-19 just reflects this or whether it is because of processes directly related to COVID-19 infection itself.

While AKI is often recoverable, it can sometimes lead to worsening long-term kidney function. Chronic kidney disease is diagnosed when kidney function declines to less than 60% of what it should be. In some cases, chronic kidney disease can lead to permanent kidney failure requiring long-term dialysis (often involving attending a dialysis unit three-times a week for four hours each time), or a kidney transplant. Even without kidney failure, chronic kidney disease is an important risk factor for several other complications including heart disease and death. The complications caused by chronic kidney disease place considerable stress on the NHS and it is important that we know what to expect to be able to ensure that services are planned with sufficient capacity to cope. For example, growing numbers requiring dialysis has a devastating impact on quality of life for those people and will require investment in the workforce and infrastructure. It can also mean more people requiring hospital admissions for heart attacks.

One way of determining what happens to kidney function after COVID-19 is to analyse readily-available health records collected from day-to-day patient care. A recent study of

this sort in US military veterans suggested that people who survived COVID-19 are more likely to develop kidney failure and lose 50% of their kidney function compared to levels before they had COVID-19. We plan to investigate this in greater detail using electronically coded NHS medical records from England using a secure, Trusted Research Environment platform called OpenSAFELY (www.opensafely.org). OpenSAFELY comprises 40% of the general population in England (24 million people).

This study has been funded by the National Institute for Health Research (NIHR). The study was approved by the Health Research Authority and the London School of Hygiene & Tropical Medicine Ethics Board.

1.2 Methods

Our study will include adults not already on dialysis and not already having received a kidney transplant. We will compare people with and without COVID-19 for differences in the rates of the following kidney complications:

1. Newly-diagnosed kidney failure (i.e. new dialysis or kidney transplant, or loss of kidney function down to less than 15% of what it should be),

2. At least 50% loss in kidney function from before they developed COVID-19,

3. Further AKI (suggesting their kidneys are more susceptible to future damage).

We will be able to determine whether outcomes vary based on the severity of COVID-19 (i.e. based on whether people required hospitalisation, intensive care, a ventilator, dialysis or had AKI), based on time (e.g. during peaks, after widespread use of effective anti-COVID-19 drugs such as steroids), and based on their levels of vaccination.

In our statistical analysis, we will account for factors such as age, sex, ethnicity, socioeconomic deprivation, smoking, BMI, and pre-existing medical problems such as diabetes.

1.3 Strengths and limitations

As kidney failure is a relatively rare occurrence in the general population, very large studies are needed to be able to investigate whether or not a group is at increased risk compared to other groups. Because of the size of the population in OpenSAFELY, we are uniquely placed to be able to look for this in the context of COVID-19. Furthermore, we can expect the data to be comprehensive as it comes from GPs and hospitals within a national healthcare system with universal coverage.

As with other studies of this nature, we anticipate challenges associated with real-world data collection. We will be as rigorous as possible to address these limitations by undertaking multiple analyses with slight differences. For example, we will compare people with COVID-19 (including people hospitalised and not hospitalised) to different groups of people without COVID-19:

1. A historical group comprising individuals followed-up from exactly 2 years before the person with COVID-19. The reason for using a historical comparator group is because not everyone with COVID-19 gets diagnosed, especially when access to testing has been limited.

2. A contemporary group comprising individuals followed-up from the same time as the person with COVID-19. The reason for using a contemporary comparator as well is to account for changes over time during the pandemic such as differences in access to healthcare (e.g. likely to have the same access to blood tests).

A further historical analysis will be restricted only to people hospitalised with COVID-19. In this analysis, we will compare this group to people hospitalised with other lung infections from February 2018 onwards up to the start of the pandemic. This will help us understand whether any differences in kidney outcomes are specifically due to COVID-19 or due to having any lung infection severe enough to require hospital admission.

This particular study will not investigate outcomes in kidney transplant recipients. One of the reasons for this is because blood test results in OpenSAFELY come from GP records

rather than hospitals where many transplant patients have their tests done. We do intend to work around this with a different study design.

2. Background

During the COVID-19 pandemic, evidence has accumulated on an association between COVID-19 hospitalisation and short-term kidney outcomes such as AKI. This has led to plausible concern about longer-term kidney complications, although definitive understanding is so far limited.

2.1 COVID-19 and AKI

AKI describes a clinical state of potentially reversible reduction in kidney function. It is diagnosed when there is a ≥50% or ≥26.5 μmol/l rise in serum creatinine (a biomarker of kidney function) on blood tests compared to baseline, or a reduction in urine output to <0.5 ml/kg/hour based on body weight[1]. AKI is not unusual in acute illness (due to hypoperfusion and/or inflammatory processes), especially on a background of comorbidity (e.g., diabetes, CKD, heart failure), and factors including increasing age and socioeconomic deprivation also contribute[2].

Initial reports on the incidence of AKI in COVID-19 from China were low, and this may be because patients had fewer comorbidities and were admitted for less severe COVID-19 than in other settings[3]. A more recent analysis of over 40,000 individuals in the International Severe Acute Respiratory and emerging Infections Consortium World Health Organization Clinical Characterization Protocol UK (ISARIC-WHO CCP-UK) study, a large prospective cohort comprising patients admitted with COVID-19 to over 250 hospitals across the UK up to early December 2020, found that 31.5% of patients developed AKI. As pre-admission serum creatinine data was unavailable, AKI could only be determined in patients who had at least 2 inpatient measurements and so it is possible people more likely to develop AKI were more likely to have repeated tests; conversely, patients with stable

elevated serum creatinine from the time off admission may have been misclassified as not having AKI resulting in underestimation. This study also found that 3% of over 80,000 inpatients required kidney replacement therapy (KRT), the treatment for severe AKI[4]. Pre-existing CKD was strongly associated both AKI and KRT, as were physiological markers of more severe COVID-19 at hospital admission (i.e. tachypnoea and hypoxia). The variability in estimates of the incidence of AKI may reflect differences in national and local criteria for hospital admission as the risk of AKI in mild COVID-19 or asymptomatic SARS-CoV-2 infection remains unknown.

2.2 COVID-19 and CKD

Individuals are primarily diagnosed with CKD when they are found to have an estimated glomerular filtration rate (GFR) (eGFR) persistently < 60ml/min/1.73m^2 over at least three months. eGFR is usually calculated from serum creatinine, an easily assayed endogenous marker of kidney function but one that is affected by a range of factors related to both its synthesis and excretion such as age, sex, diet, muscle mass, renal tubular secretion, gut loss, muscle catabolism and genetic ancestry[5]. CKD can also be diagnosed by urinary albumin creatinine ratio >3 mg/mmol or in the presence of a structural kidney abnormality (e.g. polycystic kidneys or dysplastic kidneys).

Trajectories of eGFR can vary and progressive decline to <15 ml/min/1.73m^2 results in end-stage renal disease (ESRD) (also known as “kidney failure”) with long-term KRT required to sustain life (in the form of dialysis or kidney transplantation). In addition to ESRD, CKD is associated with increased all-cause mortality, cardiovascular mortality and morbidity, and AKI[6,7]. Complications of CKD include anaemia, hyperkalaemia, hypervolaemia, altered pharmacokinetics, and secondary hyperparathyroidism[7]. CKD is common, affecting 8.5% of the UK population[8], and is associated with a substantial burden on healthcare costs[9].

AKI is a risk factor for CKD[6]. After SARS-CoV-2 infection, tubular injury, de novo immune-mediated processes (such as collapsing glomerulopathy[10-12]), or direct infection of kidney parenchyma might independently cause additional insults leading to long-term kidney

disease. In addition, pre-existing CKD is an independent risk factor for severe outcomes in COVID-19, plausibly leading to accelerated renal decline in those most at risk[13].

Given the scale of COVID-19 hospitalisation, including COVID-19-related-AKI, there is a pressing need to understand whether this will lead to an increased burden of CKD and its complications on health systems, given the potential stresses on resources that these will incur.

There has been a gradual increase in the number of studies focusing on post-COVID-19 complications, and some of these include the incidence or progression of kidney diseases[14,15].

2.3 Existing research

The strongest evidence to date comes from a cohort study of US military veterans with over 89,000 SARS-CoV-2 survivors and over 1.6 million non-infected military veteran controls. This study demonstrated increased incidence of subsequent AKI, eGFR decline ≥50%, ESRD, and major adverse kidney events (defined as eGFR decline ≥50%, ESRD, or all-cause mortality), irrespective of whether individuals were non-hospitalised, hospitalised, or admitted for critical care[16]. Overall, ESRD rates were almost three times higher in survivors compared to individuals without known infection.

As this study compared SARS-CoV-2 survivors to contemporaneous individuals without known infection, it is possible that many of this comparator group were misclassified due to the reduced availability of testing in the early stages of the pandemic and asymptomatic infection, meaning that the strength of associations may be underestimated. Findings in military veterans may also have limited generalisability in the wider population (e.g. >90% of the overall cohort was male).

Increased long-term kidney outcomes in US military veterans not hospitalised after COVID-19 warrant further investigations. These findings are in contrast to a cohort study of 443 adults from Hamburg, Germany who had survived SARS-CoV-2 infection (over 90% of

whom had not been hospitalised), which found that eGFR was similar a median of 10 months after infection compared to that of matched population-based controls recruited before the pandemic (median eGFR 108.9 vs 109.1 ml/min/1.73m^2)[17]. The generalisability of this study may also be limited as some participants after SARS-Cov2 infection were recruited through public announcement rather than systematic sampling.

A study of patients from Wuhan, China who had survived COVID-19 hospitalisation investigated follow-up eGFR in an outpatient clinic after discharge, reporting that 35.0% of over 1300 patients had eGFR <90 ml/min/1.73m^2 (median follow-up 153 days)[18]. However, the findings are difficult to interpret because of the absence of pre-hospitalisation eGFR data (i.e. before COVID-19 illness), and the high threshold used for defining abnormal kidney function (eGFR <90 ml/min/1.73m^2). Inpatient data from the time of COVID-19 illness found that a similar proportion of those without AKI had eGFR <90 ml/min/1.73m^2, which may mean that the findings merely reflect the pre-existing prevalence of abnormal kidney function in the study population. Of survivors without AKI who had preserved eGFR during admission, 13.0% of those who attended follow-up were found to have new eGFR <90 ml/min/1.73m^2. However, eGFR below a lower threshold (e.g. <60 or <30 ml/min/1.73m^2)[19], time taken to reach percentage decline in eGFR[16,20], and longitudinal eGFR decline using linear mixed models[21] are likely to be more informative than eGFR <90 ml/min/1.73m^2 in evaluating kidney outcomes following infection.

The pathophysiological processes by which COVID-19 might lead to declining kidney function remain unknown. Autopsy studies of patients who have died with COVID-19 suggest that SARS-CoV-2 may directly infect the kidney, causing upregulation of profibrotic cell signalling pathways[22]. However, since pre-existing CKD is a risk factor for death from severe COVID-19, the associations between COVID-19 and the histological features of CKD at autopsy may be distorted as there are difficulties inferring temporality: whether histological changes seen on autopsies were caused by COVID-19 or were present because people with CKD are more likely to die from COVID-19.

We will investigate the association between SARS-CoV-2 and long-term kidney outcomes using general population data on the OpenSAFELY-TPP platform, comprising

approximately 40% of the English adult population, We are well-placed to improve understanding of associations with greater precision, and are using a range of methods to address systematic biases.

3. Objectives

Overall, our aim is to investigate long-term kidney outcomes after recorded SARS-CoV-2 infection in the general population.

Our specific objectives are:

1. To describe the relative and absolute rates of ESRD (primary outcome) after recorded SARS-CoV-2 infection compared to both contemporary and historical groups, stratified by:
    a. Hospitalisation for COVID-19
    b. Critical care admission for COVID-19
    c. Mechanical ventilation for COVID-19
    d. Acute KRT associated with COVID-19
    e. AKI associated with COVID-19
2. To describe the relative and absolute rates of a 50% reduction in eGFR, incident AKI, and death (secondary outcomes), after recorded SARS-CoV-2 infection compared to both contemporary and historical groups, stratified by:
    a. Hospitalisation for COVID-19
    b. Critical care admission for COVID-19
    c. Mechanical ventilation for COVID-19
    d. Acute KRT associated with COVID-19
    e. AKI associated with COVID-19
3. To describe the relative and absolute rates of these outcomes after hospitalisation for COVID-19 compared to a historical hospitalised group, stratified by:
    a. Critical care admission for COVID-19
    b. Mechanical ventilation for COVID-19

    c. Acute KRT associated with COVID-19
    d. AKI associated with COVID-19
4. To describe the relative and absolute rates of outcomes after recorded SARS-CoV-2 infection compared to both contemporary and historical groups stratified by:
    a. Baseline eGFR
    b. Diabetes
    c. Age
    d. Sex
    e. Ethnicity
    f. Socioeconomic deprivation
5. To describe the relative and absolute rates of outcomes after recorded SARS-CoV-2 infection compared to both contemporary and historical groups additionally stratified by:
    a. Calendar time
    b. Vaccination status

4. Methods

We will use routinely collected electronic health record (EHR) data from primary care practices using TPP SystmOne software, covering approximately 40% of the population in England, linked at the individual level to NHS Secondary Uses Service (SUS) data on hospitalisations, and Second Generation Surveillance System (SGGS) data on SARS-CoV-2 test results. We will conduct cohort studies investigating kidney outcomes in people after recorded SARS-CoV-2 infection compared to both contemporary (2020-2021 general population) and historical (2018-2019 general population) groups.

4.1 Data source

Primary care records managed by TPP, a GP software provider, were linked to other data sources through OpenSAFELY, a data analytics platform created by our team on behalf of

NHS England to address urgent COVID-19 research questions (https://opensafely.org) (Williamson 2020). OpenSAFELY provides a secure software interface allowing the analysis of pseudonymized primary care patient records from England in near real-time within the EHR vendor's highly secure data centre, avoiding the need for disclosive pseudonymized patient data to be transferred off-site. This, in addition to other technical and organisational controls, minimizes any risk of re-identification. Similarly, pseudonymized datasets from other data providers are securely provided to the EHR vendor and linked to the primary care data. The dataset analysed within OpenSAFELY is based on 24 million people currently registered with GP surgeries using TPP SystmOne software. It includes pseudonymized data such as coded diagnoses, medications, and physiological parameters. No free text data are included. Further details on our information governance can be found here: information governance and ethics.

4.2 Information governance

NHS England is the data controller; TPP is the data processor; and the key researchers on OpenSAFELY are acting on behalf of NHS England. This implementation of OpenSAFELY is hosted within the TPP environment, which is accredited to the ISO 27001 information security standard and is NHS IG Toolkit compliant (NHS Digital Data Security Standards, NHS Digital Data Security & Protection Toolkit); patient data have been pseudonymized for analysis and linkage using industry standard cryptographic hashing techniques; all pseudonymized datasets transmitted for linkage onto OpenSAFELY are encrypted; access to the platform is through a virtual private network (VPN) connection; the researchers hold contracts with NHS England and only access the platform to initiate database queries and statistical models; all database activity is logged; and only aggregate statistical outputs leave the platform environment following best practice for anonymization of results such as statistical disclosure control for low cell counts (NHS Digital Anonymisation standard for publishing health and social care data). The OpenSAFELY research platform adheres to the data protection principles of the UK Data Protection Act 2018 and the EU General Data Protection Regulation (GDPR) 2016. In March 2020, the Secretary of State for Health and

Social Care used powers under the UK Health Service (Control of Patient Information) Regulations 2002 (COPI) to require organisations to process confidential patient information for the purposes of protecting public health, providing healthcare services to the public and monitoring and managing the COVID-19 outbreak and incidents of exposure (Department of Health and Social Care Coronavirus (COVID-19): notification to organisation to share information). Together, these provide the legal bases to link patient datasets on the OpenSAFELY platform. GP practices, from which the primary care data are obtained, are required to share relevant health information to support the public health response to the pandemic and have been informed of the OpenSAFELY analytics platform.

4.3 Study designs

We will compare people after SARS-CoV-2 infection to both historical and contemporary cohorts in order to address epidemiological challenges and triangulate meaningful conclusions.

Testing for SARS-CoV-2 during the early stages of the pandemic was restricted due to a lack of infrastructure[23]; therefore many people who were infected (both symptomatic and asymptomatic) will not have been coded as such and so will be misclassified in a contemporary comparison. Using historical comparator groups will allow us to definitively ensure that the comparator group was not infected with SARS-CoV-2 during follow-up. Some comparisons will also be matched on date (i.e., to start follow-up on the same date two years before), which will allow us to account for consistent seasonal variations[24,25].

Contemporary comparator populations will account for potential biases we anticipate for any historical comparison. Using a contemporary comparator will also allow us to finely adjust for changes over time during the pandemic (with time since the start of the pandemic as the underlying timescale), such as the emergence of SARS-CoV-2 variants[26], changes in testing policy[23], changes in treatment of COVID-19[27], and differences in ascertainment of outcomes due to reduced access to healthcare (including blood bottle shortages in 2021)[28,29].

4.4 Study populations

Our overall study population will be all adults registered with primary care practices using TPP SystmOne software (Figure 1) since 1 February 2016. We will exclude all individuals with missing age, sex, or Index of Multiple Deprivation (IMD) as these are likely to indicate poor data quality. All individuals included will need to have at least three months of follow-up available before cohort entry to ensure reliable capture of baseline health status; we have been reassured by TPP that three months is sufficient for records to be updated and this will optimise power.

4.4.1 Exposure group

Our exposure group will comprise people with recorded SARS-CoV-2 infection identified using data from SGSS, primary care records or SUS who remain alive 28 days after the first recording of infection (i.e. 28 days after date of testing positive in SGSS, coding in primary care records, or admission on SUS) (index date) (Figure 1).

Anyone coded in primary care or SUS to be receiving chronic dialysis or kidney transplant or with eGFR <15 ml/min/1.73m^2 (i.e. anyone with ESRD or CKD Stage 5) at the time of first recording of SARS-CoV-2 infection will be excluded.

A secondary analysis will investigate individuals who remain alive 28 days after the date of first hospital admission coded for COVID-19 in SUS. Anyone coded in primary care or SUS to be receiving chronic dialysis or kidney transplant or with eGFR <15 ml/min/1.73m^2 (i.e. anyone with ESRD or CKD Stage 5) at the time of hospital admission will be excluded.

4.4.2 Historical comparator groups

For our main analysis, historical comparator groups will comprise individuals from the general population under follow-up in OpenSAFELY, each matched five to one to each

individual from the recorded SARS-CoV-2 group on age (within 2 years), sex, NHS Sustainability and Transformation Partnership (STP, geographical areas configured for regional reorganisation available in our data), IMD decile (a measure of socioeconomic deprivation based on postcode), and date (i.e. 2 years before the first recording of SARS-CoV-2 infection in the exposed case) (Figure 1). Anyone coded in primary care or SUS to be receiving chronic dialysis or kidney transplant or with eGFR <15 ml/min/1.73m^2 before this date will be excluded (i.e. anyone with ESRD or CKD Stage 5). We will match without replacement (i.e. individuals in the historical comparator group will not be matched to more than one individual with SARS-CoV-2 infection) in order to optimise statistical precision.

In a secondary analysis, we will compare individuals who remain alive 28 days after admission to hospital with COVID-19 with those who remained alive 28 days after admission to hospital for pneumonia (including influenza) from 1 February 2018 up to two years before the end of follow-up for the exposed group (unmatched) (Figure 1). A previous study done using OpenSAFELY found similarities in outcomes between people discharged after hospitalisation with COVID-19 and a historical comparator group discharged after pneumonia[30]. Individuals who survived COVID-19 and hospitalisation from pneumonia in 2018-2019 will be followed-up in both the exposed and unexposed groups.

4.4.3 Contemporary comparator group

For our main analysis, contemporary comparator groups will comprise individuals from the general population under follow-up in OpenSAFELY, individually matched five to one to each individual from the recorded SARS-CoV-2 group on age (within 2 years), sex, NHS STP, IMD decile, and date of first recording of SARS-CoV-2 infection (Figure 1). Anyone coded in primary care or SUS to be receiving chronic dialysis or kidney transplant or with eGFR <15 ml/min/1.73m^2 before the first recording of SARS-CoV-2 infection will be excluded (i.e. anyone with ESRD or CKD Stage 5). We will again match without replacement. Individuals from this group will be censored if they themselves are recorded

with SARS-CoV-2 infection and further follow-up will commence in the exposed group from 28 days after recording if they remain alive (Figure 1).

4.4.4 Follow-up start

By using index dates 28 days after first recording of SARS-CoV-2 infection (i.e., date of testing in SGSS, date of primary care coding, or date of admission on SUS), we will be able to look back and further stratify the exposure by measures of SARS-CoV-2 severity (i.e. community, hospitalisation, critical care admission etc.).

For analyses comparing people after SARS-CoV-2 infection to a historical comparator group from 2018-2019, the matched historical comparators will be followed-up from exactly two years before the index date.

For analyses comparing people hospitalised for COVID-19 with an unmatched historical group of individuals hospitalised for pneumonia from 2018-2019, we will follow all eligible individuals from 28 days after their admission date for pneumonia.

For analyses comparing people after SARS-CoV-2 infection to a matched contemporary comparator group, we will follow all eligible individuals from the index date.

4.4.5 Follow-up end

In all analyses we will follow individuals until the earliest of: record of specific kidney outcome under investigation, ESRD, death, no longer registered with GP practice, or study end. Individuals from the matched contemporary comparator group will also be censored at the date of first recorded SARS-CoV-2 infection and will transfer to the exposed group if they survive 28 days after this date.

For analyses using matched historical 2018-2019 comparators, the study end for people with COVID-19 will be the last available date for data at the time of extraction, and for

their matched comparators it will be January 31 2020 at the latest. Our choice of end of follow-up for people with COVID-19 is to be consistent with the available follow-up time for people in the historical comparator group, as we will no longer be able to be able to follow people in the historical comparator group after February 2020 (as we will no longer be sure that those in our comparator group did not have COVID-19 after the start of the initial UK COVID-19 outbreak in February 2020). By allowing the same duration of potentially available follow up for people with and without COVID we will be able to ensure our results are not biased by allowing additional follow up for people with COVID-19 (and consequently allowing more time for them to develop the outcomes under investigation) compared to those without. By allowing the same duration of potentially available follow up for people with and without SARS-CoV-2 infection, we will be able to ensure our results are not biased by allowing additional follow up for people pre-pandemic.

For analyses with the contemporary comparator group, the study end will be the latest available date of fully linked data available on the day the initial cohort is extracted.

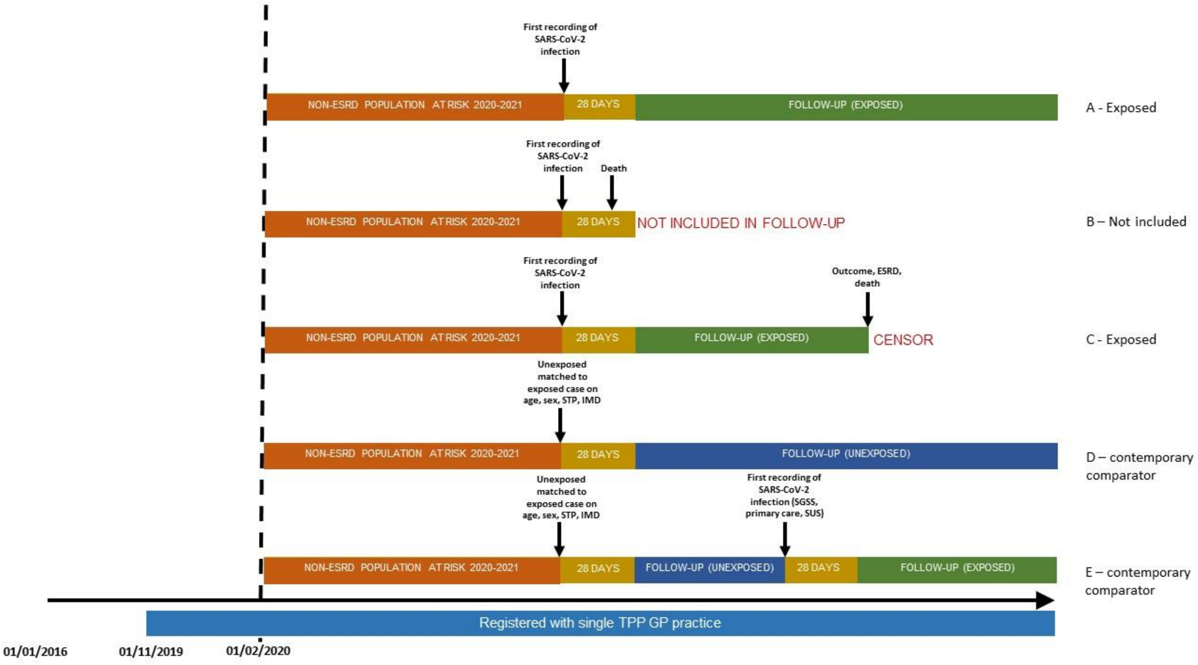

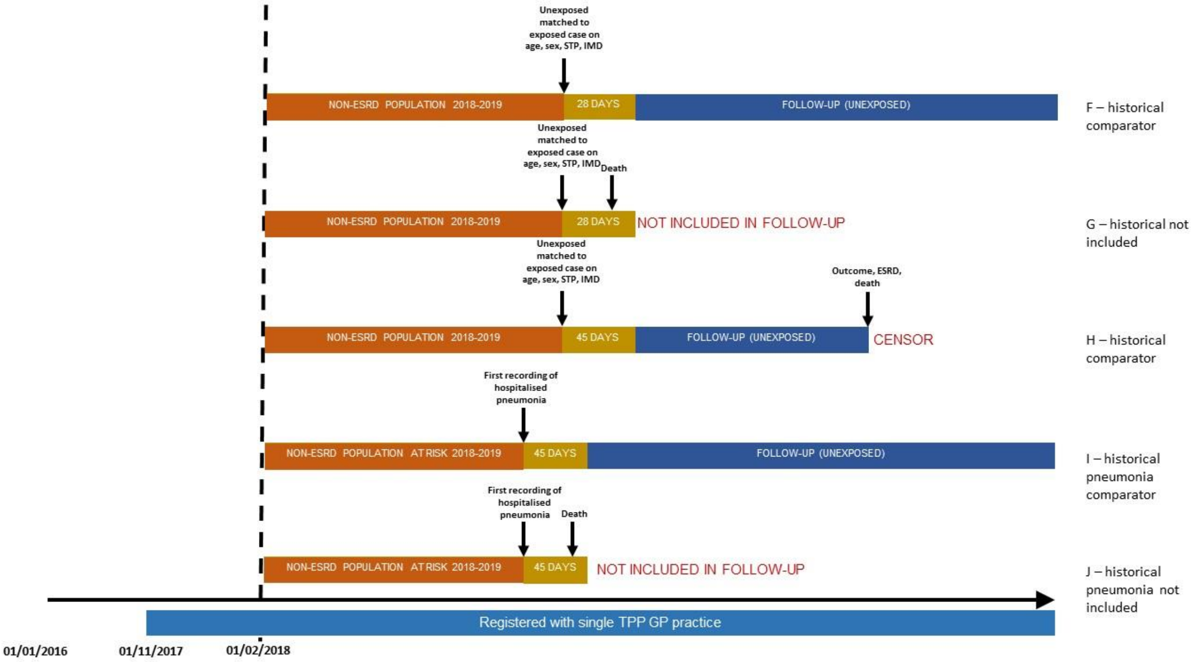

Figure 1 Graphical illustrations of potential study scenarios. **A-C: Examples of individuals with recorded SARS-CoV-2 infection.** A was followed-up from 28 days after SARS-CoV-2 infection until the study conclusion; B died within 28 days of SARS-CoV-2 infection so was not followed-up; C commenced follow-up from 28 days after SARS-CoV-2 infection until they were censored (i.e. for outcome event, death, or ESRD). **D-E: Examples of matched contemporary comparators.** D was matched to an individual with SARS-CoV-2 infection and followed-up from 28 days after matching until the study conclusion; E was matched to an individual with SARS-CoV-2 infection and followed-up from 28 days after matching until they were first recorded with SARS-CoV-2 infection themselves when they were censored from further follow-up as an unexposed comparator and then followed-up as an exposed case from 28 days after infection until the study conclusion. **F-G: Examples of matched historical comparators from 2018-2019.** F was matched to an individual with SARS-CoV-2 infection and followed-up from 28 days after matching until the study conclusion; G died within 28 days of matching so was not followed-up; H commenced follow-up from 28 days after matching until they were censored (i.e. for outcome event, death, or ESRD). **I-J: Examples of unmatched historical comparators hospitalised for pneumonia from 2018-2019.** I was followed-up from 28 days after admission for pneumonia until the study conclusion. J died within 28 days of admission for pneumonia so was not followed-up.

4.5 Study measures

4.5.1 Exposure

We will identify recorded SARS-CoV-2 infection using data from SGSS, primary care records and SUS. We will include all codes indicative of SARS-CoV-2 infection. SGSS contains the date of an individual's earliest positive SARS-CoV-2 test results from Pillar 1

(NHS and Public Health England laboratories) and Pillar 2 (commercial partners) between 1 February 2020 to the most recent available time point (i.e. the COVID-19 pandemic era).

Individuals meet criteria for inclusion in the COVID-19 exposed group if they:

- Tested positive for SARS-CoV-2 infection in SGSS
- Had a primary care code indicative of SARS-CoV-2 infection
- Discharged from hospital with an International classification of Diseases Version 10 (ICD-10) code in SUS indicative of COVID-19

The index date for those exposed to COVID-19 will be from 28 days after first recording of SARS-CoV-2 infection (i.e., after the earliest date of the record of a positive test in SGSS, primary care coding, or admission in SUS).

Looking at all identified SARS-CoV-2 infections will allow us to investigate how many adverse kidney outcomes can be prevented by preventing SARS-CoV-2 infection altogether through comparisons with people from the general population without known infection. We will also be able to investigate whether outcomes vary based on infection severity (hospitalisation, critical illness, mechanical ventilation and AKI [including requiring RRT]) as well as calendar time and vaccination status through further stratification based on morbidity coding (ICD-10 codes) from SUS. We will categorise people as i) unvaccinated if they had not received any vaccine dose at least 7 days before first recording of SARS-CoV-2 infection, ii) single dose vaccinated if they had received one vaccine dose at least 7 days before first recording of SARS-CoV-2 infection, iii) double vaccinated if they had received two vaccine doses at least 7 days before first recording of SARS-CoV-2 infection, and iv) more than double vaccinated if they had received more than two vaccine doses (including booster doses) at least 7 days before first recording of SARS-CoV-2 infection.

Hospitalised COVID-19 cases will be further investigated through a separate comparison with a historical group. Additional levels of exposure for those hospitalised will include critical care admission, mechanical ventilation, acute KRT and AKI, determined from ICD-10 and procedural codes in SUS.

4.5.2 Outcomes

4.5.2.1 Primary outcome

We will identify incident ESRD through codes for dialysis or kidney transplant using either primary care codes, or ICD-10 or procedural codes from SUS, or eGFR <15ml/min/1.73m^2 in primary care.

4.5.2.2 Secondary outcomes

1. 50% reduction in eGFR

This analysis will exclude anyone without a baseline eGFR measurement. We will be cautious when interpreting our analysis of eGFR outcomes as this is unlikely to be generalisable to the general population and only to people who had an indication for baseline eGFR testing (e.g. comorbidities, drugs).

Baseline eGFR will be defined as the mean primary care eGFR calculated from serum creatinine using the CKD-EPI equation from 18 months before the index date up to 14 days before the index date (or 14 days before hospital admission date for hospitalised COVID-19 cases)[31].

Primary care eGFR measurements will be followed-up monthly from the index date, until criteria for the outcome endpoint is met (50% reduction in eGFR from the baseline eGFR). The OpenSAFELY platform is currently only able to extract a predefined number of measures for any variable (i.e., it is not possible to extract all available measures); we will therefore extract and use the last eGFR measure for every calendar month in follow-up. Incident ESRD (i.e. dialysis or kidney transplant primary care codes, or ICD-10 or procedural codes from SUS, or eGFR <15ml/min/1.73m^2 in primary care) will also be included in the outcome definition.

We will also consider assessing changes in eGFR trajectory using slopes.

2. AKI

We will identify episodes of hospitalisation with AKI through ICD-10 codes in any diagnostic position in SUS after the index date.

3. Death

We will describe rates of death as this is an important competing risk for outcomes. Date of death will be obtained using primary care records, which compared to the Office for National Statistics (ONS) (the gold standard), has previously been demonstrated to have 78% exact agreement and 99% agreement within 30 days[32]. In case such accurate date coding practices have been affected by the pandemic, we will be able to link to the ONS date of death for 2020 data (we do not have access to ONS date of death linkage before 2019).

4.5.3 Covariates

Covariates have been determined using a directed acyclic graph (DAG) (Figure 2): demographic (age, sex), geographic region, deprivation, ethnicity, smoking, body mass index (BMI) and existing comorbidities.

For the matched historical comparison groups, we will extract covariable data as of 28 days before the index date (i.e. on the date of first recording of infection) on OpenSAFELY.

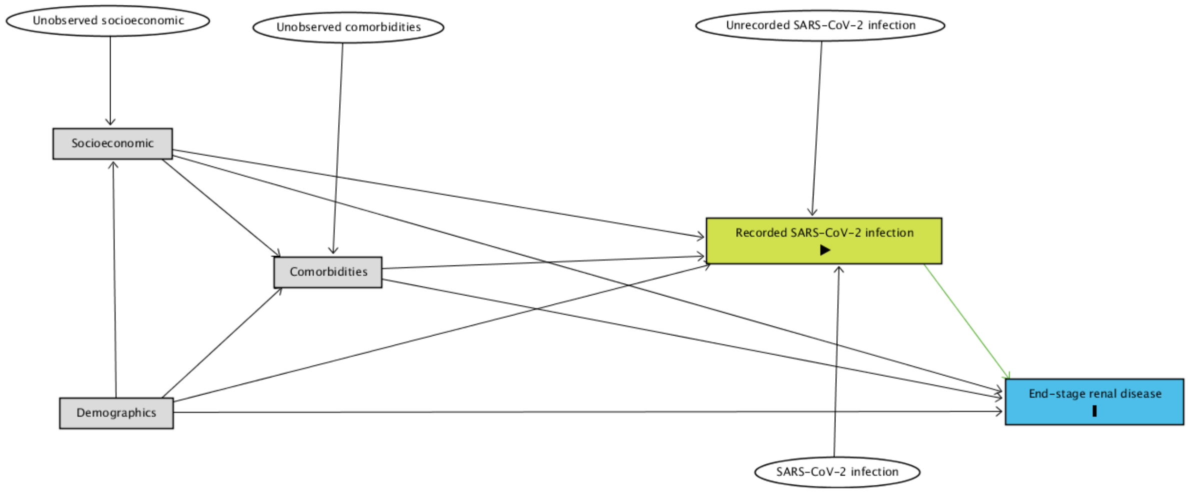

Figure 2 Causal diagram for SARS-CoV-2 infection and end-stage renal disease

4.5.3.1 Demographic

Age and sex will be included in all analyses. For descriptive purposes, we will classify age in the following categories: 18-39, 40-49, 50-59, 60-69, 70-79 and 80 years and over. In regression analyses, we will include a spline function for age to account for variation in SARS-CoV-2 infection by age.

4.5.3.2 Social

It is important to account for geographical variation in SARS-CoV-2 infection rates. We will define an individual's geographical region based on NHS STP administrative regions of England grouped into East of England, London, Midlands, North East and Yorkshire, North West, South East and South West.

We will define deprivation using quintiles of IMD. IMD is assessed across seven domains of deprivation: income, employment, education, health, crime, barriers to housing and services,

and living environment. The IMD indices rank each “lower layer super output area”, derived from residential postcodes in England, from most deprived to least deprived.

We will define ethnicity using self-reported ethnicity recorded in primary care and collapsed into four high-level categories of: white, south Asian, black, and other and mixed[33]. We expect that ethnicity will be missing for approximately 20% of our study population[33,34]. For analyses adjusting for ethnicity, we will do a complete-case analysis (only including those with known ethnicity) with a follow-up sensitivity analysis.

4.5.3.3 Clinical

We will describe several clinical conditions including: cancer, cardiovascular diseases, chronic liver disease, diabetes mellitus, hypertension and immunosuppressive diseases.

We will describe baseline eGFR for each individual based on the mean eGFR recoded from 18 months to 14 days before the index date. As with follow-up eGFR, it is not possible to obtain infinite serial eGFR measurements from OpenSAFELY and so the final eGFR measurement of each calendar month before the index date (up to 18 months before the cohort start date) will be recorded for each individual. eGFR will be calculated using the CKD-EPI equation using serum creatinine measurements without adjustment for ethnicity[31]. Baseline eGFR will be classified as follows:

1. ≥105 ml/min/1.73m^2
2. 90-104 ml/min/1.73m^2
3. 75-89 ml/min/1.73m^2
4. 60-74 ml/min/1.73m^2
5. 45-59 ml/min/1.73m^2 (CKD stage 3A)
6. 30-44 ml/min/1.73m^2 (CKD stage 3B)
7. 15-29 ml/min/1.73m^2 (CKD stage 4)
8. No eGFR measurement

We will define smoking status based on most recent primary care coding as non-smokers, ex-smokers or current smokers. We will assume that individuals with no record of smoking status are non-smokers as smoking is less common than not smoking (approximately 14% of the UK adult population were smokers in 2019)[35]. However, we will run a sensitivity analysis restricting to those with known smoking status.

BMI will be calculated using the most recent height and weight closest to the index date, then categorised as: underweight (<18.5kg/m^2), normal weight (18.5-24.9kg/m^2), overweight (25.0-29.9kg/m^2), obese (≥30.0kg/m^2) and undocumented (assumed to be normal in main analyses)[36,37].

4.6 Statistical analysis

We will initially describe the baseline characteristics of people after SARS-CoV-2 infection and those from the matched historical and contemporary comparator groups.

4.6.1 Descriptive statistics

We will describe the baseline characteristics of people after SARS-CoV-2 infection, stratified by severity, and those from the matched historical and contemporary comparator groups.

4.6.2 Main analysis

Our statistical analysis plan has been informed by our DAG (**Figure 2**). The DAG has been developed based on assumptions about the causal relationship between SARS-CoV-2 infection and the study outcomes, although it may be limited by currently incomplete understanding. The DAG includes unobserved factors which may be sources of residual confounding (such as socioeconomic factors and comorbidities), as well as sources of

outcome misclassification (i.e. unobserved eGFR decline) and death post-SARS-CoV-2 infection as a competing outcome.

We will use Cox regression models to estimate cause-specific hazard ratios and 95% confidence intervals for ESRD for individuals after SARS-CoV-2 infection, stratified by hospitalisation, critical care admission, mechanical ventilation, acute KRT and AKI, compared to individuals in the i) historical and ii) contemporary comparator groups. The comparator groups have been matched on age, sex, region, socioeconomic deprivation and date.

On the basis of minimum sufficient adjustment sets from our DAG, we will run sequential regression models, i) adjusted for age, sex, socioeconomic deprivation, ethnicity, smoking and BMI and, ii) additionally adjusted for baseline eGFR, cancer, cardiovascular diseases, chronic liver disease, diabetes mellitus, hypertension and immunosuppressive diseases.

We will use robust standard errors to account for clustering by GP practice as well as on individuals as there may be overlap between those in the recorded SARS-CoV-2 infection groups and the historical comparison group (although the time periods are non-overlapping). We will assume people with missing BMI are not obese and people without a documented smoking status are non-smokers[37].

4.6.3 Secondary analyses

1. We will use Cox regression models to estimate cause-specific hazard ratios and 95% confidence intervals for the secondary outcomes i) 50% reduction in eGFR, ii) AKI and iii) death for individuals after SARS-CoV-2 (stratified as above) compared to individuals in the i) historical and ii) contemporary comparator groups.
2. We will undertake further Cox regression models for both primary and secondary outcomes with SARS-CoV-2 infection stratified by calendar time (i.e. February –

June 2020, July – September 2020, October – November 2020, December 2020 – February 2021, and March – January 2022) and vaccination status.

3. We will fit interaction terms to obtain cause-specific hazard ratio estimates for both primary and secondary outcomes stratified by age group, ethnicity, socioeconomic deprivation, diabetes and baseline eGFR.
4. For people who survive 28 days after hospitalisation for COVID-19, we will undertake an unmatched cohort analysis comparing kidney outcomes with a historical population followed-up from 1 February 2018 restricted to those who survived 28 days after a hospital admission for pneumonia (with or without influenza). This analysis will be stratified by critical care admission, mechanical ventilation, acute KRT and AKI, with additional adjustment for calendar month. The reason for this analysis is to investigate whether COVID-19 is responsible for long-term kidney outcomes to a greater or lesser extent than hospitalisation for other respiratory tract infections.

4.6.4 Sensitivity analyses

We aim to investigate the robustness of our primary and secondary analyses by exploring assumptions further using a range of sensitivity analyses, including:

1. Competing risk analysis

   Due to the competing risk of death, we will undertake a competing risk analysis using Fine and Gray regression models to obtain subdistribution hazard ratios for ESRD.
2. Multiple imputation for ethnicity

In our main analyses, we will undertake a complete case analysis for ethnicity (i.e. only include individuals with recorded ethnicity data). In this sensitivity analysis, we will use multiple imputation for missing ethnicity data.

3. Complete case analysis for smoking and BMI

   In our main analysis, we will assume that individuals without smoking status are non-smokers and that individuals without BMI have normal BMI. In separate sensitivity analyses, we will instead undertake complete case analyses (i.e. only including individuals with complete smoking and BMI data respectively).

4. ESRD definition

   In case of possible residual misclassification of AKI as ESRD (where an individual has an eGFR <15ml/min/1.73m^2), we will undertake a sensitivity analysis restricting only to people with new codes for dialysis or transplantation (i.e., incident KRT).

5. Incident eGFR <30ml/min/1.73m^2

   As healthcare services have been impacted heavily by the pandemic, there may not have been sufficient routine testing of patients to be able to detect a reduction in eGFR. As patients who are highest risk may be prioritised, we will also investigate for incident eGFR <30ml/min/1.73m^2 in primary care or new codes for dialysis or kidney transplant in either primary care records, or ICD-10 or procedural codes from SUS.

6. eGFR equation

   In our main analyses, we will calculate eGFR using the CKD-EPI equation from 2009 (without adjusting for ethnicity)[31]. This equation has recently been updated excluding adjustment for ethnicity[38]. We will assess how many individuals change CKD stage from the original CKD-EPI equation after switching to the 2021 equation. If there are marked differences in the number of people with CKD and within each CKD stage, we will undertake a sensitivity analysis using the 2021 equation instead for eGFR.

4.7 Software and Reproducibility

Data management will be undertaken using Python, with analysis carried out using Stata 16.1. Code for data management and analysis and code lists used to define study measures (exposures, outcomes, covariates) will be archived online: https://github.com/opensafely/post-covid-kidney-outcomes.

5. Power calculations

A study of 89,000 survivors after SARS-CoV-2 infection found increased incidence of ESRD with a hazard ratio of 2.96 (95% confidence interval 2.49-3.51).

For our power calculation, we have assumed conservatively that the effect is only found in people hospitalised with COVID-19 and that the incidence of ESRD in the general population is 0.02%. There have been over 690,000 people hospitalised for COVID-19 in England with 150,000 deaths. As OpenSAFELY-TPP comprises 40% of the population in England, after accounting for insufficient time registered with a TPP practice and missing age, sex, deprivation and ethnicity data in people hospitalised for COVID-19 based on another study, we anticipate that our sample will include over 200,000 hospitalised COVID-19 survivors for analysis[34]. This sample size would be sufficient to detect a hazard ratio for incident ESRD >1.6 with >80% power at the 5% significance level.

**Total exposed population (SARS-CoV-2 infection survivors)**	**Hazard ratio for incident ESRD**					
	**1.5**	**1.6**	**1.7**	**1.8**	**1.9**	**2.0**
**100,000**	50%	63%	74%	83%	89%	93%
**150,000**	65%	78%	88%	94%	97%	99%
**200,000**	76%	88%	94%	98%	99%	100%
**250,000**	84%	93%	98%	99%	100%	100%
**500,000**	98%	100%	100%	100%	100%	100%
**1,000,000**	100%	100%	100%	100%	100%	100%

Table 1 Power estimates for detecting incident ESRD in survivors of SARS-CoV-2 infection at the 5% significance level by total exposed population size, assuming an incidence of ESRD of 0.02% in the general population.

6. Strengths and limitations

Using OpenSAFELY-TPP data offers the statistical power to investigate for relatively rare outcomes such as ESRD. In addition, we hope to improve understanding about long-term kidney outcomes after SARS-CoV-2 infection through triangulation, integrating findings from different approaches each of which seeks to address important biases[39]. We have explicitly presented several potential biases through a DAG[40].

We will use a historical comparator group in order to definitively ensure that individuals who were infected with SARS-CoV-2 are not included in follow-up, and most comparisons will be matched on date (i.e. to start follow-up on the same date two years earlier) to account for consistent seasonal variations[24,25]. Testing for SARS-CoV-2 during the early stages of the pandemic was restricted due to a lack of infrastructure[23] and so many people who were infected (both symptomatic and asymptomatic) will not have been coded as such and so will be misclassified in contemporary comparisons.

However, a historical comparator population may also introduce biases as it will not allow us to account for differences in the ascertainment of outcomes due to reduced access to healthcare during the pandemic (including blood bottle shortages in 2021)[28,29]. By also using a contemporary comparator population, we will be able to finely adjust for changes over time during the pandemic.

Investigating eGFR outcomes may be affected by collider bias as only people with available eGFR measurement will be included and eGFR measurement may be more likely to be undertaken in those at risk of CKD, which may differentially include people who have survived more severe COVID-19[41,42]. In addition, as equations for eGFR are unable to account for body composition and nutritional factors, eGFR outcomes may also be underestimated if there are sustained changes after surviving a prolonged, severe or complex course of illness with COVID-19. A study from Wuhan, China, found that of those without AKI with eGFR <90 ml/min/1.73m^2 during hospitalisation, 29.7% of those followed-up in an outpatient clinic were found to have eGFR >90 ml/min/1.73m^2

suggesting potential decrease in serum creatinine in some people beyond hospital discharge[18]. Similar findings were seen in a study of patients hospitalised with AKI in London, UK[43]. In one of our secondary analyses, we will restrict only to individuals hospitalised for COVID-19 and a historical group hospitalised for pneumonia to account for these biases to some extent. We will also investigate for ESRD and hospitalised AKI as other outcomes which are less susceptible to these bias, so conclusions will not exclusively be based on eGFR outcomes.

Analyses restricting to individuals hospitalised for COVID-19 and a historical group hospitalised for pneumonia will also allow us to determine whether differences in outcomes are due to COVID-19 rather than being the effects of hospitalisation for acute respiratory illness. Another study conducted on OpenSAFELY (peer-reviewed publication pending) found that outcomes (such venous thromboembolism, myocardial infarction and stroke) in patients discharged after hospitalisation for COVID-19 was similar to those after hospitalised pneumonia in a historical comparison[30]. However, I will be cautious about interpreting findings as people who are both susceptible to hospitalisation with COVID-19 and subsequently surviving, may be different to people who survive hospitalisation for pneumonia.

7. Administrative

7.1 Ethics

The study was approved by the Health Research Authority (REC reference 20/LO/0651) and by the London School of Hygiene and Tropical Medicine (LSHTM) ethics board (reference 21863).

7.2 Funding

[Placeholder for OpenSAFELY overall funding]

[Enter details of local funding] NCS?

VM is funded by the National Institute for Health Research (Award ID NIHR301535).

7.3 Patient and public involvement

This protocol has been developed in collaboration with our Patient and Public Involvement partners.

7.4 Conflict of interests

The authors have declared no competing interest.

References

1 KDIGO Clinical Practice Guideline for Acute Kidney Injury. *Kidney International Supplements* **2**, 1–138, doi:doi:10.1038/kisup.2012.6 (2012).

2 Phillips, D. *et al*. The influence of socioeconomic status on presentation and outcome of acute kidney injury. *QJM: An International Journal of Medicine* **111**, 849-857, doi:10.1093/qjmed/hcy180 (2018).

3 Guan, W. J. *et al*. Clinical Characteristics of Coronavirus Disease 2019 in China. *N Engl J Med* **382**, 1708-1720, doi:10.1056/NEJMoa2002032 (2020).

4 Sullivan, M. K. *et al*. Acute kidney injury in patients hospitalized with COVID-19 from the ISARIC WHO CCP-UK Study: a prospective, multicentre cohort study. *Nephrol Dial Transplant* **37**, 271-284, doi:10.1093/ndt/gfab303 (2022).

5 Gama, R. M., Kalyesubula, R., Fabian, J. & Mahalingasivam, V. NICE takes ethnicity out of estimating kidney function. *BMJ* **374**, n2159, doi:10.1136/bmj.n2159 (2021).

6 Forni, L. G. *et al.* Renal recovery after acute kidney injury. *Intensive Care Med* **43**, 855-866, doi:10.1007/s00134-017-4809-x (2017).

7 KDIGO 2012 Clinical Practice Guideline for the Evaluation and Management of Chronic Kidney Disease. *Kidney International Supplements* **3**, 1–150, doi:doi:10.1038/kisup.2012.76 (2013).

8 Stevens, P. E. *et al.* Chronic kidney disease management in the United Kingdom: NEOERICA project results. *Kidney Int* **72**, 92-99, doi:10.1038/sj.ki.5002273 (2007).

9 Kerr, M., Bray, B., Medcalf, J., O'Donoghue, D. J. & Matthews, B. Estimating the financial cost of chronic kidney disease to the NHS in England. *Nephrol Dial Transplant* **27 Suppl 3**, iii73-80, doi:10.1093/ndt/gfs269 (2012).

10 Magoon, S. *et al.* COVID-19-Related Glomerulopathy: A Report of 2 Cases of Collapsing Focal Segmental Glomerulosclerosis. *Kidney Med* **2**, 488-492, doi:10.1016/j.xkme.2020.05.004 (2020).

11 Sharma, Y. *et al.* COVID-19-Associated Collapsing Focal Segmental Glomerulosclerosis: A Report of 2 Cases. *Kidney Med* **2**, 493-497, doi:10.1016/j.xkme.2020.05.005 (2020).

12 May, R. M. *et al.* A multi-center retrospective cohort study defines the spectrum of kidney pathology in Coronavirus 2019 Disease (COVID-19). *Kidney Int* **100**, 1303-1315, doi:10.1016/j.kint.2021.07.015 (2021).

13 Bhaskaran, K. *et al.* HIV infection and COVID-19 death: a population-based cohort analysis of UK primary care data and linked national death registrations within the OpenSAFELY platform. *The Lancet HIV* **8**, e24-e32, doi:10.1016/s2352-3018(20)30305-2 (2021).

14 Liu, T. *et al.* Twelve-month systemic consequences of COVID-19 in patients discharged from hospital: a prospective cohort study in Wuhan, China. *Clin Infect Dis*, doi:10.1093/cid/ciab703 (2021).

15 Ayoubkhani, D. *et al.* Post-covid syndrome in individuals admitted to hospital with covid-19: retrospective cohort study. *BMJ* **372**, n693, doi:10.1136/bmj.n693 (2021).

16 Bowe, B., Xie, Y., Xu, E. & Al-Aly, Z. Kidney Outcomes in Long COVID. *J Am Soc Nephrol* **32**, 2851-2862, doi:10.1681/ASN.2021060734 (2021).

17 Petersen, E. L. *et al.* Multi-organ assessment in mainly non-hospitalized individuals after SARS-CoV-2 infection: The Hamburg City Health Study COVID programme. *Eur Heart J*, doi:10.1093/eurheartj/ehab914 (2022).

18 Huang, C. *et al.* 6-month consequences of COVID-19 in patients discharged from hospital: a cohort study. *The Lancet* **397**, 220-232, doi:10.1016/s0140-6736(20)32656-8 (2021).

19 Novak, M. *et al*. Increased Risk of Incident Chronic Kidney Disease, Cardiovascular Disease, and Mortality in Patients With Diabetes With Comorbid Depression. *Diabetes Care* **39**, 1940-1947, doi:10.2337/dc16-0048 (2016).

20 Klatte, D. C. F. *et al*. Association Between Proton Pump Inhibitor Use and Risk of Progression of Chronic Kidney Disease. *Gastroenterology* **153**, 702-710, doi:10.1053/j.gastro.2017.05.046 (2017).

21 Janmaat, C. J. *et al*. Lower serum calcium is independently associated with CKD progression. *Sci Rep* **8**, 5148, doi:10.1038/s41598-018-23500-5 (2018).

22 Jansen, J. *et al*. SARS-CoV-2 infects the human kidney and drives fibrosis in kidney organoids. *Cell Stem Cell* **29**, 217-231 e218, doi:10.1016/j.stem.2021.12.010 (2022).

23 Iacobucci, G. Covid-19: What is the UK's testing strategy? *BMJ* **368**, m1222, doi:10.1136/bmj.m1222 (2020).

24 Iwagami, M. *et al*. Seasonality of acute kidney injury incidence and mortality among hospitalized patients. *Nephrol Dial Transplant* **33**, 1354-1362, doi:10.1093/ndt/gfy011 (2018).

25 Goto, S., Hamano, T., Ogata, S. & Masakane, I. Seasonal variations in cause-specific mortality and transition to renal replacement therapy among patients with end-stage renal disease. *Sci Rep* **10**, 2325, doi:10.1038/s41598-020-59153-6 (2020).

26 Grint, D. J. *et al*. Case fatality risk of the SARS-CoV-2 variant of concern B.1.1.7 in England, 16 November to 5 February. *Euro Surveill* **26**, doi:10.2807/1560-7917.ES.2021.26.11.2100256 (2021).

27 Group, R. C. *et al*. Dexamethasone in Hospitalized Patients with Covid-19. *N Engl J Med* **384**, 693-704, doi:10.1056/NEJMoa2021436 (2021).

28 Mansfield, K. E. *et al*. Indirect acute effects of the COVID-19 pandemic on physical and mental health in the UK: a population-based study. *The Lancet Digital Health* **3**, e217-e230, doi:10.1016/s2589-7500(21)00017-0 (2021).

29 Rimmer, A. What has caused the NHS blood tube shortage, and how is it affecting doctors and patients? *BMJ* **374**, n2174, doi:10.1136/bmj.n2174 (2021).

30 Tazare, J. *et al*. Rates of serious clinical outcomes in survivors of hospitalisation with COVID-19: a descriptive cohort study within the OpenSAFELY platform [Pre-print]. *medRxiv*, doi:10.1101/2021.01.22.21250304 (2021).

31 Levey, A. S. *et al*. A new equation to estimate glomerular filtration rate. *Ann Intern Med* **150**, 604-612, doi:10.7326/0003-4819-150-9-200905050-00006 (2009).

32 Gallagher, A. M., Dedman, D., Padmanabhan, S., Leufkens, H. G. M. & de Vries, F. The accuracy of date of death recording in the Clinical Practice Research Datalink

GOLD database in England compared with the Office for National Statistics death registrations. *Pharmacoepidemiol Drug Saf* **28**, 563-569, doi:10.1002/pds.4747 (2019).

33 Mathur, R. *et al.* Ethnic differences in SARS-CoV-2 infection and COVID-19-related hospitalisation, intensive care unit admission, and death in 17 million adults in England: an observational cohort study using the OpenSAFELY platform. *The Lancet* **397**, 1711-1724, doi:10.1016/s0140-6736(21)00634-6 (2021).

34 Bhaskaran, K. *et al.* Overall and cause-specific hospitalisation and death after COVID-19 hospitalisation in England: A cohort study using linked primary care, secondary care, and death registration data in the OpenSAFELY platform. *PLoS Med* **19**, e1003871, doi:10.1371/journal.pmed.1003871 (2022).

35 Office for National Statistics: Smoking prevalence in the UK and the impact of data collection changes: 2020. (2021).

36 Bhaskaran, K., Forbes, H. J., Douglas, I., Leon, D. A. & Smeeth, L. Representativeness and optimal use of body mass index (BMI) in the UK Clinical Practice Research Datalink (CPRD). *BMJ Open* **3**, e003389, doi:10.1136/bmjopen-2013-003389 (2013).

37 Williamson, E. J. *et al.* Factors associated with COVID-19-related death using OpenSAFELY. *Nature* **584**, 430-436, doi:10.1038/s41586-020-2521-4 (2020).

38 Inker, L. A. *et al.* New Creatinine- and Cystatin C-Based Equations to Estimate GFR without Race. *N Engl J Med* **385**, 1737-1749, doi:10.1056/NEJMoa2102953 (2021).

39 Lawlor, D. A., Tilling, K. & Davey Smith, G. Triangulation in aetiological epidemiology. *Int J Epidemiol* **45**, 1866-1886, doi:10.1093/ije/dyw314 (2016).

40 Tennant, P. W. G. *et al.* Use of directed acyclic graphs (DAGs) to identify confounders in applied health research: review and recommendations. *Int J Epidemiol* **50**, 620-632, doi:10.1093/ije/dyaa213 (2021).

41 Griffith, G. J. *et al.* Collider bias undermines our understanding of COVID-19 disease risk and severity. *Nat Commun* **11**, 5749, doi:10.1038/s41467-020-19478-2 (2020).

42 Mahalingasivam, V. *et al.* COVID-19 and kidney disease: insights from epidemiology to inform clinical practice. . *Nature Reviews Nephrology*, doi:https://doi.org/10.1038/s41581-022-00570-3 (2022).

43 Wan, Y. I. *et al.* Acute kidney injury in COVID-19: multicentre prospective analysis of registry data. *Clin Kidney J* **14**, 2356-2364, doi:10.1093/ckj/sfab071 (2021).

Deviations from protocol – Long-term kidney outcomes after COVID-19: a matched cohort study using the OpenSAFELY platform

Section	Original protocol	Deviation	Justification
4.3 Study designs	We will compare people after SARS-CoV-2 infection to both historical and contemporary cohorts in order to address epidemiological challenges to be able to triangulate meaningful conclusions.	The comparison with the historical cohort was designated as a sensitivity analysis.	We had expected the analysis with contemporary comparators may not be robust due to non-ascertainment of asymptomatic/mild COVID-19 infection. However, because of the absence of effect in those with non-hospitalised COVID-19, this did not prove to be an important bias.<br><br>For clarity and avoidance of repetition, we assigned our historical cohort analysis as a sensitivity analysis in our final manuscript.
4.4.2 Historical comparator groups	[We will match historical comparators] 2 years before the first recording of SARS-CoV-2 infection in the exposed case….<br><br>In our secondary analysis, we will compare individuals who remain alive 28 days after admission to hospital with COVID-19 with those who remained alive 28 days after admission to hospital for pneumonia (including influenza) from 1 February 2018 up to two years before the end of follow-up for the exposed group (unmatched)	We matched historical comparators three years before first recording of COVID-19 infection.<br><br>For secondary analysis, we followed-up patients hospitalised for pneumonia from 1 February 2017.	This allowed us to follow-up COVID-19 cases up to 31 January 2023 and historical comparators up to 31 January 2020 (i.e., before the start of COVID-19 epidemic in the UK).

4.4.2 Historical comparator groups & 4.4.3 Contemporary comparator groups	…comparator groups will comprise individuals from the general population under follow-up in OpenSAFELY, each matched five to one to each individual from the recorded SARS-CoV-2 group on age (within 1 year), sex, NHS Sustainability and Transformation Partnership (STP, geographical areas configured for regional reorganisation available in our data), IMD decile (a measure of socioeconomic deprivation based on postcode), and date…	Individuals with COVID-19 were matched to three comparators rather than five.<br><br>Individuals were matched on age, sex, and STP only (i.e., not additionally matched on IMD).	Due to depletion of potential matches, we reduced the number of matches to three.<br><br>We adjusted for IMD (rather than including as an additional matching factor) to maximise the number of potential matches.
4.5.1 Exposure	We will also be able to investigate whether outcomes vary based on infection severity (hospitalisation, critical illness, mechanical ventilation and acute kidney injury [including requiring renal replacement therapy]) as well as calendar time and vaccination status…	We did not use mechanical ventilation or renal replacement therapy as additional levels of exposure.	We decided there would be little added benefit in disaggregating mechanical ventilation from critical care admissions, especially in the context of heightened competing risk of death.<br><br>We did not include acute renal replacement therapy as an additional level of exposure as it was included in our outcome definition (we defined kidney failure as incident dialysis [including any acute renal replacement therapy records recorded between initial COVID-19 record and index date 28 days later on day 1 of follow up], kidney transplantation, or estimated glomerular filtration rate <15ml/min/1.73m^2). However, we investigated the effect of including and excluding acute renal replacement

			therapy (recorded in the 28 days after initial COVID-19 record) from our outcome definition through sensitivity analyses.<br><br>Instead of calendar time and vaccination status being investigated as additional levels of exposure, we fitted these as interaction terms in secondary analysis to be able to determine stratum-specific effects on individuals with and without COVID-19.
4.5.3.3 Covariates (clinical)	We will describe several clinical conditions including: diabetes mellitus, hypertension, ischaemic heart disease, heart failure, atrial fibrillation, stroke, peripheral vascular disease, non-haematological cancer, haematological cancer, chronic respiratory disease, chronic liver disease, dementia, other chronic neurological diseases, systemic lupus erythematosus, vasculitis, rheumatoid arthritis, HIV and sickle cell disease.<br><br>We will also describe albuminuria and/or proteinuria. In addition, we will describe coded CKD as clinical management may vary (e.g. greater routine follow-up) between people diagnosed and subsequently coded	In addition to diabetes mellitus, hypertension and non-haematological cancer, we grouped some covariates into composite categories: 1) cardiovascular disease included ischaemic heart disease, heart failure, atrial fibrillation, stroke and peripheral vascular disease; and 2) immunosuppressive diseases included haematological cancer, HIV, rheumatoid arthritis and systemic lupus erythematosus. We did not include the remaining clinical covariates (i.e., chronic respiratory disease, chronic liver disease, dementia, other chronic neurological diseases, vasculitis, and sickle cell disease).	We grouped covariates to improve model performance. We did excluded some covariates due to relatively low numbers or risk of misclassification.<br><br>We did not extract albuminuria or proteinuria due to underascertainment. We did not extract coded CKD as our models would be adjusting for baseline eGFR instead.[1,2]<br><br>In the OpenSAFELY platform, eFI was only extracted in December 2020. eFI would therefore have been missing for individuals entering the cohort in the earlier months of the COVID-19 pandemic

	as having CKD compared to people without recognised CKD.<br><br>…we will request baseline electronic frailty index (eFI) calculations for each individual aged ≥65 years…<br><br>We will describe the number of individuals who were being prescribed drugs from the following classes in primary care: insulin, other antidiabetic drugs, immunosuppressants and anticoagulants.	We did not extract albuminuria or proteinuria. We did not extract coded CKD.<br><br>We did not extract eFI.<br><br>We did not extract prescription data.	(i.e., March 2020 to December 2020) and at more likely to be misclassified for those entering the cohort later in the study period (as the eFI was not updated after December 2020, so would not have captured deficits occurring between then and a subsequent cohort entry).<br><br>We did not extract prescription data as we were adjusting for the conditions they were treating. Immunosuppressants will often be unascertained in primary care records due to secondary care prescribing.
4.6.1 Main analyses	Due to the competing risk of death, we will use Fine and Gray regression models (using calendar time as the underlying timescale) to estimate subdistribution hazard ratios…<br><br>We will run sequential regression models:<br>1. Minimally adjusted for age (using spline functions) and sex,<br>2. Additionally adjusting for ethnicity, IMD, smoking and BMI,<br>3. Additionally adjusting for clinical comorbidities.	We used Cox models stratified by matched set to obtain hazard ratios, overall, and stratified by time period instead of Fine and Grey models.<br><br>We ran fully-adjusted models only.<br><br>We undertook complete case analysis without making assumptions about individuals with missing BMI or smoking status data.	Cox models quantify average cause-specific hazards over the course of follow-up, as well as more meaningful period-specific hazards. These are more informative for policymaking than subdistribution hazards.<br><br>We ran only fully-adjusted models to be able to present the most meaningful estimates only after adjustment for potential confounding variables.

	We will assume people with missing BMI are not obese and people without a documented smoking status are non-smokers,		We performed complete case analysis to minimize effects of misclassification.
4.6.2 Secondary analyses	*A priori,* we intend to fit interaction terms to obtain hazard ratio estimates stratified by age group, ethnicity, quintile of IMD, frailty group (for those aged 65 years and over only), diabetes, baseline eGFR, coded CKD, and calendar time of infection.<br><br>Stratified SHRs will also be reported for critical care admission, mechanical ventilation, acute RRT and AKI after COVID-19.<br><br>We will describe Kaplan-Meier graphs for survival after COVID-19. We will obtain *p*-values using log rank tests.	We fitted interaction terms for age, sex, ethnicity, diabetes, baseline eGFR, vaccination status and COVID-19 wave (i.e. calendar time of infection).<br><br>We obtained stratum-specific hazard ratios overall, and by hospitalization status only (i.e. hospitalised and non-hospitalised).<br><br>We did not produce Kaplan-Meier graphs.	We felt sex was an important potential effect modifier. We felt interaction for IMD would not provide important additional information.<br><br>We did not investigate interaction by frailty or coded CKD status: 1) We were unable to capture reliable time-updated measures of frailty; and 2) evidence indicates that coded CKD status does not capture CKD status as reliably as CKD stage established using serum creatinine test results to calculate eGFR measures.[2]<br><br>Investigating interactions by critical care admission or acute kidney injury would have been difficult to interpret due to the high competing risk of death with our outcomes.<br><br>We did not have access to the data after June 30 2024 to be able to obtain standardized incidence curves as planned. We undertook assessment for

			proportional hazards using Schoenfeld residuals and presented period-specific hazard ratios.
4.6.3 Sensitivity analyses		Our historical cohort analysis was presented as a sensitivity analysis.	For clarity and avoidance of repetition, we assigned our historical cohort analysis as a sensitivity analysis in our final manuscript.
4.6.3.2 Sensitivity analyses (eGFR reduction)	In case a difference in 50% reduction in eGFR is not identified, we will also investigate for 40% and 30% reduction in eGFR.	We did not investigate for 40% and 30% reduction in eGFR.	We identified an important effect on 50% reduction in eGFR and so these additional sensitivity analyses were not required.
4.6.3.3 Sensitivity analyses (incident eGFR <30ml/min/1.73m^2)	As healthcare services have been impacted heavily by the pandemic, there may not have been sufficient routine testing of patients to be able to detect a reduction in eGFR. As patients who are highest risk may be prioritised, we will also investigate for incident eGFR <30ml/min/1.73m^2…	We did not investigate for incident eGFR <30ml/min/1.73m^2.	We identified an important effect on 50% reduction in eGFR and so this additional sensitivity analysis was not required.
4.6.3.4 Sensitivity analyses (eGFR equation)	We will assess how many individuals change CKD stage from the original CKD-EPI equation after switching to the 2021 equation. If there are marked differences in the number of people with CKD and within each CKD stage, we will undertake a	We did not undertake an analysis using the 2021 CKD-EPI eGFR equation.	This equation has not yet been validated for use in clinical practice in the UK.

	sensitivity analysis using the 2021 equation instead for eGFR.		
4.6.3.5 Sensitivity analyses (Age)	As our main analyses will adjust for age in broad age bands, to ensure that we have fully accounted for age as a potential confounder, we will repeat analyses with age as the underlying timescale (allowing us to finely account for age).	We did not undertake this sensitivity analysis.	Our main analysis implicitly adjusted for exact age as our models were stratified by matched set (with age as one of the matching variables).
4.6.3.7 Sensitivity analyses (Smoking)	In our main analyses we will assume that individuals with missing smoking status were non-smokers. To test this assumption, we will repeat our analyses restricting to those with known smoking status.	In our main analyses, we undertook complete case analysis without making assumptions about individuals with missing smoking status data.	We decided to perform complete case analysis (i.e. the planned sensitivity analysis) to minimize effects of misclassification.
4.6.3.8 Sensitivity analyses (BMI)	In our main analyses we will assume that individuals with missing BMI data have normal BMI. To test this assumption, we will repeat our analyses restricting to those with known BMI.	In our main analyses, we undertook complete case analysis without making assumptions about individuals with missing BMI status data.	We decided to perform complete case analysis (i.e. the planned sensitivity analysis) to minimize effects of misclassification.
