# Supplementary material for: Long-term kidney outcomes after COVID-19: a matched cohort study using the OpenSAFELY platform
Source: Lancet Reg Health Eur. 2025 Jun 18;55:101338. doi: 10.1016/j.lanepe.2025.101338 (PMC12426834; doi:10.1016/j.lanepe.2025.101338)

Supplementary Material: Long-term kidney outcomes after COVID-19: a matched cohort study using the OpenSAFELY platform

Viyaasan Mahalingasivam, MPhil [a,b] · Bang Zheng, PhD [a] · Kevin Wing, PhD [c] · Edward P K Parker, PhD [d] · Krishnan Bhaskaran, PhD [a] · Juan Jesús Carrero, PhD [e] · Sandra Jayacodi [f] · Edith Jumbo [f] · Tamanna Miah, BA [f] · Brian Gracey, MA [f] · John Tazare, PhD [g] · Shalini Santhakumaran, PhD [h] · Rohini Mathur, PhD [i] · Ruth E Costello, PhD [a] · Emily Herrett, PhD [a] · Qing Wen, PhD [a] · Thomas Hartney, MSc [a] · Ian J Douglas, PhD [a] · Amelia Green, PhD [j] · Louis Fisher, MSc [j] · Helen J Curtis, DPhil [j] · Alex J Walker, PhD [j] · Brian MacKenna, MPharm [j] · William J Hulme, PhD [j] · Amir Mehrkar, MRCGP [j] · Sebastian Bacon, BA [j] · Ben Goldacre, MRCPsych [j] · Elizabeth Williamson, PhD [g] · Dorothea Nitsch, MD [a,h] · Kathryn E Mansfield, PhD [k] · Laurie Tomlinson, PhD [a] for the OpenSAFELY Collaborative

a. Department of Non-Communicable Disease Epidemiology, London School of Hygiene & Tropical Medicine, London, UK

b. Department of Nephrology & Transplantation, Barts Health NHS Trust, London, UK

c. School of Health & Wellbeing, University of Glasgow, Glasgow, UK

d. NIHR Health Protection Research Unit in Vaccines and Immunisation, London School of Hygiene & Tropical Medicine, London, UK

e. Department of Medical Epidemiology & Biostatistics, Karolinska Institutet, Solna, Sweden

f. Patient and public involvement partner, UK

g. Department of Medical Statistics, London School of Hygiene & Tropical Medicine, London, UK

h. UK Kidney Association, Bristol, UK

i. Wolfson Institute of Population Health, Queen Mary University of London, London, UK

j. Bennett Institute for Applied Data Science, University of Oxford, Oxford, UK

k. School of Health and Care Sciences, University of Lincoln, Lincoln, UK

Table of contents

Figure S1 ... 3
Figure S2 ... 4
Figure S3 ... 5
Figure S4 ... 7
Figure S5 ... 8
Figure S6 ... 10
Figure S7 ... 11
Figure S8 ... 12
Figure S9 ... 13
Figure S10 ... 14
Table S1 ... 15
Table S2 ... 17
Table S3 ... 20
Table S4 ... 23
Table S5 ... 26
Table S6 ... 27
Table S7 ... 30
Table S8 ... 33
Table S9 ... 35
Table S10 ... 37
Table S11 ... 40
Table S12 ... 42
Table S13 ... 43
Table S14 ... 44
Table S15 ... 45
Table S16 ... 48
Appendix S1 ... 49
Appendix S2 ... 50
Appendix S3 ... 51
Appendix S4 ... 52
Appendix S5 ... 54
Appendix S6 ... 55

Figure S1

Study population flowchart for matched historical sensitivity analysis.

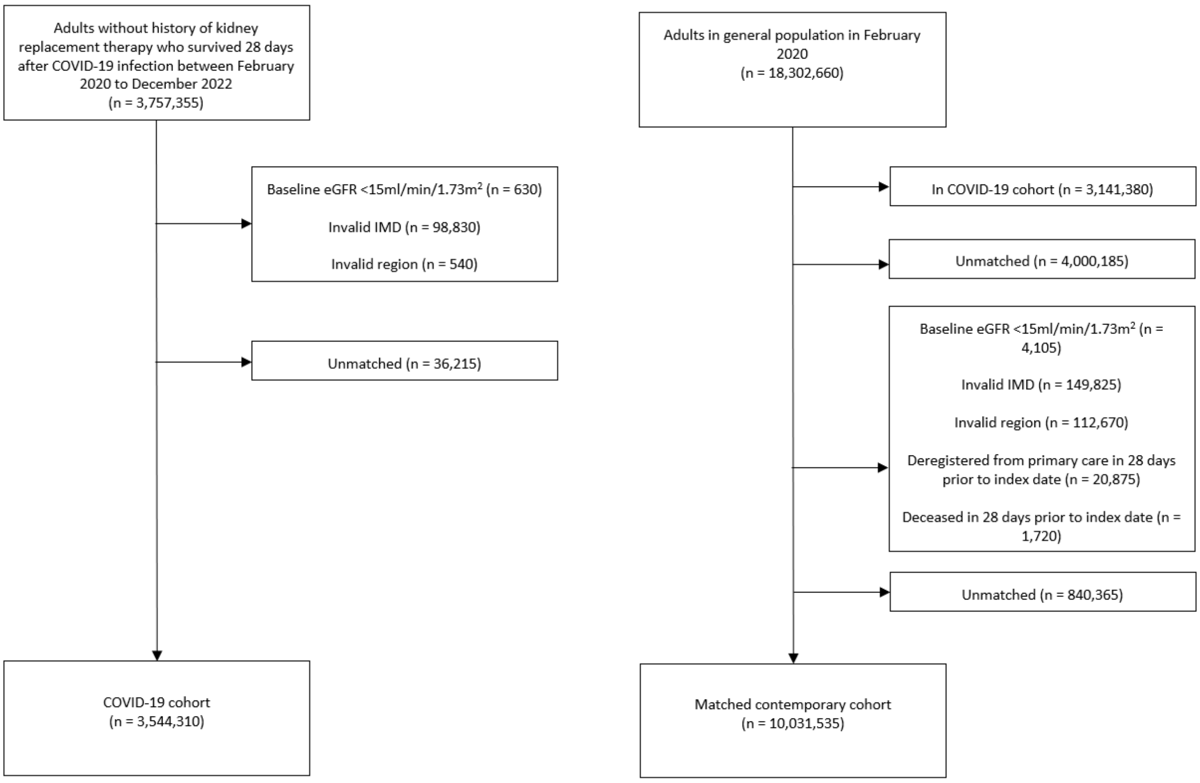

Counts rounded to nearest 5. eGFR = estimated glomerular filtration rate, IMD = index of multiple deprivation.

Initial extractions from OpenSAFELY platform excluded individuals without valid age, sex, region and IMD.

Figure S2

Fully-adjusted hazard ratio and adjusted rate difference estimates for kidney failure (i.e., incident dialysis, kidney transplantation, or estimated glomerular filtration rate (eGFR) <15 ml/min/1.73m^2), 50% reduction in eGFR, and death after COVID-19 in a **sensitivity analysis** compared to an age-, sex- and sustainability and transformation partnership region-matched **historical cohort**, overall and by COVID-19 hospitalisation status, by specific follow-up periods (in days since index date, i.e., 28 days after first COVID-19 infection record) (**Table S7**).

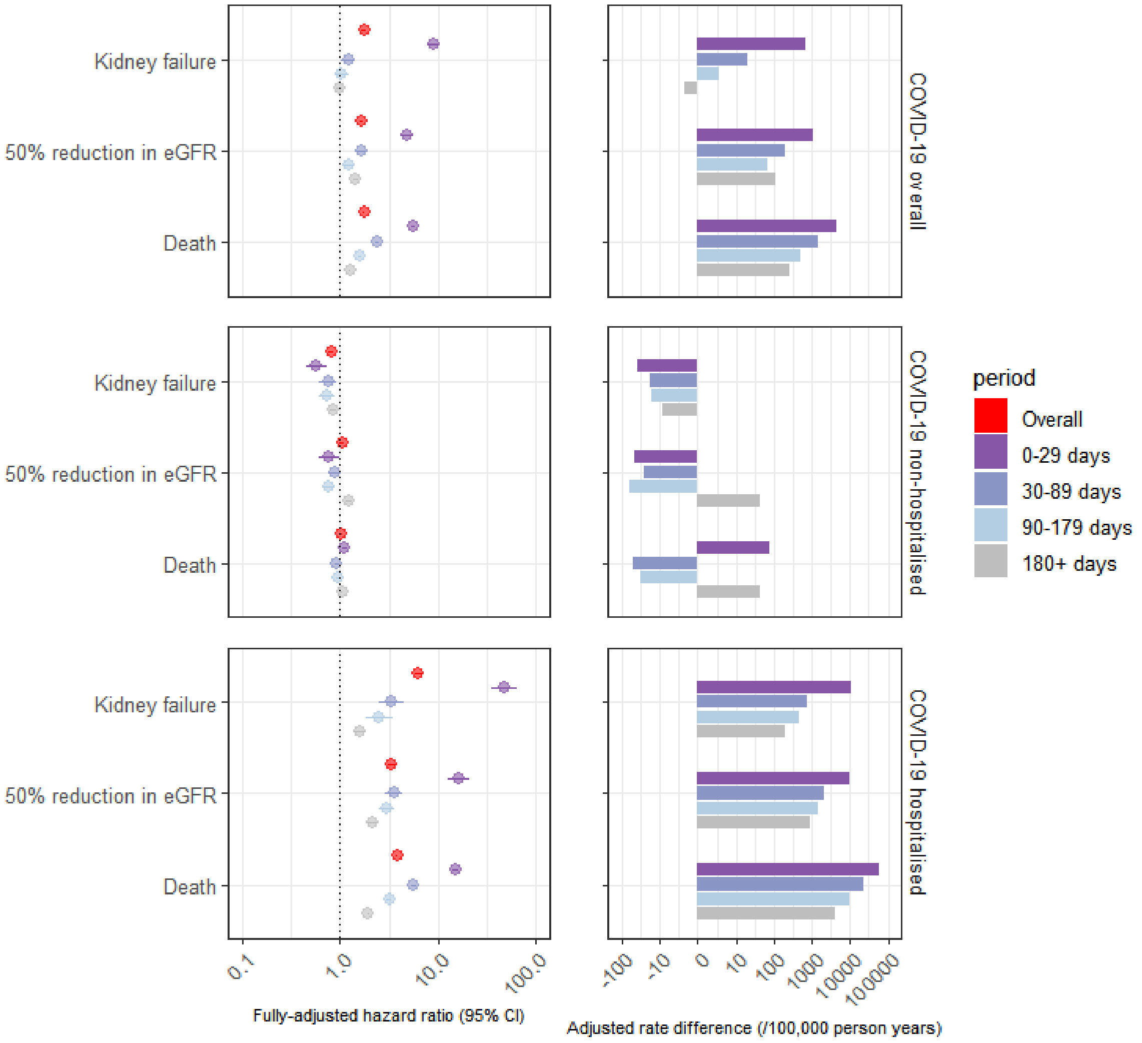

Models adjusted for ethnicity, deprivation, rural or urban, body mass index, smoking status, baseline eGFR (with "no baseline eGFR measurement" treated as a categorical variable), previous acute kidney injury, cardiovascular diseases, diabetes mellitus, hypertension, immunosuppressive diseases, non-haematological cancer, general practice consultations in the previous year and hospital admissions in the previous five years. Fully-adjusted hazard ratio and adjusted rate difference on log scale. CI = confidence interval.

Figure S3

Fully-adjusted hazard ratio estimates for kidney failure (i.e., incident dialysis, kidney transplantation or estimated glomerular filtration rate (eGFR) <15 ml/min/1.73m^2) (main analysis), and a series of **additional sensitivity analyses** compared to an age-, sex- and sustainability and transformation partnership region-matched cohort.

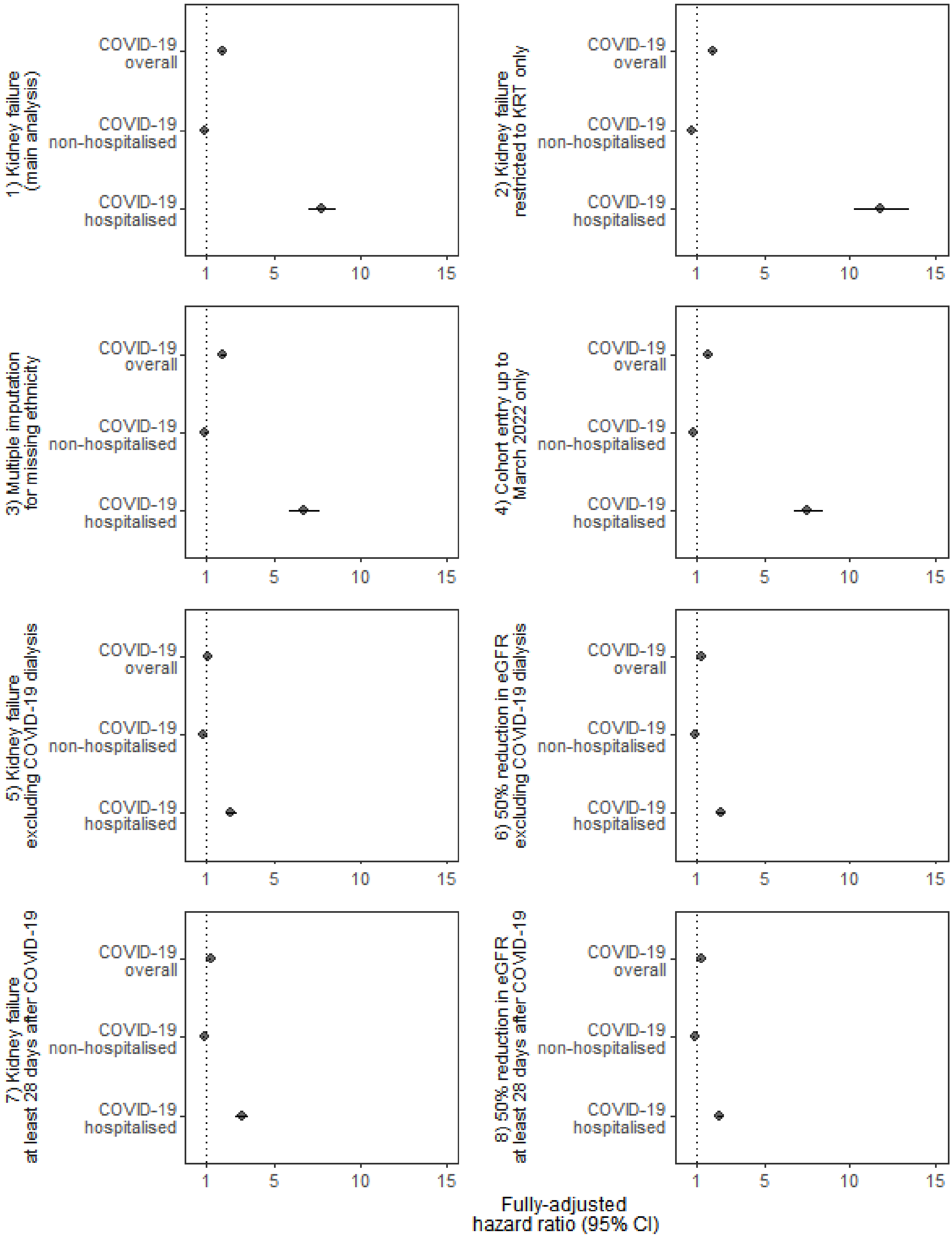

1) Kidney failure (main analysis); 2) Kidney failure redefined to include only incident kidney replacement therapy (KRT) (i.e., not including individuals with incident eGFR <15ml/min/1.73m^2 without a code for dialysis or kidney transplantation); 3) Kidney failure after multiple imputation for missing ethnicity data; 4) COVID-19 cases restricted up to March 2022 (i.e., the end of universal access to COVID-19 testing); 5) Kidney failure excluding individuals requiring dialysis within 28 days of COVID-19 diagnosis; 6) 50% reduction in eGFR excluding individuals requiring dialysis within 28 days of COVID-19 diagnosis; 7) kidney failure at least 28 days after COVID-19 diagnosis; 8) 50% reduction in eGFR at least 28 days after COVID-19 diagnosis (**Table S1**). Models adjusted for ethnicity, deprivation, rural or urban, body mass index, smoking status, baseline eGFR (with "no baseline eGFR measurement" treated as a categorical variable), previous acute kidney injury, cardiovascular diseases, diabetes mellitus, hypertension, immunosuppressive diseases, non-haematological cancer, general practice consultations in the previous year hospital admissions in the previous five years, and COVID-19 vaccination. CI = confidence interval.

Figure S4

Fully-adjusted hazard ratio estimates for kidney failure (i.e., incident dialysis, kidney transplantation or estimated glomerular filtration rate (eGFR) <15 ml/min/1.73m^2), stratified by potential effect modifiers, for COVID-19 compared to an age-, sex-, and sustainability and transformation partnership region-matched **historical cohort** (**Table S9**).

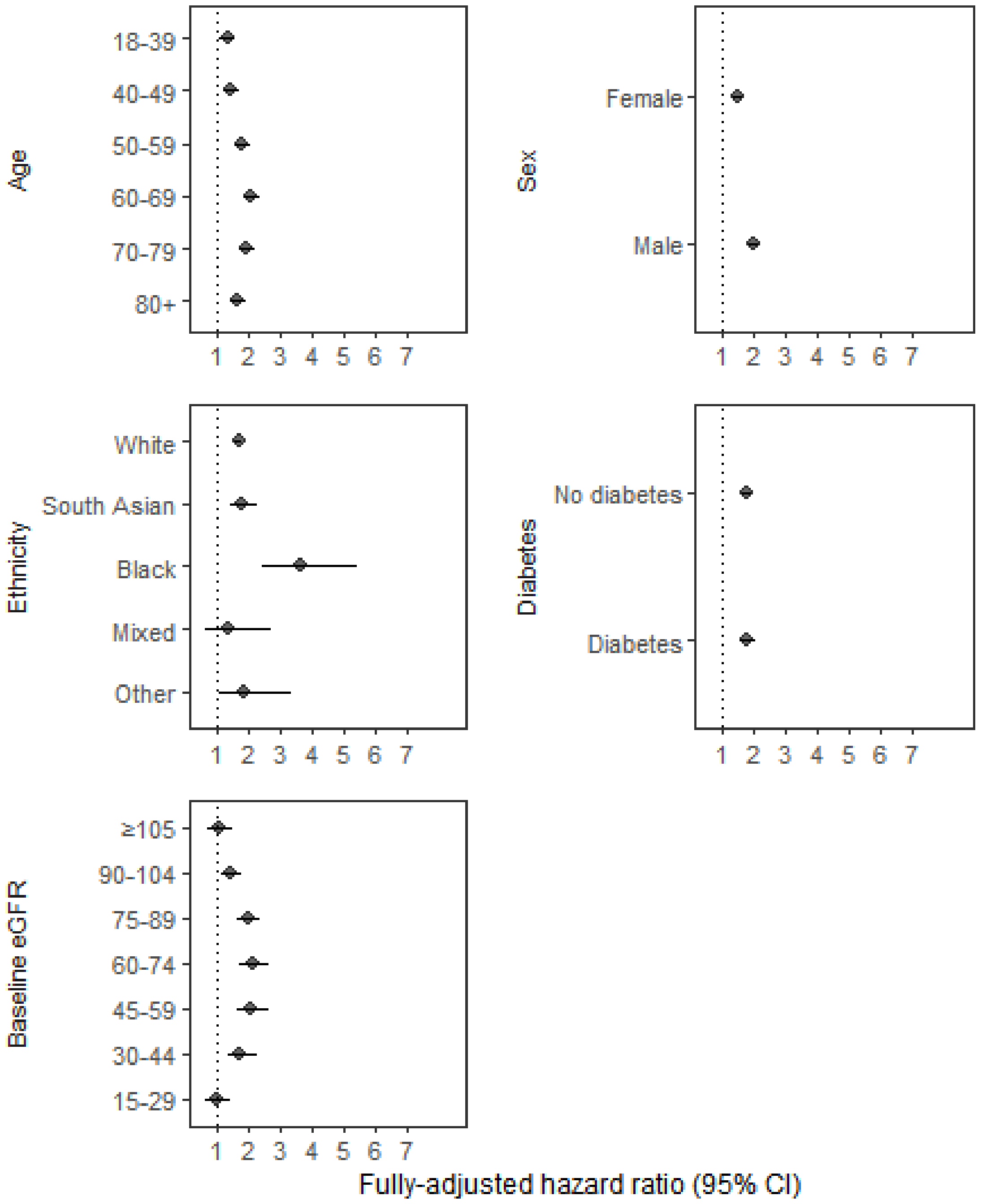

Models adjusted for ethnicity, deprivation, rural or urban, body mass index, smoking status, baseline eGFR (with "no baseline eGFR measurement" treated as a categorical variable), previous acute kidney injury, cardiovascular diseases, diabetes mellitus, hypertension, immunosuppressive diseases, non-haematological cancer, general practice consultations in the previous year, and hospital admissions in the previous five years. Age in years. Baseline eGFR in ml/min/1.73m^2. CI = confidence interval.

Figure S5

Fully-adjusted hazard ratio estimates for kidney failure (i.e., incident dialysis, kidney transplantation or estimated glomerular filtration rate (eGFR) <15 ml/min/1.73m^2), **stratified by potential effect modifiers**, for COVID-19 **stratified by hospitalisation status** compared to an age-, sex- and sustainability and transformation partnership region-matched cohort (**Table S10**).

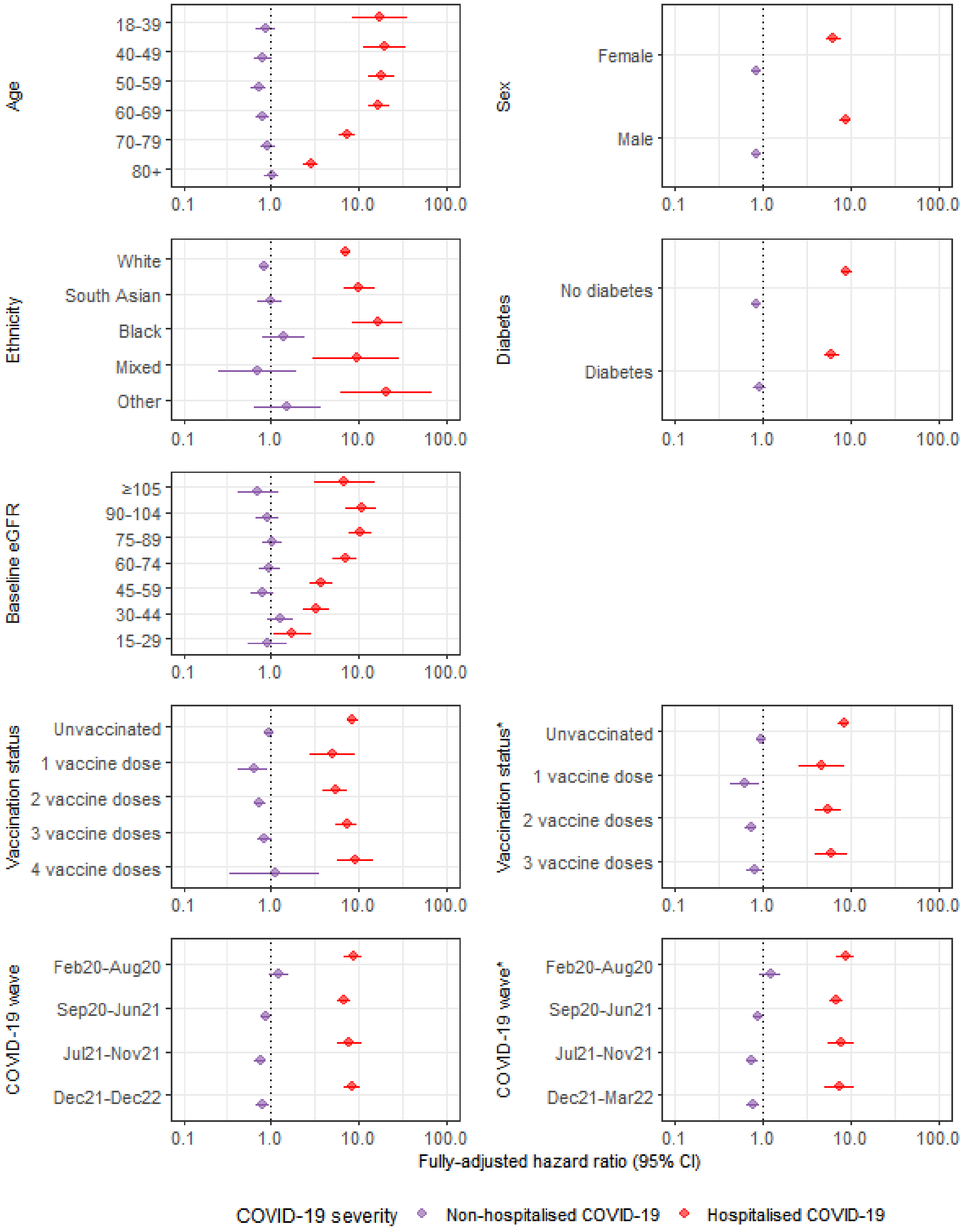

Models adjusted for ethnicity, deprivation, rural or urban, body mass index, smoking status, baseline eGFR (with "no baseline eGFR measurement" treated as a categorical variable), previous acute kidney injury, cardiovascular diseases, diabetes mellitus, hypertension, immunosuppressive diseases, non-haematological cancer, general practice consultations in the previous year, hospital admissions in the previous five years, and COVID-19 vaccination status and COVID-19 wave. Fully-adjusted hazard ratio on log scale. Age in years. Baseline eGFR in ml/min/1.73m^2. CI = confidence interval. *COVID-19 vaccination status and COVID-19 wave restricted to cases up to March 2022 (i.e., the end of universal access to COVID-19 testing).

Figure S6

Fully-adjusted hazard ratio estimates for kidney failure (i.e., incident dialysis, kidney transplantation or estimated glomerular filtration rate (eGFR) <15 ml/min/1.73m^2), stratified by **potential effect modifiers**, for COVID-19 **stratified by hospitalisation status** compared to an age-, sex- and sustainability and transformation partnership region-matched **historical** cohort (**Table S11**).

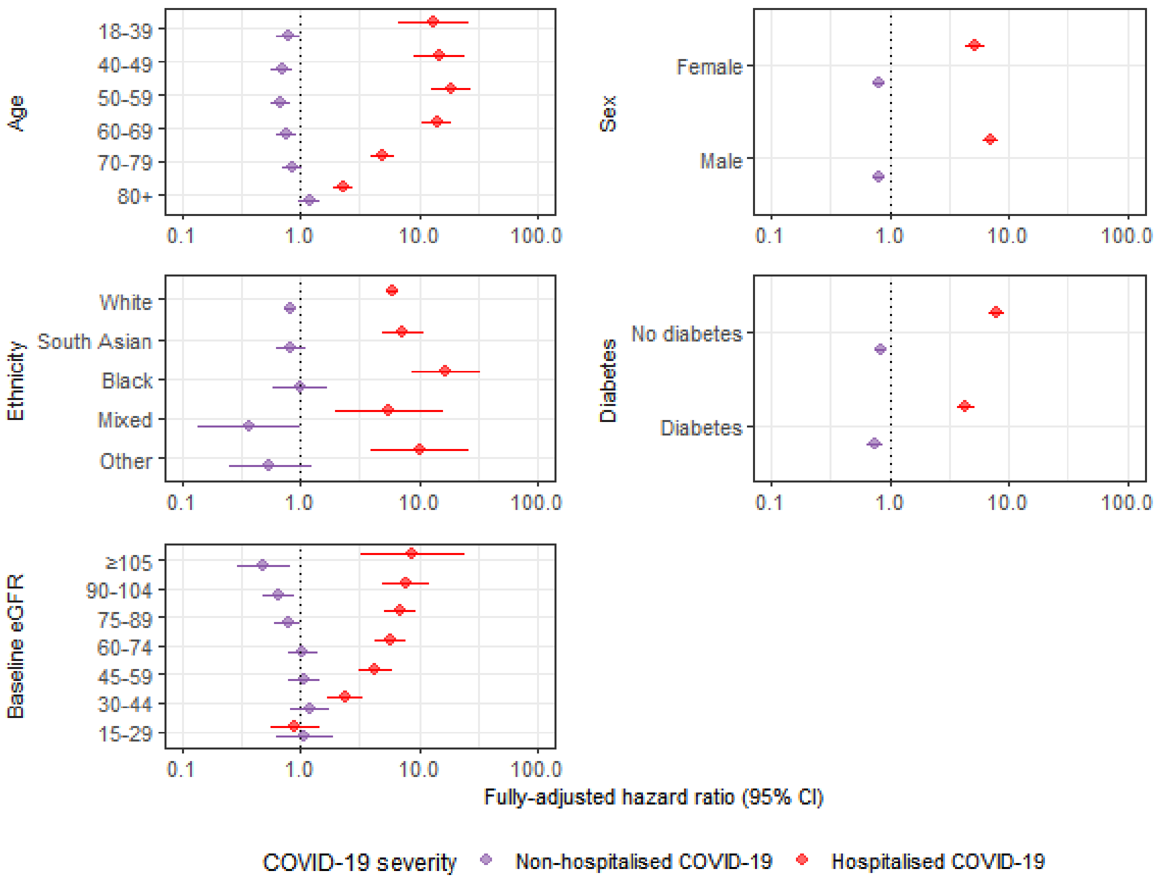

Models adjusted for ethnicity, deprivation, rural or urban, body mass index, smoking status, baseline eGFR (with "no baseline eGFR measurement" treated as a categorical variable), previous acute kidney injury, cardiovascular diseases, diabetes mellitus, hypertension, immunosuppressive diseases, non-haematological cancer, general practice consultations in the previous year and hospital admissions in the previous five years. Fully-adjusted hazard ratio on log scale. Age in years. Baseline eGFR in ml/min/1.73m^2. CI = confidence interval.

Figure S7

Fully-adjusted hazard ratio estimates for kidney failure (i.e., incident dialysis, kidney transplantation or estimated glomerular filtration rate (eGFR) <15 ml/min/1.73m^2), 50% reduction in eGFR, and death after COVID-19 **overall** (left panel) and stratified by **hospitalisation status** (right panel) compared to an age-, sex- and sustainability and transformation partnership region-matched cohort, **stratified by COVID-19 wave** as an interaction (**Table S12; Table S13**).

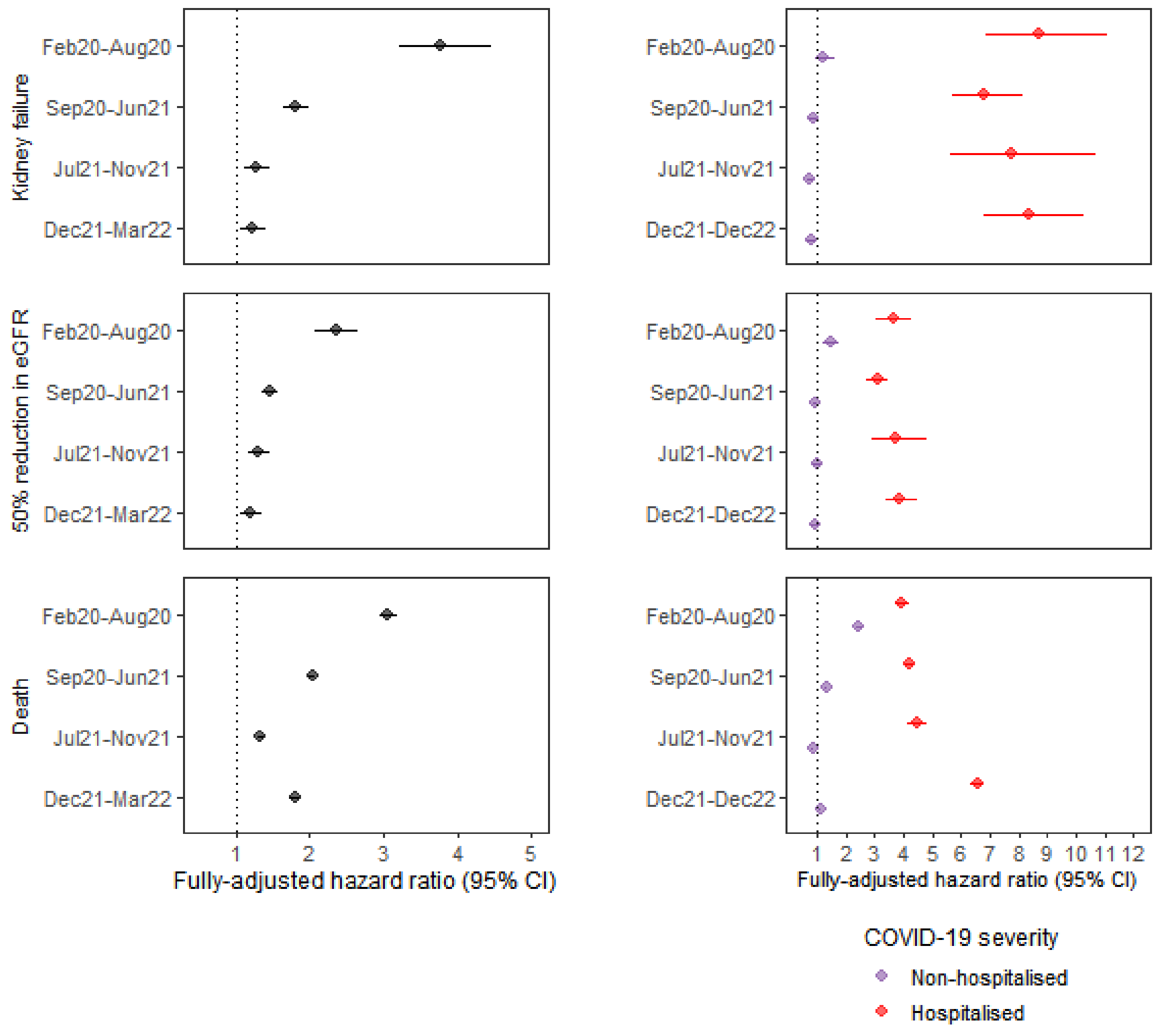

Models adjusted for ethnicity, deprivation, rural or urban, body mass index, smoking status, baseline eGFR (with "no baseline eGFR measurement" treated as a categorical variable), previous acute kidney injury, cardiovascular diseases, diabetes mellitus, hypertension, immunosuppressive diseases, non-haematological cancer, general practice consultations in the previous year, hospital admissions in the previous five years and COVID-19 vaccination status. For COVID-19 overall (left column), wave 4 is restricted to December 2021 to March 2022 (i.e. the end of universal access to COVID-19 testing). CI = confidence interval.

Figure S8

Fully-adjusted hazard ratio estimates for kidney failure (i.e., incident dialysis, kidney transplantation or estimated glomerular filtration rate (eGFR) <15 ml/min/1.73m^2) after COVID-19 stratified by **levels of COVID-19 severity** compared to an age-, sex- and sustainability and transformation partnership region-matched **cohort** (**Table S14**).

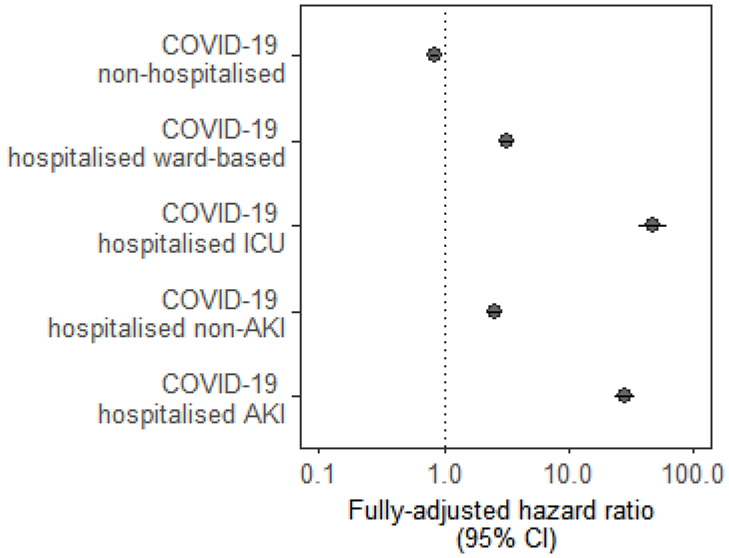

Models adjusted for ethnicity, deprivation, rural or urban, body mass index, smoking status, baseline eGFR (with "no baseline eGFR measurement" treated as a categorical variable), previous acute kidney injury, cardiovascular diseases, diabetes mellitus, hypertension, immunosuppressive diseases, non-haematological cancer, general practice consultations in the previous year, hospital admissions in the previous five years, and COVID-19 vaccination status. Fully-adjusted hazard ratio on log scale. AKI = acute kidney injury, CI = confidence interval, ICU = intensive care unit.

Figure S9

Study population flowchart for analysis comparing hospitalised COVID-19 with hospitalised pneumonia; counts rounded to nearest 5. IMD = index of multiple deprivation

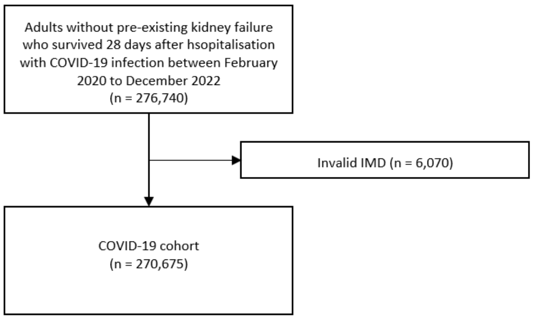

Figure S10

Fully-adjusted hazard ratio estimates for kidney failure (i.e., incident dialysis, kidney transplantation or estimated glomerular filtration rate (eGFR) <15 ml/min/1.73m^2), 50% reduction in eGFR, and death **after COVID-19 hospitalisation** compared to a **historical** cohort after **hospitalisation for pneumonia**, over all time periods and by specific follow-up periods (in days since index date, i.e., 28 days after first COVID-19 infection record) (**Table S14**).

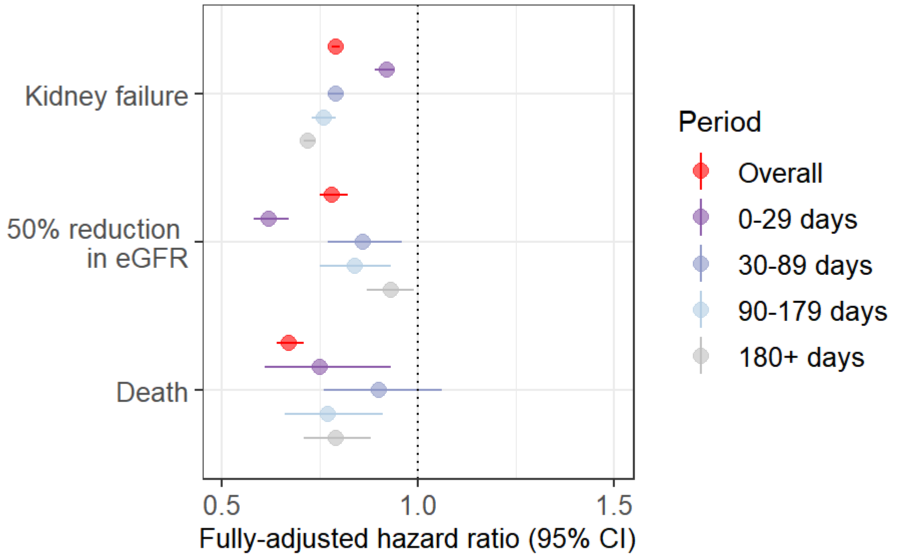

Models adjusted for ethnicity, deprivation, rural or urban, body mass index, smoking status, baseline eGFR, previous acute kidney injury, cardiovascular diseases, diabetes mellitus, hypertension, immunosuppressive diseases, non-haematological cancer, general practice consultations in the previous year, hospital admissions in the previous five years and calendar month. CI = confidence interval.

Table S1

Description and justification for a series of additional sensitivity analyses with corresponding fully-adjusted hazard ratio estimates for COVID-19 compared to an age-, sex- and sustainability and transformation partnership region-matched cohort.

Description	Justification	Fully-adjusted hazard ratio (95% CI)		
		COVID-19 overall	COVID-19 non-hospitalised	COVID-19 hospitalised
Main analysis	Included for comparison	1.93 (1.84-2.03)	0.85 (0.79-0.90)	7.74 (7.00-8.56)
Kidney failure outcome defined as incident kidney replacement therapy only	To determine the burden of kidney replacement therapy outcomes, we redefined kidney failure as an outcome to include only coded dialysis or kidney transplantation (i.e., not including eGFR <15 ml/min/1.73m^2).	1.99 (1.88-2.10)	0.74 (0.69-0.80)	11.73 (10.23-13.46)
Kidney failure with multiple imputation for missing ethnicity*	To address bias due to missing ethnicity data, we replicated our main analysis for kidney failure after using multiple imputation for missing ethnicity.	1.97 (1.84-2.10)	0.91 (0.84-1.00)	6.60 (5.76-7.57)
Kidney failure with COVID-19 cases restricted up to March 2022	To address bias in COVID-19 coding due to the end of universal access to testing, we replicated our main analysis for kidney failure, restricting COVID-19 cases to those diagnosed up to March 2022 only.	1.72 (1.63-1.82)	0.83 (0.78-0.89)	7.52 (6.72-8.42)
Kidney failure excluding patients with hospital dialysis at time of COVID-19	To distinguish the influence of severe AKI during COVID-19 illness on outcome estimates, we replicated our main analyses for kidney failure after excluding individuals first coded as requiring kidney replacement therapy within 28 days of COVID-19	1.10 (1.04-1.17)	0.83 (0.78-0.89)	2.43 (2.16-2.74)
50% reduction in eGFR excluding patients with hospital dialysis at time of COVID-19	To distinguish the influence of severe AKI during COVID-19 illness on outcome estimates, we replicated our main analyses for 50% reduction in eGFR after excluding individuals first coded as requiring kidney replacement therapy within 28 days of COVID-19	1.36 (1.31-1.42)	0.99 (0.94-1.04)	2.46 (2.28-2.64)
Kidney failure at least 28 days after COVID-19	To distinguish the influence of severe AKI during COVID-19 illness on outcome estimates, we replicated our main analyses for kidney failure only including codes for kidney replacement therapy from 28 days after COVID-19 diagnosis (i.e., implying ongoing kidney failure beyond the initial illness).	1.22 (1.15-1.29)	0.84 (0.79-0.90)	3.06 (2.73-3.43)

50% reduction in eGFR at least 28 days after COVID-19	To distinguish the influence of severe AKI during COVID-19 illness on outcome estimates, we replicated our main analyses for 50% reduction in eGFR only including codes for kidney replacement therapy from 28 days after COVID-19 diagnosis (i.e., implying ongoing kidney failure beyond the initial illness).	1.29 (1.24-1.34)	0.90 (0.86-0.95)	2.40 (2.24-2.58)

Models adjusted for ethnicity, deprivation, rural or urban, body mass index, smoking status, baseline eGFR (with “no baseline eGFR measurement” treated as a categorical variable), previous AKI, cardiovascular diseases, diabetes mellitus, hypertension, immunosuppressive diseases, non-haematological cancer, general practice consultations in the previous year and hospital admissions in the previous five years. (**Figure S3**). Event counts rounded to nearest 5. AKI = acute kidney injury, CI = confidence interval, eGFR = estimated glomerular filtration rate.

*Multinomial logistic regression was used as the imputation model including all other covariates and kidney failure (as a binary outcome variable), with ten imputed datasets created. Hazard ratios were combined using Rubin’s rules.

Table S2

Demographic, socioeconomic and clinical characteristics for COVID-19 cohorts, stratified by hospitalisation status, and an age-, sex- and sustainability and transformation partnership region-matched contemporary cohort.

	COVID-19 vs matched contemporary population (main analysis)			COVID-19 vs matched historical population (sensitivity analysis)		
	COVID-19 non-hospitalised	COVID-19 hospitalised	Matched cohort	COVID-19 non-hospitalised	COVID-19 hospitalised	Matched cohort
Number of individuals	3300465	243845	10031535	3373120	248020	10586610
Follow-up (days), median (IQR)	449 (371-699)	354 (145-708)	410 (361-567)	444 (370-694)	349 (146-705)	444 (369-701)
Age (years), median (IQR)	43 (32-55)	71 (55-82)	44 (32-57)	42 (31-54)	71 (54-82)	44 (32-56)
Sex, n (%)						
Female	1766675 (53.5)	119665 (49.1)	5306755 (52.9)	1837140 (54.5)	123725 (49.9)	5723975 (54.1)
Male	1533790 (46.5)	124180 (50.9)	4724780 (47.1)	1535980 (45.5)	124295 (50.1)	4862635 (45.9)
Index of multiple deprivation, n (%)						
1 Most deprived	667035 (20.2)	59730 (24.5)	2104640 (21.0)	688280 (20.4)	61135 (24.6)	2204035 (20.8)
2	668005 (20.2)	50945 (20.9)	2004605 (20.0)	683005 (20.2)	51805 (20.9)	2112930 (20.0)
3	696160 (21.1)	50560 (20.7)	2104315 (21.0)	708875 (21.0)	51260 (20.7)	2220980 (21.0)
4	658475 (20.0)	44510 (18.3)	1981500 (19.8)	670705 (19.9)	45205 (18.2)	2095920 (19.8)
5 Least deprived	610795 (18.5)	38095 (15.6)	1836475 (18.3)	622255 (18.4)	38615 (15.6)	1952740 (18.4)
Ethnicity, n (%)						
White	2388985 (72.4)	179185 (73.5)	7085150 (70.6)	2440160 (72.3)	182055 (73.4)	7591135 (71.7)
South Asian	224930 (6.8)	16080 (6.6)	680615 (6.8)	229435 (6.8)	16495 (6.7)	681460 (6.4)
Black	68985 (2.1)	5640 (2.3)	233515 (2.3)	70155 (2.1)	5745 (2.3)	225075 (2.1)
Mixed	39470 (1.2)	2015 (0.8)	122310 (1.2)	40415 (1.2)	2085 (0.8)	115555 (1.1)
Other	47535 (1.4)	3180 (1.3)	237785 (2.4)	48490 (1.4)	3260 (1.3)	202990 (1.9)
Missing	530560 (16.1)	37745 (15.5)	1672165 (16.7)	544470 (16.1)	38380 (15.5)	1770395 (16.7)
Region, n (%)						
East Midlands	602620 (18.3)	46210 (19.0)	1844310 (18.4)	617465 (18.3)	47480 (19.1)	1960365 (18.5)
East	741770 (22.5)	51225 (21.0)	2240430 (22.3)	752210 (22.3)	51945 (20.9)	2357650 (22.3)

London	205110 (6.2)	12805 (5.3)	618245 (6.2)	205305 (6.1)	12820 (5.2)	633970 (6.0)
North East	178740 (5.4)	15125 (6.2)	552690 (5.5)	185895 (5.5)	15410 (6.2)	592770 (5.6)
North West	327635 (9.9)	24870 (10.2)	995750 (9.9)	343785 (10.2)	25375 (10.2)	1079210 (10.2)
South East	194785 (5.9)	15000 (6.2)	595370 (5.9)	195565 (5.8)	15080 (6.1)	614510 (5.8)
South West	401050 (12.2)	29730 (12.2)	1224560 (12.2)	402370 (11.9)	29840 (12.0)	1267650 (12.0)
West Midlands	143315 (4.3)	13165 (5.4)	448735 (4.5)	147105 (4.4)	13475 (5.4)	473890 (4.5)
Yorkshire and The Humber	505440 (15.3)	35710 (14.6)	1511450 (15.1)	523420 (15.5)	36600 (14.8)	1606600 (15.2)
Urban/rural, n (%)						
Urban	2694575 (81.6)	198020 (81.2)	8043555 (80.2)	2756400 (81.7)	201625 (81.3)	8497210 (80.3)
Rural	605890 (18.4)	45825 (18.8)	1987985 (19.8)	616720 (18.3)	46395 (18.7)	2089400 (19.7)
Body mass index, n (%)						
<18.5 kg/m^2	51815 (1.6)	6695 (2.7)	205770 (2.1)	53835 (1.6)	6875 (2.8)	209820 (2.0)
18.5-24.9 kg/m^2	975475 (29.6)	64235 (26.3)	3155620 (31.5)	1000415 (29.7)	65555 (26.4)	3386070 (32.0)
25.0-29.9 kg/m^2	949135 (28.8)	74300 (30.5)	2750620 (27.4)	966065 (28.6)	75280 (30.4)	2955225 (27.9)
30.0-34.9 kg/m^2	493760 (15.0)	47565 (19.5)	1365695 (13.6)	503420 (14.9)	48235 (19.4)	1443525 (13.6)
35.0-39.9 kg/m^2	202785 (6.1)	21930 (9.0)	548850 (5.5)	207720 (6.2)	22270 (9.0)	567495 (5.4)
≥40.0 kg/m^2	107310 (3.3)	13355 (5.5)	294820 (2.9)	110650 (3.3)	13615 (5.5)	292780 (2.8)
Missing	520185 (15.8)	15760 (6.5)	1710165 (17.0)	531015 (15.7)	16195 (6.5)	1731705 (16.4)
Smoking, n (%)						
Non-smoker	1598300 (48.4)	90790 (37.2)	4692210 (46.8)	1636785 (48.5)	92685 (37.4)	5040100 (47.6)
Current/former smoker	1585590 (48.0)	151170 (62.0)	4883770 (48.7)	1616800 (47.9)	153375 (61.8)	5228590 (49.4)
Missing	116575 (3.5)	1880 (0.8)	455555 (4.5)	119535 (3.5)	1960 (0.8)	317925 (3.0)
Past medical history						
Baseline eGFR (ml/min/1.73m^2), median (IQR)	92.2 (78.8-104.4)	73.3 (55.1-88.5)	89.6 (75.3-102.6)	92.4 (79.1-104.7)	73.7 (55.3-88.9)	90.7 (76.5-103.5)
Previous acute kidney injury, n (%)	34100 (1.0)	35700 (14.6)	116575 (1.2)	34385 (1.0)	35890 (14.5)	75155 (0.7)
Cardiovascular diseases, n (%)	209455 (6.3)	90410 (37.1)	745695 (7.4)	211080 (6.3)	90685 (36.6)	600830 (5.7)
Diabetes mellitus, n (%)	305740 (9.3)	79230 (32.5)	976655 (9.7)	309365 (9.2)	79800 (32.2)	858160 (8.1)

Hypertension, n (%)	524300 (15.9)	131905 (54.1)	1748430 (17.4)	527430 (15.6)	132265 (53.3)	1833780 (17.3)
Immunosuppressive diseases, n (%)	56350 (1.7)	19295 (7.9)	179915 (1.8)	57030 (1.7)	19430 (7.8)	181545 (1.7)
Non-haematological cancer, n (%)	147115 (4.5)	50915 (20.9)	517520 (5.2)	148120 (4.4)	51135 (20.6)	519920 (4.9)
GP consultations previous year, median (IQR)	3 (1-8)	10 (4-20)	3 (0-8)	3 (1-8)	10 (4-20)	3 (0-7)
Hospital admissions previous 5 years, n (%)						
0	1954870 (59.2)	56005 (23.0)	6220305 (62.0)	1993135 (59.1)	56845 (22.9)	7784135 (73.5)
1	624525 (18.9)	39510 (16.2)	1736460 (17.3)	638555 (18.9)	40180 (16.2)	1538610 (14.5)
>1	721070 (21.8)	148330 (60.8)	2074770 (20.7)	741430 (22.0)	150995 (60.9)	1263865 (11.9)
COVID-19 vaccination status, n (%)						
Unvaccinated	1309745 (39.7)	116300 (47.7)	4470605 (44.6)	1319395 (39.1)	117375 (47.3)	N/A
1 vaccine dose	228360 (6.9)	9165 (3.8)	605545 (6.0)	231575 (6.9)	9440 (3.8)	N/A
2 vaccine doses	1176475 (35.6)	31175 (12.8)	2989875 (29.8)	1203045 (35.7)	32150 (13.0)	N/A
3 vaccine doses	576040 (17.5)	57555 (23.6)	1849475 (18.4)	609025 (18.1)	59200 (23.9)	N/A
4 vaccine doses	9845 (0.3)	29645 (12.2)	116040 (1.2)	10080 (0.3)	29855 (12.0)	N/A
COVID-19 wave, n (%)						
February 2020 – August 2020 (wild-type)	83325 (2.5)	26260 (10.8)	320440 (3.2)	83335 (2.5)	26265 (10.6)	N/A
September 2020 – June 2021 (Alpha variant)	989350 (30.0)	75145 (30.8)	3058780 (30.5)	989465 (29.3)	75150 (30.3)	N/A
July 2021 – November 2021 (Delta variant)	1024270 (31.0)	32785 (13.4)	3010410 (30.0)	1024390 (30.4)	32790 (13.2)	N/A
December 2021 – December 2022 (Omicron variants)	1203515 (36.5)	109655 (45.0)	3641900 (36.3)	1275925 (37.8)	113815 (45.9)	N/A

Counts rounded to nearest 5. eGFR = estimated glomerular filtration rate, GP = general practice, IQR = interquartile range, N/A = not applicable.

Table S3

Demographic, socioeconomic and clinical characteristics for COVID-19 cohort and an age-, sex- and sustainability and transformation partnership region-matched contemporary cohort compared to their respective cohorts for complete case matched analysis (i.e. after excluding individuals with any missing data for ethnicity, body mass index or smoking status).

	COVID-19 cohort		Matched cohort	
	Extracted	After excluding individuals with missing data	Extracted	After excluding individuals with missing data
Number of individuals	3544310	2516030	10031535	5649105
Follow-up (days), median (IQR)	446 (370-700)	441 (369-700)	410 (361-567)	408 (360-572)
Age (years), median (IQR)	44 (32-57)	48 (37-59)	44 (32-57)	49 (38-60)
Sex, n (%)				
Female	1886340 (53.2)	1464385 (58.2)	5306755 (52.9)	3381335 (59.9)
Male	1657970 (46.8)	1051640 (41.8)	4724780 (47.1)	2267770 (40.1)
Index of multiple deprivation, n (%)				
1 Most deprived	726765 (20.5)	506390 (20.1)	2104640 (21.0)	1139305 (20.2)
2	718950 (20.3)	507415 (20.2)	2004605 (20.0)	1114225 (19.7)
3	746720 (21.1)	533375 (21.2)	2104315 (21.0)	1197850 (21.2)
4	702980 (19.8)	504115 (20.0)	1981500 (19.8)	1139045 (20.2)
5 Least deprived	648890 (18.3)	464740 (18.5)	1836475 (18.3)	1058675 (18.7)
Ethnicity, n (%)				
White	2568170 (72.5)	2183415 (86.8)	7085150 (70.6)	4847455 (85.8)
South Asian	241010 (6.8)	198570 (7.9)	680615 (6.8)	439465 (7.8)
Black	74625 (2.1)	60525 (2.4)	233515 (2.3)	149565 (2.6)
Mixed	41485 (1.2)	32330 (1.3)	122310 (1.2)	72910 (1.3)
Other	50720 (1.4)	41185 (1.6)	237785 (2.4)	139715 (2.5)
Missing	568300 (16.0)	N/A	1672165 (16.7)	N/A
Region, n (%)				
East Midlands	648825 (18.3)	462455 (18.4)	1844310 (18.4)	1047515 (18.5)

East	792995 (22.4)	555860 (22.1)	2240430 (22.3)	1234630 (21.9)
London	217915 (6.1)	167190 (6.6)	618245 (6.2)	387595 (6.9)
North East	193870 (5.5)	139590 (5.5)	552690 (5.5)	318895 (5.6)
North West	352505 (9.9)	249145 (9.9)	995750 (9.9)	565850 (10.0)
South East	209785 (5.9)	144995 (5.8)	595370 (5.9)	314850 (5.6)
South West	430780 (12.2)	299500 (11.9)	1224560 (12.2)	666195 (11.8)
West Midlands	156475 (4.4)	111505 (4.4)	448735 (4.5)	252930 (4.5)
Yorkshire and The Humber	541155 (15.3)	385795 (15.3)	1511450 (15.1)	860655 (15.2)
Urban/rural, n (%)				
Urban	2892590 (81.6)	2045610 (81.3)	8043555 (80.2)	4484235 (79.4)
Rural	651715 (18.4)	470420 (18.7)	1987985 (19.8)	1164870 (20.6)
Body mass index, n (%)				
<18.5 kg/m^2	58510 (1.7)	44510 (1.8)	205770 (2.1)	121860 (2.2)
18.5-24.9 kg/m^2	1039705 (29.3)	854660 (34.0)	3155620 (31.5)	2081235 (36.8)
25.0-29.9 kg/m^2	1023430 (28.9)	868405 (34.5)	2750620 (27.4)	1907100 (33.8)
30.0-34.9 kg/m^2	541325 (15.3)	459140 (18.2)	1365695 (13.6)	954435 (16.9)
35.0-39.9 kg/m^2	224720 (6.3)	188695 (7.5)	548850 (5.5)	380785 (6.7)
≥40.0 kg/m^2	120670 (3.4)	100615 (4.0)	294820 (2.9)	203695 (3.6)
Missing	535945 (15.1)	N/A	1710165 (17.0)	N/A
Smoking, n (%)				
Non-smoker	1689090 (47.7)	1186445 (47.2)	4692210 (46.8)	2638100 (46.7)
Current/former smoker	1736760 (49.0)	1329585 (52.8)	4883770 (48.7)	3011005 (53.3)
Missing	118455 (3.3)	N/A	455555 (4.5)	N/A
Past medical history				
Baseline eGFR (ml/min/1.73m^2), median (IQR)	90.3 (75.9-103.1)	89.4 (75.4-101.8)	89.6 (75.3-102.6)	88.5 (74.6-100.8)
Previous acute kidney injury, n (%)	69805 (2.0)	54340 (2.2)	116575 (1.2)	75165 (1.3)
Cardiovascular diseases, n (%)	299865 (8.5)	243305 (9.7)	745695 (7.4)	506305 (9.0)
Diabetes mellitus, n (%)	384975 (10.9)	321315 (12.8)	976655 (9.7)	688585 (12.2)

Hypertension, n (%)	656205 (18.5)	544935 (21.7)	1748430 (17.4)	1222810 (21.6)
Immunosuppressive diseases, n (%)	75645 (2.1)	62090 (2.5)	179915 (1.8)	123550 (2.2)
Non-haematological cancer, n (%)	198035 (5.6)	162555 (6.5)	517520 (5.2)	357485 (6.3)
GP consultations previous year, median (IQR)	4.0 (1.0-9.0)	4.0 (1.0-10.0)	3.0 (0.0-8.0)	4.0 (1.0-9.0)
Hospital admissions previous 5 years, n (%)				
0	2010875 (56.7)	1342040 (53.3)	6220305 (62.0)	3246110 (57.5)
1	664035 (18.7)	492395 (19.6)	1736460 (17.3)	1061680 (18.8)
>1	869400 (24.5)	681590 (27.1)	2074770 (20.7)	1341315 (23.7)
COVID-19 vaccination status, n (%)				
Unvaccinated	1426045 (40.2)	954230 (37.9)	4470605 (44.6)	2289055 (40.5)
1 vaccine dose	237525 (6.7)	140725 (5.6)	605545 (6.0)	278620 (4.9)
2 vaccine doses	1207650 (34.1)	881245 (35.0)	2989875 (29.8)	1771835 (31.4)
3 vaccine doses	633595 (17.9)	507460 (20.2)	1849475 (18.4)	1230265 (21.8)
4 vaccine doses	39495 (1.1)	32365 (1.3)	116040 (1.2)	79335 (1.4)
COVID-19 wave, n (%)				
February 2020 – August 2020 (wild-type)	109590 (3.1)	82335 (3.3)	320440 (3.2)	192335 (3.4)
September 2020 – June 2021 (Alpha variant)	1064495 (30.0)	743585 (29.6)	3058780 (30.5)	1682130 (29.8)
July 2021 – November 2021 (Delta variant)	1057055 (29.8)	745460 (29.6)	3010410 (30.0)	1683260 (29.8)
December 2021 – December 2022 (Omicron variants)	1313170 (37.1)	944645 (37.5)	3641900 (36.3)	2091375 (37.0)

Counts rounded to nearest 5. eGFR = estimated glomerular filtration rate, GP = general practice, IQR = interquartile range, N/A = not applicable.

Table S4

Fully-adjusted hazard ratio and adjusted rate difference estimates for kidney failure (i.e. incident dialysis, kidney transplantation or estimated glomerular filtration rate (eGFR) <15 ml/min/1.73m^2), 50% reduction in eGFR (composite outcome including incident kidney failure), and death after COVID-19 compared to an age-, sex- and sustainability and transformation partnership region-matched cohort, overall and by follow-up period (in days since index date, i.e., 28 days after first COVID-19 infection record), stratified by hospitalisation status (**Figure 3**).

	Follow-up period	Events in COVID-19 group	Events in matched group	Fully-adjusted HR (95% CI)	Adjusted rate difference (/100,000 person-years) (95% CI)
Kidney failure					
COVID-19 overall	Overall	4545	4980	1.93 (1.84-2.03)	N/A
	0-29 days	1620	330	13.65 (11.59-16.07)	755.56 (744.95-764.56)
	30-89 days	500	645	1.53 (1.31-1.79)	43.28 (29.57-55.14)
	90-179 days	565	895	1.10 (0.95-1.27)	8.63 (-5.00-20.18)
	180+ days	1830	3080	1.04 (0.97-1.12)	3.14 (-2.53-8.75)
COVID-19 non-hospitalised	Overall	1690	4980	0.85 (0.79-0.90)	N/A
	0-29 days	100	330	0.80 (0.60-1.07)	-13.56 (-36.17-3.55)
	30-89 days	205	645	0.91 (0.74-1.10)	-5.43 (-19.28-4.99)
	90-179 days	275	895	0.84 (0.71-0.99)	-9.36 (-20.07--0.50)
	180+ days	1100	3080	0.84 (0.77-0.91)	-9.96 (-15.61--5.17)
COVID-19 hospitalised	Overall	2855	4980	7.74 (7.00-8.56)	N/A
	0-29 days	1520	330	75.93 (52.68-109.43)	10438.65 (10377.16-10481.30)
	30-89 days	300	645	4.17 (3.10-5.61)	856.71 (763.43-926.08)
	90-179 days	290	895	2.11 (1.57-2.85)	428.73 (295.88-529.02)
	180+ days	730	3080	2.20 (1.87-2.58)	292.15 (249.18-328.00)
50% reduction in eGFR					
COVID-19 overall	Overall	7070	8710	1.62 (1.56-1.68)	N/A
	0-29 days	1295	380	7.84 (6.64-9.27)	1239.29 (1206.54-1267.24)
	30-89 days	925	965	1.96 (1.76-2.19)	248.44 (219.04-275.62)
	90-179 days	1070	1490	1.46 (1.33-1.62)	125.40 (98.75-152.32)
	180+ days	3735	5820	1.25 (1.18-1.31)	73.46 (56.03-86.92)

COVID-19 non-hospitalised	Overall	3105	8710	0.99 (0.95-1.04)	N/A
	0-29 days	130	380	1.10 (0.83-1.45)	14.76 (-33.25-50.38)
	30-89 days	325	965	1.05 (0.90-1.22)	9.55 (-22.28-36.15)
	90-179 days	495	1490	1.04 (0.92-1.18)	7.86 (-17.76-31.16)
	180+ days	2140	5820	0.97 (0.91-1.03)	-7.20 (-23.03-6.78)
COVID-19 hospitalised	Overall	3965	8710	3.49 (3.25-3.75)	N/A
	0-29 days	1170	380	21.62 (16.49-28.34)	10061.08 (9909.29-10176.78)
	30-89 days	600	965	3.92 (3.29-4.66)	2206.90 (2062.18-2326.92)
	90-179 days	575	1490	2.54 (2.13-3.02)	1314.59 (1150.28-1450.27)
	180+ days	1600	5820	2.19 (1.99-2.41)	889.50 (814.37-957.73)
Death					
COVID-19 overall	Overall	57530	54085	2.22 (2.19-2.25)	N/A
	0-29 days	11175	3130	7.45 (7.15-7.76)	4866.77 (4835.11-4896.91)
	30-89 days	9815	6105	3.32 (3.22-3.43)	1712.93 (1690.00-1736.61)
	90-179 days	8605	8865	2.02 (1.96-2.08)	729.47 (707.58-750.10)
	180+ days	27510	35725	1.58 (1.55-1.60)	450.12 (435.10-459.83)
COVID-19 non-hospitalised	Overall	23375	54085	1.27 (1.25-1.29)	N/A
	0-29 days	1480	3130	1.54 (1.44-1.64)	281.53 (245.32-313.32)
	30-89 days	2495	6105	1.32 (1.26-1.38)	161.90 (137.81-183.90)
	90-179 days	3380	8865	1.20 (1.15-1.25)	100.64 (78.76-120.77)
	180+ days	15905	35725	1.27 (1.25-1.30)	160.60 (151.09-174.33)
COVID-19 hospitalised	Overall	34155	54085	4.93 (4.83-5.04)	N/A
	0-29 days	9695	3130	19.36 (18.09-20.72)	63583.45 (63340.32-63810.76)
	30-89 days	7320	6105	7.84 (7.44-8.26)	23817.24 (23630.03-23994.29)
	90-179 days	5225	8865	4.08 (3.88-4.29)	10990.30 (10806.37-11164.97)
	180+ days	11605	35725	2.54 (2.47-2.62)	5095.32 (5001.55-5196.35)

Event counts rounded to nearest 5. Models adjusted for ethnicity, deprivation, rural or urban, body mass index, smoking status, baseline eGFR (with “no baseline eGFR measurement” treated as a categorical variable), previous acute kidney injury, cardiovascular diseases, diabetes

mellitus, hypertension, immunosuppressive diseases, non-haematological cancer, general practice consultations in the previous year, hospital admissions in the previous five years, COVID-19 vaccination status and COVID-19 wave. N/A = not applicable.

Table S5

Proportional hazards assumption for each outcome (kidney failure, 50% reduction in eGFR, and death) tested using Schoenfeld residuals: globally, and COVID-19 as the exposure. For kidney failure, p-values were obtained for COVID-19 overall, and by severity (COVID-19 non-hospitalised and COVID-19 hospitalised).

	P-value for non-proportionality of hazards				
	Kidney failure			50% reduction in eGFR	Death
	COVID-19 overall	COVID-19 non-hospitalised	COVID-19 hospitalised	COVID-19 overall	COVID-19 overall
Global test	<0.0001	0.0005	<0.0001	<0.0001	<0.0001
COVID-19	<0.0001	0.6669	<0.0001	<0.0001	<0.0001

Table S6

Demographic, socioeconomic and clinical characteristics for i) the COVID-19 and an age-, sex- and sustainability and transformation partnership (STP) region-matched cohorts, and ii) the COVID-19 and an age-, sex- and STP region-matched historical cohorts.

	Contemporary (main analysis)		Historical (sensitivity analysis)	
	COVID-19 cohort	Matched cohort	COVID-19 cohort	Matched cohort
Number of individuals	3544310	10031535	3621140	10586610
Follow-up (days), median (IQR)	446 (370-700)	410 (361-567)	441 (369-695)	444 (369-701)
Age (years), median (IQR)	44 (32-57)	44 (32-57)	44 (32-56)	44 (32-56)
Sex, n (%)				
Female	1886340 (53.2)	5306755 (52.9)	1960865 (54.2)	5723975 (54.1)
Male	1657970 (46.8)	4724780 (47.1)	1660275 (45.8)	4862635 (45.9)
Index of multiple deprivation, n (%)				
1 Most deprived	726765 (20.5)	2104640 (21.0)	749415 (20.7)	2204035 (20.8)
2	718950 (20.3)	2004605 (20.0)	734815 (20.3)	2112930 (20.0)
3	746720 (21.1)	2104315 (21.0)	760135 (21.0)	2220980 (21.0)
4	702980 (19.8)	1981500 (19.8)	715910 (19.8)	2095920 (19.8)
5 Least deprived	648890 (18.3)	1836475 (18.3)	660870 (18.3)	1952740 (18.4)
Ethnicity, n (%)				
White	2568170 (72.5)	7085150 (70.6)	2622215 (72.4)	7591135 (71.7)
South Asian	241010 (6.8)	680615 (6.8)	245935 (6.8)	681460 (6.4)
Black	74625 (2.1)	233515 (2.3)	75900 (2.1)	225075 (2.1)
Mixed	41485 (1.2)	122310 (1.2)	42500 (1.2)	115555 (1.1)
Other	50720 (1.4)	237785 (2.4)	51745 (1.4)	202990 (1.9)
Missing	568300 (16.0)	1672165 (16.7)	582850 (16.1)	1770395 (16.7)
Region, n (%)				
East Midlands	648825 (18.3)	1844310 (18.4)	664945 (18.4)	1960365 (18.5)
East	792995 (22.4)	2240430 (22.3)	804150 (22.2)	2357650 (22.3)
London	217915 (6.1)	618245 (6.2)	218120 (6.0)	633970 (6.0)

North East	193870 (5.5)	552690 (5.5)	201310 (5.6)	592770 (5.6)
North West	352505 (9.9)	995750 (9.9)	369160 (10.2)	1079210 (10.2)
South East	209785 (5.9)	595370 (5.9)	210645 (5.8)	614510 (5.8)
South West	430780 (12.2)	1224560 (12.2)	432210 (11.9)	1267650 (12.0)
West Midlands	156475 (4.4)	448735 (4.5)	160580 (4.4)	473890 (4.5)
Yorkshire and The Humber	541155 (15.3)	1511450 (15.1)	560015 (15.5)	1606600 (15.2)
Urban/rural, n (%)				
Urban	2892590 (81.6)	8043555 (80.2)	2958020 (81.7)	8497210 (80.3)
Rural	651715 (18.4)	1987985 (19.8)	663120 (18.3)	2089400 (19.7)
Body mass index, n (%)				
<18.5 kg/m^2	58510 (1.7)	205770 (2.1)	60705 (1.7)	209820 (2.0)
18.5-24.9 kg/m^2	1039705 (29.3)	3155620 (31.5)	1065965 (29.4)	3386070 (32.0)
25.0-29.9 kg/m^2	1023430 (28.9)	2750620 (27.4)	1041345 (28.8)	2955225 (27.9)
30.0-34.9 kg/m^2	541325 (15.3)	1365695 (13.6)	551655 (15.2)	1443525 (13.6)
35.0-39.9 kg/m^2	224720 (6.3)	548850 (5.5)	229995 (6.4)	567495 (5.4)
≥40.0 kg/m^2	120670 (3.4)	294820 (2.9)	124260 (3.4)	292780 (2.8)
Missing	535945 (15.1)	1710165 (17.0)	547210 (15.1)	1731705 (16.4)
Smoking, n (%)				
Non-smoker	1689090 (47.7)	4692210 (46.8)	1729475 (47.8)	5040100 (47.6)
Current/former smoker	1736760 (49.0)	4883770 (48.7)	1770175 (48.9)	5228590 (49.4)
Missing	118455 (3.3)	455555 (4.5)	121490 (3.4)	317925 (3.0)
Past medical history				
Baseline eGFR (ml/min/1.73m^2), median (IQR)	90.3 (75.9-103.1)	89.6 (75.3-102.6)	90.5 (76.2-103.4)	90.7 (76.5-103.5)
Previous acute kidney injury, n (%)	69805 (2.0)	116575 (1.2)	70280 (1.9)	75155 (0.7)
Cardiovascular diseases, n (%)	299865 (8.5)	745695 (7.4)	301765 (8.3)	600830 (5.7)
Diabetes mellitus, n (%)	384975 (10.9)	976655 (9.7)	389165 (10.7)	858160 (8.1)
Hypertension, n (%)	656205 (18.5)	1748430 (17.4)	659695 (18.2)	1833780 (17.3)
Immunosuppressive diseases, n (%)	75645 (2.1)	179915 (1.8)	76460 (2.1)	181545 (1.7)

Non-haematological cancer, n (%)	198035 (5.6)	517520 (5.2)	199255 (5.5)	519920 (4.9)
GP consultations previous year, median (IQR)	4 (1-9)	3 (0-8)	4 (1-9)	3 (0-7)
Hospital admissions previous 5 years, n (%)				
0	2010875 (56.7)	6220305 (62.0)	2049975 (56.6)	7784135 (73.5)
1	664035 (18.7)	1736460 (17.3)	678735 (18.7)	1538610 (14.5)
>1	869400 (24.5)	2074770 (20.7)	892430 (24.6)	1263865 (11.9)
COVID-19 vaccination status, n (%)				
Unvaccinated	1426045 (40.2)	4470605 (44.6)	1436770 (39.7)	N/A
1 vaccine dose	237525 (6.7)	605545 (6.0)	241015 (6.7)	N/A
2 vaccine doses	1207650 (34.1)	2989875 (29.8)	1235200 (34.1)	N/A
3 vaccine doses	633595 (17.9)	1849475 (18.4)	668220 (18.5)	N/A
4 vaccine doses	39495 (1.1)	116040 (1.2)	39930 (1.1)	N/A
COVID-19 wave, n (%)				
February 2020 – August 2020 (wild-type)	109590 (3.1)	320440 (3.2)	109600 (3.0)	N/A
September 2020 – June 2021 (Alpha variant)	1064495 (30.0)	3058780 (30.5)	1064615 (29.4)	N/A
July 2021 – November 2021 (Delta variant)	1057055 (29.8)	3010410 (30.0)	1057185 (29.2)	N/A
December 2021 – December 2022 (Omicron variants)	1313170 (37.1)	3641900 (36.3)	1389740 (38.4)	N/A

Counts rounded to nearest 5. eGFR = estimated glomerular filtration rate, GP = general practice, IQR = interquartile range, N/A = not applicable.

Table S7

Fully-adjusted hazard ratio and adjusted rate difference estimates for kidney failure (i.e. incident dialysis, kidney transplantation or estimated glomerular filtration rate (eGFR) <15 ml/min/1.73m^2), 50% reduction in eGFR (composite outcome including incident kidney failure), and death after COVID-19 compared to an age-, sex- and sustainability and transformation partnership region-matched **historical cohort**, overall and by follow-up period (in days since index date, i.e., 28 days after first COVID-19 infection record), stratified by hospitalisation status (**Figure S2**).

	Follow-up period	Events in COVID-19 group	Events in matched group	Fully-adjusted HR (95% CI)	Adjusted rate difference (/100,000 person-years) (95% CI)
Kidney failure					
COVID-19 overall	Overall	4550	4995	1.75 (1.66-1.84)	N/A
	0-29 days	1630	420	9.01 (7.89-10.29)	708.32 (695.77-719.32)
	30-89 days	500	690	1.21 (1.03-1.43)	21.06 (3.53-36.49)
	90-179 days	565	925	1.04 (0.89-1.22)	3.55 (-11.39-16.62)
	180+ days	1830	2915	0.98 (0.91-1.06)	-1.64 (-7.93-4.54)
COVID-19 non-hospitalised	Overall	1685	4995	0.81 (0.75-0.86)	N/A
	0-29 days	100	420	0.57 (0.45-0.72)	-39.70 (-64.33--20.47)
	30-89 days	200	690	0.75 (0.61-0.92)	-17.31 (-33.21--4.52)
	90-179 days	275	925	0.74 (0.62-0.89)	-16.75 (-29.22--5.89)
	180+ days	1095	2915	0.86 (0.79-0.93)	-8.31 (-13.57--3.84)
COVID-19 hospitalised	Overall	2865	4995	6.35 (5.74-7.04)	N/A
	0-29 days	1530	420	47.28 (35.19-63.52)	10267.46 (10191.24-10324.19)
	30-89 days	300	690	3.35 (2.47-4.56)	778.06 (660.10-865.92)
	90-179 days	290	925	2.53 (1.82-3.53)	485.08 (361.39-574.89)
	180+ days	735	2915	1.58 (1.36-1.85)	197.08 (142.11-246.67)
50% reduction in eGFR					
COVID-19 overall	Overall	7050	7790	1.64 (1.57-1.71)	N/A
	0-29 days	1290	465	4.86 (4.21-5.61)	1100.57 (1056.55-1138.69)
	30-89 days	920	1050	1.65 (1.48-1.86)	194.58 (160.20-228.38)
	90-179 days	1065	1580	1.22 (1.10-1.36)	69.93 (35.25-102.65)
	180+ days	3730	4640	1.45 (1.37-1.54)	112.48 (97.88-127.08)

COVID-19 non-hospitalised	Overall	3095	7790	1.06 (1.00-1.12)	N/A
	0-29 days	130	465	0.77 (0.61-0.97)	-47.40 (-101.46--4.91)
	30-89 days	320	1050	0.88 (0.76-1.03)	-26.31 (-60.93-5.62)
	90-179 days	490	1580	0.76 (0.66-0.87)	-62.42 (-101.82--29.54)
	180+ days	2135	4640	1.22 (1.14-1.31)	41.34 (28.15-54.25)
COVID-19 hospitalised	Overall	3955	7790	3.40 (3.16-3.67)	N/A
	0-29 days	1160	465	16.40 (12.63-21.31)	9746.68 (9557.76-9892.50)
	30-89 days	605	1050	3.56 (2.92-4.34)	2130.58 (1948.17-2280.16)
	90-179 days	575	1580	3.00 (2.48-3.62)	1433.77 (1283.46-1556.56)
	180+ days	1595	4640	2.15 (1.94-2.38)	872.07 (789.98-945.35)
Death					
COVID-19 overall	Overall	57160	58800	1.76 (1.74-1.79)	N/A
	0-29 days	11100	3500	5.56 (5.34-5.79)	4447.79 (4407.60-4486.53)
	30-89 days	9785	7020	2.39 (2.31-2.47)	1380.28 (1345.89-1412.44)
	90-179 days	8555	9260	1.58 (1.53-1.63)	512.00 (483.15-539.08)
	180+ days	27300	38665	1.27 (1.25-1.30)	254.08 (239.03-275.80)
COVID-19 non-hospitalised	Overall	23195	58800	1.03 (1.02-1.05)	N/A
	0-29 days	1475	3500	1.11 (1.05-1.18)	76.93 (36.97-118.42)
	30-89 days	2480	7020	0.93 (0.89-0.98)	-48.48 (-79.60--13.14)
	90-179 days	3350	9260	0.95 (0.91-0.99)	-30.56 (-57.43--5.87)
	180+ days	15785	38665	1.06 (1.03-1.08)	41.64 (21.43-54.50)
COVID-19 hospitalised	Overall	33965	58800	3.81 (3.72-3.89)	N/A
	0-29 days	9625	3500	15.38 (14.39-16.43)	61313.78 (61020.44-61586.27)
	30-89 days	7305	7020	5.68 (5.38-5.99)	22094.16 (21830.91-22338.48)
	90-179 days	5205	9260	3.19 (3.03-3.35)	9800.47 (9564.16-10014.21)
	180+ days	11520	38665	1.95 (1.90-2.01)	4046.19 (3934.10-4173.33)

Event counts rounded to nearest 5. Models adjusted for ethnicity, deprivation, rural or urban, body mass index, smoking status, baseline eGFR (with “no baseline eGFR measurement” treated as a categorical variable), previous acute kidney injury, cardiovascular diseases, diabetes

mellitus, hypertension, immunosuppressive diseases, non-haematological cancer, general practice consultations in the previous year and hospital admissions in the previous five years. N/A = not applicable.

Table S8

Fully-adjusted hazard ratio estimates for kidney failure (i.e. incident dialysis, kidney transplantation or estimated glomerular filtration rate (eGFR) <15 ml/min/1.73m^2), stratified by potential effect modifiers, for COVID-19 compared to an age-, sex- and sustainability and transformation partnership region-matched cohort (**Figure 4**).

	Events in COVID-19 group	Events in matched group	Fully-adjusted HR (95% CI)	P-value for interaction
Age				
18-39	280	365	1.51 (1.23-1.84)	0.0002
40-49	400	535	1.72 (1.44-2.04)	
50-59	745	1000	1.86 (1.64-2.12)	
60-69	960	1000	2.27 (2.00-2.57)	
70-79	1045	1055	2.27 (1.99-2.59)	
80+	1115	1615	1.69 (1.48-1.93)	
Sex				
Female	1825	2480	1.63 (1.49-1.79)	<0.0001
Male	2720	3085	2.17 (2.01-2.35)	
Ethnicity				
White	1145	4815	1.82 (1.71-1.94)	<0.0001
South Asian	1025	430	2.43 (1.90-3.11)	
Black	960	190	4.50 (2.92-6.92)	
Mixed	795	60	2.02 (0.97-4.18)	
Other	620	70	4.11 (2.11-8.01)	
Diabetes mellitus				
No diabetes mellitus	2270	3105	1.78 (1.65-1.92)	0.0004
Diabetes mellitus	2275	2460	2.34 (2.07-2.65)	
Baseline eGFR				
≥105	140	180	1.51 (0.98-2.31)	0.0066
90-104	345	360	2.29 (1.81-2.89)	
75-89	455	435	2.68 (2.21-3.25)	
60-74	430	435	2.17 (1.77-2.68)	

45-59	480	530	1.71 (1.38-2.14)	
30-44	675	710	2.08 (1.61-2.67)	
15-29	1270	1870	1.32 (0.91-1.91)	
COVID-19 vaccination status				
Unvaccinated	2450	2690	2.39 (2.20-2.60)	<0.0001
1 vaccine dose	200	295	1.17 (0.86-1.60)	
2 vaccine doses	790	1360	1.08 (0.94-1.24)	
3 vaccine doses	900	1110	1.89 (1.64-2.19)	
4 vaccine doses	205	105	9.43 (6.05-14.69)	
COVID-19 vaccination status (cases up to March 2022 only)				
Unvaccinated	2420	2680	2.35 (2.16-2.56)	<0.0001
1 vaccine dose	190	295	1.12 (0.82-1.53)	
2 vaccine doses	750	1350	1.07 (0.93-1.23)	
3 vaccine doses	475	960	1.21 (1.01-1.44)	
4 vaccine doses	N/A	N/A	N/A	
COVID-19 wave				
February 2020 – August 2020 (wild-type)	745	550	3.77 (3.20-4.44)	<0.0001
September 2020 – June 2021 (Alpha variant)	1685	2240	1.82 (1.65-2.00)	
July 2021 – November 2021 (Delta variant)	735	1325	1.28 (1.12-1.45)	
December 2021 – December 2022 (Omicron variants)	1380	1455	2.11 (1.89-2.36)	
COVID-19 wave (cases up to March 2022 only)				
February 2020 – August 2020 (wild-type)	745	550	3.77 (3.20-4.45)	<0.0001
September 2020 – June 2021 (Alpha variant)	1685	2240	1.81 (1.65-1.99)	
July 2021 – November 2021 (Delta variant)	735	1325	1.27 (1.11-1.45)	
December 2021 – March 2022 (Omicron BA.1 variant)	670	1180	1.22 (1.06-1.41)	

Event counts rounded to nearest 5. Models adjusted for ethnicity, deprivation, rural or urban, body mass index, smoking status, baseline eGFR (with “no baseline eGFR measurement” treated as a categorical variable), previous acute kidney injury, cardiovascular diseases, diabetes mellitus, hypertension, immunosuppressive diseases, non-haematological cancer, general practice consultations in the previous year, hospital admissions in the previous five years, COVID-19 vaccination status and COVID-19 wave. *P*-values for interaction obtained by likelihood ratio tests comparing models with and without interaction term.

Table S9

Fully-adjusted hazard ratio estimates for kidney failure (i.e. incident dialysis, kidney transplantation or estimated glomerular filtration rate (eGFR) <15 ml/min/1.73m^2), stratified by potential effect modifiers, for COVID-19 compared to an age-, sex- and sustainability and transformation partnership region-matched **historical cohort** (**Figure S4**).

	Events in COVID-19 group	Events in matched group	Fully-adjusted HR (95% CI)	*P*-value for interaction
Age				
18-39	285	350	1.32 (1.08-1.60)	0.0001
40-49	420	545	1.42 (1.20-1.68)	
50-59	755	850	1.80 (1.58-2.05)	
60-69	960	890	2.09 (1.84-2.37)	
70-79	1040	1020	1.92 (1.68-2.19)	
80+	1090	1345	1.64 (1.42-1.89)	
Sex				
Female	1825	2220	1.48 (1.35-1.63)	<0.0001
Male	2725	2775	1.96 (1.81-2.12)	
Ethnicity				
White	3700	4305	1.69 (1.58-1.81)	0.0063
South Asian	505	415	1.81 (1.43-2.30)	
Black	215	160	3.64 (2.43-5.47)	
Mixed	50	55	1.35 (0.67-2.70)	
Other	75	60	1.86 (1.03-3.35)	
Diabetes mellitus				
No diabetes mellitus	2270	2855	1.73 (1.60-1.87)	0.7138
Diabetes mellitus	2280	2145	1.78 (1.57-2.02)	
Baseline eGFR				
≥105	145	180	1.03 (0.69-1.53)	0.0002
90-104	350	355	1.43 (1.14-1.80)	
75-89	455	455	1.97 (1.63-2.38)	
60-74	430	390	2.15 (1.74-2.65)	

45-59	475	500	2.10 (1.67-2.65)	
30-44	675	670	1.74 (1.33-2.27)	
15-29	1260	1630	0.98 (0.67-1.43)	

Event counts rounded to nearest 5. Models adjusted for ethnicity, deprivation, rural or urban, body mass index, smoking status, baseline eGFR (with “no baseline eGFR measurement” treated as a categorical variable), previous acute kidney injury, cardiovascular diseases, diabetes mellitus, hypertension, immunosuppressive diseases, non-haematological cancer, general practice consultations in the previous year and hospital admissions in the previous five years. *P*-values for interaction obtained by likelihood ratio tests comparing models with and without interaction term.

Table S10

Fully-adjusted hazard ratio estimates for kidney failure (i.e. incident dialysis, kidney transplantation or estimated glomerular filtration rate (eGFR) <15 ml/min/1.73m^2), stratified by potential effect modifiers, for COVID-19 stratified by hospitalisation status compared to an age-, sex- and sustainability and transformation partnership region-matched cohort (**Figure S5**).

	Events in COVID-19 group		Events in matched group	Fully-adjusted hazard ratio (95% CI)	
	Non-hospitalised	Hospitalised		Non-hospitalised COVID-19	Hospitalised COVID-19
Age					
18-39	145	130	365	0.86 (0.67-1.10)	17.41 (8.36-36.26)
40-49	185	215	535	0.81 (0.65-1.02)	19.72 (11.21-34.68)
50-59	275	470	1000	0.72 (0.60-0.86)	17.94 (12.67-25.42)
60-69	330	630	1000	0.81 (0.68-0.96)	16.82 (12.63-22.39)
70-79	345	700	1055	0.92 (0.76-1.12)	7.35 (5.89-9.18)
80+	405	705	1615	1.02 (0.84-1.24)	2.82 (2.33-3.40)
Sex					
Female	760	1065	2480	0.84 (0.75-0.95)	6.36 (5.33-7.60)
Male	930	1790	3085	0.85 (0.76-0.95)	8.66 (7.53-9.97)
Ethnicity					
White	1385	2315	4815	0.82 (0.75-0.89)	7.20 (6.38-8.12)
South Asian	210	295	430	0.97 (0.71-1.33)	10.12 (6.72-15.26)
Black	60	155	190	1.39 (0.79-2.46)	16.34 (8.54-31.27)
Mixed	10	40	60	0.70 (0.25-1.96)	9.39 (2.97-29.70)
Other	20	50	70	1.53 (0.63-3.69)	20.65 (6.20-68.72)
Diabetes mellitus					
No diabetes mellitus	925	1345	3105	0.83 (0.75-0.91)	8.97 (7.75-10.38)
Diabetes mellitus	765	1510	2460	0.90 (0.76-1.06)	6.11 (5.10-7.33)
Baseline eGFR					
≥105	50	85	180	0.71 (0.41-1.22)	6.89 (3.15-15.06)
90-104	125	220	360	0.92 (0.67-1.24)	10.73 (7.08-16.28)
75-89	145	310	435	1.02 (0.79-1.33)	10.29 (7.60-13.91)

60-74	155	275	435	0.96 (0.73-1.26)	6.97 (5.08-9.55)
45-59	155	325	530	0.80 (0.59-1.08)	3.70 (2.71-5.05)
30-44	245	430	710	1.27 (0.91-1.78)	3.30 (2.34-4.65)
15-29	540	730	1870	0.91 (0.55-1.52)	1.73 (1.06-2.82)
COVID-19 vaccination status					
Unvaccinated	840	1610	2690	0.94 (0.84-1.06)	8.47 (7.34-9.77)
1 vaccine dose	90	110	295	0.63 (0.42-0.92)	5.10 (2.81-9.26)
2 vaccine doses	430	360	1360	0.74 (0.63-0.87)	5.46 (3.95-7.55)
3 vaccine doses	315	585	1110	0.84 (0.69-1.02)	7.28 (5.58-9.49)
4 vaccine doses	15	190	105	1.10 (0.34-3.59)	9.08 (5.67-14.52)
COVID-19 vaccination status (cases up to March 2022 only)					
Unvaccinated	840	1580	2680	0.94 (0.83-1.05)	8.30 (7.18-9.59)
1 vaccine dose	90	100	295	0.62 (0.42-0.91)	4.70 (2.56-8.63)
2 vaccine doses	430	320	1350	0.73 (0.62-0.85)	5.46 (3.87-7.69)
3 vaccine doses	285	190	960	0.81 (0.66-0.99)	5.97 (3.85-9.24)
4 vaccine doses	N/A	N/A	N/A	N/A	N/A
COVID-19 wave					
February 2020 – August 2020 (wild-type)	205	535	550	1.23 (0.94-1.60)	8.74 (6.89-11.08)
September 2020 – June 2021 (Alpha variant)	685	1000	2240	0.87 (0.76-0.98)	6.83 (5.73-8.15)
July 2021 – November 2021 (Delta variant)	390	345	1325	0.75 (0.64-0.88)	7.74 (5.61-10.67)
December 2021 – December 2022 (Omicron variants)	405	975	1455	0.81 (0.68-0.95)	8.36 (6.81-10.27)
COVID-19 wave (cases up to March 2022 only)					
February 2020 – August 2020 (wild-type)	205	535	550	1.21 (0.93-1.58)	8.74 (6.88-11.10)
September 2020 – June 2021 (Alpha variant)	685	1000	2240	0.86 (0.76-0.98)	6.80 (5.70-8.11)
July 2021 – November 2021 (Delta variant)	390	345	1325	0.75 (0.64-0.88)	7.77 (5.62-10.75)
December 2021 – March 2022 (Omicron BA.1 variant)	360	305	1180	0.77 (0.65-0.92)	7.41 (5.13-10.70)

Event counts rounded to nearest 5. Models adjusted for ethnicity, deprivation, rural or urban, body mass index, smoking status, baseline eGFR (with “no baseline eGFR measurement” treated as a categorical variable), previous acute kidney injury, cardiovascular diseases, diabetes mellitus, hypertension, immunosuppressive diseases, non-haematological cancer, general practice consultations in the previous year, hospital

admissions in the previous five years, COVID-19 vaccination status and COVID-19 wave. Age in years. Baseline eGFR in ml/min/1.73m^2. CI = confidence interval.

Table S11

Fully-adjusted hazard ratio estimates for kidney failure (i.e. incident dialysis, kidney transplantation or estimated glomerular filtration rate (eGFR) <15 ml/min/1.73m^2), stratified by potential effect modifiers, for COVID-19 stratified by hospitalisation status compared to an age-, sex- and sustainability and transformation partnership region-matched historical cohort (**Figure S6**).

	Events in COVID-19 group		Events in matched group	Fully-adjusted hazard ratio (95% CI)	
	Non-hospitalised	Non-hospitalised		Non-hospitalised COVID-19	Hospitalised COVID-19
Age					
18-39	150	135	350	0.79 (0.62-1.00)	13.34 (6.71-26.52)
40-49	190	230	545	0.70 (0.56-0.87)	14.93 (9.10-24.51)
50-59	275	480	850	0.68 (0.57-0.82)	18.58 (12.93-26.72)
60-69	330	630	890	0.77 (0.64-0.91)	14.08 (10.63-18.64)
70-79	340	700	1020	0.87 (0.71-1.05)	4.92 (3.98-6.08)
80+	400	690	1345	1.18 (0.95-1.45)	2.28 (1.88-2.77)
Sex					
Female	755	1070	2220	0.80 (0.71-0.91)	5.22 (4.37-6.23)
Male	930	1795	2775	0.80 (0.72-0.90)	7.09 (6.18-8.14)
Ethnicity					
White	1380	2320	4305	0.81 (0.74-0.88)	5.96 (5.29-6.72)
South Asian	210	295	415	0.83 (0.62-1.13)	7.30 (4.92-10.84)
Black	60	155	160	1.01 (0.59-1.72)	16.84 (8.67-32.71)
Mixed	10	40	55	0.37 (0.14-0.99)	5.54 (1.94-15.82)
Other	20	50	60	0.55 (0.25-1.25)	10.07 (3.84-26.40)
Diabetes mellitus					
No diabetes mellitus	925	1345	2855	0.83 (0.75-0.91)	7.88 (6.83-9.10)
Diabetes mellitus	760	1520	2145	0.75 (0.64-0.89)	4.38 (3.66-5.26)
Baseline eGFR					
≥105	55	90	180	0.49 (0.29-0.81)	8.76 (3.19-24.04)
90-104	125	225	355	0.66 (0.49-0.88)	7.71 (4.94-12.04)

75-89	140	310	455	0.79 (0.61-1.01)	6.94 (5.18-9.31)
60-74	155	275	390	1.05 (0.80-1.38)	5.64 (4.14-7.70)
45-59	155	320	500	1.09 (0.80-1.48)	4.24 (3.07-5.87)
30-44	240	435	670	1.20 (0.83-1.73)	2.39 (1.69-3.40)
15-29	540	720	1630	1.08 (0.62-1.88)	0.90 (0.56-1.46)

Event counts rounded to nearest 5. Models adjusted for ethnicity, deprivation, rural or urban, body mass index, smoking status, baseline eGFR (with “no baseline eGFR measurement” treated as a categorical variable), previous acute kidney injury, cardiovascular diseases, diabetes mellitus, hypertension, immunosuppressive diseases, non-haematological cancer, general practice consultations in the previous year, hospital admissions in the previous five years, COVID-19 vaccination status and COVID-19 wave. Age in years. Baseline eGFR in ml/min/1.73m^2. CI = confidence interval.

Table S12

Fully-adjusted hazard ratio (HR) and adjusted rate difference estimates for kidney failure (i.e. incident dialysis, kidney transplantation or estimated glomerular filtration rate (eGFR) <15 ml/min/1.73m^2), 50% reduction in eGFR, and death after COVID-19 compared to an age-, sex- and sustainability and transformation partnership region-matched cohort, stratified by COVID-19 wave as an interaction (**Figure S7**).

	Events in COVID-19 group	Events in matched group	Fully-adjusted HR (95% CI)	P-value for interaction
Kidney failure				
February 2020 – August 2020 (wild-type)	745	550	3.77 (3.20-4.45)	<0.0001
September 2020 – June 2021 (Alpha variant)	1685	2240	1.81 (1.65-1.99)	
July 2021 – November 2021 (Delta variant)	735	1325	1.27 (1.11-1.45)	
December 2021 – March 2022 (Omicron BA.1 variant)	670	1180	1.22 (1.06-1.41)	
50% reduction in eGFR				
February 2020 – August 2020 (wild-type)	1090	1080	2.35 (2.08-2.65)	<0.0001
September 2020 – June 2021 (Alpha variant)	2445	3645	1.45 (1.35-1.56)	
July 2021 – November 2021 (Delta variant)	910	1695	1.30 (1.16-1.45)	
December 2021 – March 2022 (Omicron BA.1 variant)	900	1655	1.19 (1.07-1.34)	
Death				
February 2020 – August 2020 (wild-type)	10165	9480	3.06 (2.95-3.18)	<0.0001
September 2020 – June 2021 (Alpha variant)	22300	28050	2.03 (1.99-2.08)	
July 2021 – November 2021 (Delta variant)	6380	12290	1.33 (1.29-1.38)	
December 2021 – March 2022 (Omicron BA.1 variant)	8635	11800	1.79 (1.73-1.85)	

Models adjusted for ethnicity, deprivation, rural or urban, body mass index, smoking status, baseline eGFR (with “no baseline eGFR measurement” treated as a categorical variable), previous acute kidney injury, cardiovascular diseases, diabetes mellitus, hypertension, immunosuppressive diseases, non-haematological cancer, general practice consultations in the previous year, hospital admissions in the previous five years and COVID-19 vaccination status. Wave 4 is restricted to December 2021 to March 2022 (i.e. the end of universal access to COVID-19 testing). *P*-values for interaction obtained by likelihood ratio tests comparing models with and without interaction term. CI = confidence interval.

Table S13

Fully-adjusted hazard ratio estimates for kidney failure (i.e. incident dialysis, kidney transplantation or estimated glomerular filtration rate (eGFR) <15 ml/min/1.73m^2), 50% reduction in eGFR, and death after COVID-19 stratified by hospitalisation status compared to an age-, sex- and sustainability and transformation partnership region-matched cohort, stratified by COVID-19 wave as an interaction (**Figure S7**).

	Events in COVID-19 group		Events in matched group	Fully-adjusted hazard ratio (95% CI)	
	Non-hospitalised	Hospitalised		Non-hospitalised COVID-19	Hospitalised COVID-19
Kidney failure					
February 2020 – August 2020 (wild-type)	205	535	550	1.23 (0.94-1.60)	8.74 (6.89-11.08)
September 2020 – June 2021 (Alpha variant)	685	1000	2240	0.87 (0.76-0.98)	6.83 (5.73-8.15)
July 2021 – November 2021 (Delta variant)	390	345	1325	0.75 (0.64-0.88)	7.74 (5.61-10.67)
December 2021 – December 2022 (Omicron variants)	405	975	1455	0.81 (0.68-0.95)	8.36 (6.81-10.27)
50% reduction in eGFR					
February 2020 – August 2020 (wild-type)	475	830	1180	1.47 (1.23-1.75)	3.63 (3.06-4.31)
September 2020 – June 2021 (Alpha variant)	1400	1650	4375	0.93 (0.85-1.02)	3.09 (2.73-3.49)
July 2021 – November 2021 (Delta variant)	745	480	2250	0.98 (0.86-1.11)	3.74 (2.89-4.84)
December 2021 – December 2022 (Omicron variants)	760	1480	2610	0.93 (0.82-1.05)	3.90 (3.40-4.48)
Death					
February 2020 – August 2020 (wild-type)	4775	5390	9480	2.47 (2.35-2.60)	3.96 (3.74-4.19)
September 2020 – June 2021 (Alpha variant)	10570	11730	28050	1.33 (1.29-1.36)	4.21 (4.06-4.37)
July 2021 – November 2021 (Delta variant)	3300	3080	12290	0.85 (0.81-0.89)	4.45 (4.13-4.79)
December 2021 – December 2022 (Omicron variants)	4730	13955	16055	1.11 (1.06-1.15)	6.57 (6.31-6.83)

Event counts rounded to nearest 5. Models adjusted for ethnicity, deprivation, rural or urban, body mass index, smoking status, baseline eGFR (with “no baseline eGFR measurement” treated as a categorical variable), previous acute kidney injury, cardiovascular diseases, diabetes mellitus, hypertension, immunosuppressive diseases, non-haematological cancer, general practice consultations in the previous year, hospital admissions in the previous five years and COVID-19 vaccination status. CI = confidence interval.

Table S14

Fully-adjusted hazard ratio estimates for kidney failure (i.e. incident dialysis, kidney transplantation or estimated glomerular filtration rate (eGFR) <15 ml/min/1.73m^2) after COVID-19 stratified by levels of severity compared to an age-, sex- and sustainability and transformation partnership region-matched cohort (**Figure S8**).

	Kidney failure events in COVID-19 group	Fully-adjusted hazard ratio (95% CI)
COVID-19 non-hospitalised	1690	0.84 (0.79-0.90)
COVID-19 hospitalised ward-based	1560	3.19 (2.84-3.59)
COVID-19 hospitalised ICU	1295	47.38 (36.49-61.50)
COVID-19 hospitalised without AKI	865	2.53 (2.21-2.90)
COVID-19 hospitalised with AKI	2020	28.40 (23.63-34.13)

Event counts rounded to nearest 5. Models adjusted for ethnicity, deprivation, rural or urban, body mass index, smoking status, baseline eGFR (with "no baseline eGFR measurement" treated as a categorical variable), previous acute kidney injury, cardiovascular diseases, diabetes mellitus, hypertension, immunosuppressive diseases, non-haematological cancer, general practice consultations in the previous year, hospital admissions in the previous five years, COVID-19 vaccination status and COVID-19 wave. AKI = acute kidney injury, CI = confidence interval, ICU = intensive care unit.

Table S15

Demographic, socioeconomic and clinical characteristics for the hospitalised COVID-19 cohort and a historical hospitalised pneumonia cohort.

	Hospitalised COVID-19	Hospitalised pneumonia
Number of individuals	270675	340210
Follow-up (days), median (IQR)	332 (144-687)	333 (101-657)
Age (years), median (IQR)	69 (50-81)	76 (64-85)
Sex, n (%)		
Female	139280 (51.5)	170920 (50.2)
Male	131395 (48.5)	169290 (49.8)
Index of multiple deprivation, n (%)		
1 Most deprived	67740 (25.0)	76575 (22.5)
2	56910 (21.0)	69540 (20.4)
3	55615 (20.5)	72985 (21.5)
4	48975 (18.1)	65405 (19.2)
5 Least deprived	41435 (15.3)	55705 (16.4)
Ethnicity, n (%)		
White	197405 (72.9)	249655 (73.4)
South Asian	18660 (6.9)	11535 (3.4)
Black	6505 (2.4)	3270 (1.0)
Mixed	2470 (0.9)	1255 (0.4)
Other	3800 (1.4)	2005 (0.6)
Missing	41835 (15.5)	72490 (21.3)
Region, n (%)		
East Midlands	51985 (19.2)	63370 (18.6)
East	55730 (20.6)	71595 (21.0)
London	14680 (5.4)	16005 (4.7)
North East	17135 (6.3)	19525 (5.7)

North West	28160 (10.4)	32745 (9.6)
South East	16195 (6.0)	23185 (6.8)
South West	31780 (11.7)	47915 (14.1)
West Midlands	15060 (5.6)	15050 (4.4)
Yorkshire and The Humber	39950 (14.8)	50825 (14.9)
Urban/rural, n (%)		
Urban	220985 (81.6)	268575 (78.9)
Rural	49690 (18.4)	71630 (21.1)
Body mass index, n (%)		
<18.5 kg/m^2	7665 (2.8)	14465 (4.3)
18.5-24.9 kg/m^2	72160 (26.7)	106535 (31.3)
25.0-29.9 kg/m^2	80845 (29.9)	102990 (30.3)
30.0-34.9 kg/m^2	51515 (19.0)	56595 (16.6)
35.0-39.9 kg/m^2	23870 (8.8)	23485 (6.9)
≥40.0 kg/m^2	14655 (5.4)	12895 (3.8)
Missing	19970 (7.4)	23245 (6.8)
Smoking, n (%)		
Non-smoker	102435 (37.8)	100435 (29.5)
Current/former smoker	164910 (60.9)	237375 (69.8)
Missing	3330 (1.2)	2395 (0.7)
Past medical history		
Baseline eGFR (ml/min/1.73m^2), median (IQR)	74.8 (56.1-90.4)	69.8 (52.0-85.6)
Previous acute kidney injury, n (%)	38255 (14.1)	38110 (11.2)
Cardiovascular diseases, n (%)	95525 (35.3)	141620 (41.6)
Diabetes mellitus, n (%)	84405 (31.2)	95125 (28.0)
Hypertension, n (%)	138245 (51.1)	202510 (59.5)
Immunosuppressive diseases, n (%)	20720 (7.7)	29895 (8.8)
Non-haematological cancer, n (%)	54085 (20.0)	86425 (25.4)

GP consultations previous year, median (IQR)	11 (5-20)	10 (3-19)
Hospital admissions previous 5 years, n (%)		
0	62095 (22.9)	103915 (30.5)
1	43655 (16.1)	67420 (19.8)
>1	164930 (60.9)	168875 (49.6)
COVID-19 vaccination status, n (%)		
Unvaccinated	123210 (45.5)	N/A
1 vaccine dose	12200 (4.5)	N/A
2 vaccine doses	36315 (13.4)	N/A
3 vaccine doses	66765 (24.7)	N/A
4 vaccine doses	32185 (11.9)	N/A
COVID-19 wave, n (%)		
February 2020 – August 2020 (wild-type)	26255 (9.7)	N/A
September 2020 – June 2021 (Alpha variant)	76820 (28.4)	N/A
July 2021 – November 2021 (Delta variant)	34490 (12.7)	N/A
December 2021 – December 2022 (Omicron variants)	133105 (49.2)	N/A

Counts rounded to nearest 5. eGFR = estimated glomerular filtration rate, GP = general practice, IQR = interquartile range, N/A = not applicable.

Table S16

Fully-adjusted hazard ratio estimates for kidney failure (i.e. incident dialysis, kidney transplantation or estimated glomerular filtration rate (eGFR) <15 ml/min/1.73m^2), 50% reduction in eGFR, and death after COVID-19 hospitalisation compared to a historical cohort after hospitalisation for pneumonia, over all time periods and by specific follow-up periods (in days since index date, i.e., 28 days after first COVID-19 infection record) (**Figure S10**).

	Events in COVID-19 group	Events in pneumonia group	Fully-adjusted hazard ratio (95% CI)
Kidney failure			
Overall	3830	6630	0.67 (0.64-0.71)
0-29 days	195	340	0.75 (0.61-0.93)
30-89 days	390	545	0.90 (0.76-1.06)
90-179 days	370	605	0.77 (0.66-0.91)
180+ days	870	1565	0.79 (0.71-0.88)
50% reduction in eGFR			
Overall	5180	8660	0.78 (0.75-0.82)
0-29 days	1560	2800	0.62 (0.58-0.67)
30-89 days	795	1195	0.86 (0.77-0.96)
90-179 days	755	1195	0.84 (0.75-0.93)
180+ days	2040	3425	0.93 (0.87-0.99)
Death			
Overall	46920	97925	0.79 (0.78-0.80)
0-29 days	13255	22365	0.92 (0.89-0.94)
30-89 days	10205	19840	0.79 (0.77-0.81)
90-179 days	7240	15190	0.76 (0.73-0.79)
180+ days	15785	39685	0.72 (0.71-0.74)

Event counts rounded to nearest 5. Models adjusted for ethnicity, deprivation, rural or urban, body mass index, smoking status, baseline eGFR, previous acute kidney injury, cardiovascular diseases, diabetes mellitus, hypertension, immunosuppressive diseases, non-haematological cancer, general practice consultations in the previous year, hospital admissions in the previous five years and calendar month. CI = confidence interval.

Appendix S1

Information governance and ethical approval

NHS England is the data controller of the NHS England OpenSAFELY COVID-19 Service; TPP is the data processor; all study authors using OpenSAFELY have the approval of NHS England (https://digital.nhs.uk/coronavirus/coronavirus-covid-19-response-information-governance-hub/the-nhs-england-opensafely-covid-19-service-privacy-notice). This implementation of OpenSAFELY is hosted within the [TPP environment which is accredited to the ISO 27001 information security standard and is NHS IG Toolkit compliant (https://digital.nhs.uk/data-and-information/looking-after-information/data-security-and-information-governance/data-security-and-protection-toolkit).

Patient data has been pseudonymised for analysis and linkage using industry standard cryptographic hashing techniques; all pseudonymised datasets transmitted for linkage onto OpenSAFELY are encrypted; access to the NHS England OpenSAFELY COVID-19 service is via a virtual private network (VPN) connection; the researchers hold contracts with NHS England and only access the platform to initiate database queries and statistical models; all database activity is logged; only aggregate statistical outputs leave the platform environment following best practice for anonymisation of results such as statistical disclosure control for low cell counts (https://digital.nhs.uk/data-and-information/information-standards/information-standards-and-data-collections-including-extractions/publications-and-notifications/standards-and-collections/isb1523-anonymisation-standard-for-publishing-health-and-social-care-data).

The service adheres to the obligations of the UK General Data Protection Regulation (UK GDPR) and the Data Protection Act 2018. The service previously operated under notices initially issued in February 2020 by the Secretary of State under Regulation 3(4) of the Health Service (Control of Patient Information) Regulations 2002 (COPI Regulations), which required organisations to process confidential patient information for COVID-19 purposes; this set aside the requirement for patient consent (https://www.gov.uk/government/publications/coronavirus-covid-19-notification-of-data-controllers-to-share-information/coronavirus-covid-19-notice-under-regulation-34-of-the-health-service-control-of-patient-information-regulations-2002-general--2). As of 1 July 2023, the Secretary of State has requested that NHS England continue to operate the Service under the COVID-19 Directions 2020 (https://digital.nhs.uk/about-nhs-digital/corporate-information-and-documents/directions-and-data-provision-notices/secretary-of-state-directions/covid-19-public-health-directions-2020). In some cases of data sharing, the common law duty of confidence is met using, for example, patient consent or support from the Health Research Authority Confidentiality Advisory Group (https://www.hra.nhs.uk/about-us/committees-and-services/confidentiality-advisory-group/).

Taken together, these provide the legal bases to link patient datasets using the service. GP practices, which provide access to the primary care data, are required to share relevant health information to support the public health response to the pandemic, and have been informed of how the service operates.

Appendix S2

Justification for study design decisions, potential biases and how biases have been addressed

Design decision	Justification	Potential bias introduced by design decision	Mitigation of bias
Exclusion of individuals with a pre-existing code for dialysis or kidney transplantation, or eGFR <15ml/min/1.73m^2, prior to first COVID-19 record (or matching date for matched comparators).	We aimed to reliably capture new-onset outcomes in people without pre-existing kidney failure. We therefore excluded individuals with historical codes for dialysis.	Selection bias – without access to UK Renal Registry data, our definition of pre-existing kidney failure will have resulted in the exclusion of people who previously received only short-term dialysis for acute kidney injury that subsequently recovered, or had a calculated eGFR <15 ml/min/1.73m^2 due to acute kidney injury which subsequently recovered. As these groups may also be at high risk of severe COVID-19 and COVID-19-related acute kidney injury, this selection bias may have resulted in an underestimation of the effect of COVID-19 on long-term kidney outcomes.	
Exclusion of individuals who died within the first 28 days of COVID-19 infection (i.e. before the index date).	A high proportion of individuals who required dialysis as a complication of COVID-19 died during acute illness. Given that our research question focused on investigating long-term kidney outcomes, inclusion of these individuals would have resulted in an overestimation of these outcomes.		
Inclusion of individuals who had a code for dialysis in first 28 days after first recording of COVID-19 (i.e. before the index date).	Our research question aimed at investigating long-term kidney outcomes. Individuals with severe COVID-19-related acute kidney injury requiring dialysis are likely to be among those at the highest risk of developing long-term kidney complications. Additionally, including these individuals enabled us to determine the true overall burden of dialysis associated with COVID-19 infection amongst survivors.	Misclassification of the outcome (overestimation) in some people who received dialysis for severe acute kidney injury who then subsequently recovered kidney function.	We undertook sensitivity analyses excluding individuals with a code for dialysis in the first 28 days after first recording of COVID-19 (i.e. before the index date).
Dialysis events recorded in the first 28 days after COVID-19 diagnosis (or equivalent for matched comparators) were counted as outcomes. We counted these as outcomes as occurring on day 1 of study follow-up (i.e. 29 days after first recording of COVID-19 infection or matching).	If we had excluded these events, as people may not have had a subsequent code for dialysis despite continuing beyond 28 days, we would have underestimated our outcome, particularly considering the high intermediate-term mortality risk in this group (e.g., within three months).<br><br>We also considered acute dialysis in survivors to be an important indicator of long-term health resource needs that would otherwise be missed.	Misclassification of the outcome (overestimation) in some people who received dialysis for severe acute kidney injury who then subsequently recovered kidney function.	We undertook sensitivity analyses only counting recorded dialysis events from after the first 28 days of COVID-19 diagnosis (i.e. after the index date).

eGFR = estimated glomerular filtration rate

Appendix S3

Description of covariates

Covariate	Definition/components	Data source	Categories
Age		Primary care	Whole year
Sex		Primary care	Female, male
Index of multiple deprivation	Divided into quintiles	Primary care	1 Most deprived, 2, 3, 4, 5 Least deprived
Ethnicity		Primary care	White, South Asian, Black, Mixed, Other, Missing
Region of England		Primary care	East Midlands, East, London, North East, North West, South East, South West, West Midlands, Yorkshire and The Humber
STP		Primary care	31 STPs
Urban/rural		Primary care	Binary
Body mass index	Calculated from most recent height and weight record	Primary care	<18.5 kg/m^2, 18.5-24.9 kg/m^2, 25.0-29.9 kg/m^2, 30.0-34.9 kg/m^2, 35.0-39.9 kg/m^2, ≥40.0 kg/m^2
Smoking		Primary care	Never smoked, Current/former smoker, Missing
Baseline eGFR	Mean eGFR from 18 months preceding COVID-19 diagnosis date/matching date, calculated from serum creatinine (CKD-EPI equation excluding ethnicity adjustment)	Primary care	≥105 ml/min/1.73m^2, 90-104 ml/min/1.73m^2, 75-89 ml/min/1.73m^2, 60-74 ml/min/1.73m^2, 45-59 ml/min/1.73m^2, 30-44ml/min/1.73m^2,15-29 ml/min/1.73m^2, No baseline eGFR measurement
Previous acute kidney injury		SUS	Binary
Cardiovascular diseases	Atrial fibrillation, heart failure, myocardial infarction, peripheral vascular disease, stroke	Primary care	Binary
Diabetes mellitus		Primary care	Binary
Hypertension		Primary care	Binary
Immunosuppressed	Haematological cancer, human immunodeficiency virus, rheumatoid arthritis, systemic lupus erythematosus	Primary care	Binary
Non-haematological cancer		Primary care	Binary
GP consultations previous year		Primary care	0, 1-2, 3-9, >9
Hospital admissions previous 5 years		SUS	0, 1, >1
COVID-19 vaccination status	Number of vaccinations ≥15 days apart and ≥7 days prior to the COVID-19 diagnosis/matching date	Primary care	Unvaccinated, 1 vaccine dose, 2 vaccine doses, 3 vaccine doses, 4 vaccine doses
COVID-19 wave	COVID-19 diagnosis/matching date	Primary care, SUS	February 2020 – August 2020 (wild-type), September 2020 – June 2021 (Alpha variant), July 2021 – November 2021 (Delta variant), December 2021 – December 2022 (Omicron variants)

eGFR = estimated glomerular filtration rate, STP = sustainability and transformation partnership, SUS = Secondary Uses Services

Codelists available at https://github.com/opensafely/post-covid-kidney-outcomes/tree/main/codelists

Appendix S4

Guidance for Reporting Involvement of Patients and Public 2 short form (GRIPP2-SF) checklist

<table>
<tr><td>Section and topic</td><td>Item</td></tr>
<tr><td>1. Aim

Report the aim of the study</td><td>We aimed to comprehensively investigate the burden of long-term kidney outcomes following COVID-19 infection up to December 2022 using large, nationwide electronic health records.</td></tr>
<tr><td>2. Methods

Provide a clear description of the methods used for PPI in the study</td><td>We consulted several patients and members of the public with and without kidney diseases and people who had and had not had COVID-19 through local and national kidney patient groups, the NIHR People in Research, the COVID-19 Research Involvement Group, national charities and community groups. Many of those who came forward had no previous experience of PPI. Discussions with individuals provided insight into research and engagement priorities and supported a Fellowship application to NIHR which included funding for ongoing PPI activities.

After successful obtaining a Fellowship, we organised a series of online activities introducing PPI, discussing electronic health record research and experiences of COVID-19 and kidney disease. These meetings involved breakout rooms facilitated by the research team to be able to identify research priorities and determine how these could be achieved within the OpenSAFELY platform. Partners were also able to highlight concerns and ideas to be able to connect further with communities and build trust about the use of electronic health records in research.

Four PPI members were invited to form the steering committee of the study. This group had a range of backgrounds and experience. This group provided feedback on results, supported dissemination, and set further priorities. There are plans for ongoing dissemination include community group discussions and video content.</td></tr>
<tr><td>3. Results

Outcomes—Report the results of PPI in the study, including both positive and negative outcomes</td><td>PPI contributed to the study in several ways:

• Questionnaire to understand skills and level of involvement of different PPI partners
• Prioritisation of investigating long-term kidney outcomes after COVID-19
• Ensuring diverse representation and identifying evidence of inequalities
• Feeding back on study results
• Designed research posters and slides for presentation at national conferences
• Presentation at national conference to clinicians
• Setting further research priorities within Fellowship and for future funding
• Writing accessible lay summaries
• Moving towards additional layers of involvement aiming to reach more of the general public
• Invitations to discuss PPI strategy across departments at London School of Hygiene & Tropical Medicine</td></tr>
</table>

**4. Discussion**<br><br>Outcomes—Comment on the extent to which PPI influenced the study overall. Describe positive and negative effects	PPI influenced several important aspects of this study including funding application, setting priorities and determining a dissemination strategy. This was especially useful given the limited experience of PPI in electronic health record research, as it provided an opportunity to better understand how NHS users feel about their data and its use for research in the broader public interest.<br><br>There is limited awareness in the public about kidney-related complications of COVID-19, as well as the heightened risks for patients with pre-existing kidney disease and ethnic differences in outcomes. Under the guidance of our PPI partners, we plan to address this in our future engagement work.
**5. Reflections**<br><br>Critical perspective—Comment critically on the study, reflecting on the things that went well and those that did not, so others can learn from this experience	Our approach was dynamic and evolved as the study progressed under the direction PPI partners and supported by NIHR funding. The study benefited from using a range of approaches to consult PPI partners including one-to-one conversations, online group activities, questionnaires and meetings. Moving forward, we plan to engage our research more directly with community groups and the general public. The main constraint limiting the full potential of PPI in our work has been the lack of time and resources for administrative support.

NHS = National Health Service, NIHR = National Institute for Health and Care Research, PPI = patient and public involvement

Appendix S5

OpenSAFELY patient and public involvement

OpenSAFELY has involved patients and the public in various ways: we developed a public website that provides a detailed description of the platform in language suitable for a lay audience (**https://opensafely.org**); we have participated in two citizen juries exploring public trust in OpenSAFELY; we have co-developed an explainer video (**https://www.opensafely.org/about/);** we have patient representation who are experts by experience on our OpenSAFELY Oversight Board; we have partnered with Understanding Patient Data to produce lay explainers on the importance of large datasets for research; we have presented at various online public engagement events to key communities (e.g., Healthcare Excellence Through Technology; Faculty of Clinical Informatics annual conference; NHS Assembly; HDRUK symposium); and more. To ensure the patient voice is represented, we are working closely to decide on language choices with appropriate medical research charities (e.g., Association of Medical Research Charities). We will share information and interpretation of our findings through press releases, social media channels, and plain language summaries.

Appendix S6

Sources of collider bias

Potential sources of collider bias are described below. In the directed acyclic graphs (right): = exposure, = outcome, = factor on which selection is conditioned, and = an alternative cause for the outcome

Death due to COVID-19

Potential matched contemporary comparators who die from COVID-19 before they can be matched include those are higher susceptibility of kidney failure. With time, this results in a depletion of the most susceptible potential matched contemporary comparators resulting in overestimation of the effect in those who survive COVID-19.

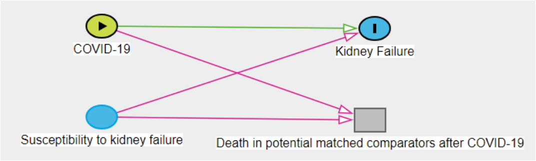

Death due to COVID-19 hospitalisation

(Compared to pre-pandemic pneumonia hospitalisation)

Individuals with susceptibility for kidney failure who would have survived pneumonia pre-pandemic to then be able to develop kidney failure, are more likely to have died from COVID-19 hospitalisation. While we adjusted for several potential factors associated with susceptibility for kidney failure, there is likely persistent residual confounding related to the severity of comorbidities (e.g. diabetes, cardiovascular disease) and frailty.

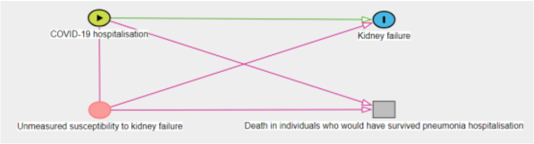

COVID-19 testing

Individuals who are more susceptible to kidney failure may be more likely to undergo COVID-19 testing due to heightened risk of severe disease.

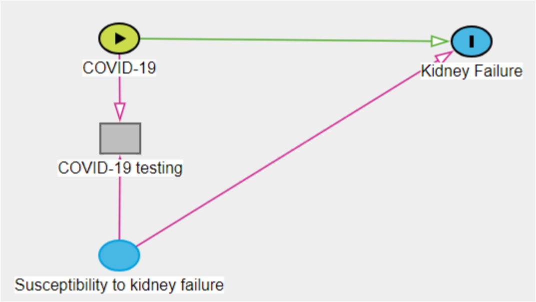

Supplement: Supplementary Figures and Tables [file mmc2.pdf]
